# Enantiospecific Alkynylation of Alkylboronic Esters

**DOI:** 10.1002/anie.201600599

**Published:** 2016-03-02

**Authors:** Yahui Wang, Adam Noble, Eddie L. Myers, Varinder K. Aggarwal

**Affiliations:** ^1^ School of ChemistryUniversity of BristolCantock's CloseBristolBS8 1TSUK

**Keywords:** alkynes, alkynylation, boronic esters, synthetic methods

## Abstract

Enantioenriched secondary and tertiary alkyl pinacolboronic esters undergo enantiospecific deborylative alkynylation through a Zweifel‐type alkenylation followed by a 1,2‐elimination reaction. The process involves use of α‐lithio vinyl bromide or vinyl carbamate species, for which application to Zweifel‐type reactions has not previously been explored. The resulting functionalized 1,1‐disubstituted alkenes undergo facile base‐mediated elimination to generate terminal alkyne products in high yield and excellent levels of enantiospecificity over a wide range of pinacolboronic ester substrates. Furthermore, along with terminal alkynes, internal and silyl‐protected alkynes can be formed by simply introducing a suitable carbon‐ or silicon‐based electrophile after the base‐mediated 1,2‐elimination reaction.

Stereoselective methods for the synthesis of alkynes^1^, ^2^ have received renewed interest as a result of their considerable synthetic utility across an array of modern reactions, including click reactions with azides,^3^ gold‐catalyzed cycloisomerization,^4^ and enyne/diyne metathesis.^5^ Alkyne‐substituted stereocenters can be installed with high enantioselectivity by a number of methods, including the asymmetric addition of acetylides to carbonyls,^6^ transformation of chiral aldehydes or ketones into the corresponding alkynes,^7^ and copper‐catalyzed allylic substitution reactions.^8^ As an alternative, we considered stereospecific conversion of chiral alkylboronic esters into alkynes because alkylboronic esters themselves are versatile intermediates that can be readily prepared with high enantioselectivity using a variety of methods,^9^ including by a lithiation–borylation method developed within our laboratory.^10^

The Suzuki–Miyaura reaction could potentially be used to transform boronic esters into alkynes in conjunction with an alkynyl halide. However, the use of chiral boronic acids/esters in such cross‐coupling reactions is not known; the only reported examples are those that utilize primary sp^3^‐, sp^2^‐, and sp‐type boron species, which are compounds that undergo facile transmetalation.^11^, ^12^ Another attractive method involves electrophile‐induced 1,2‐migration of an alkynyl boronate followed by deboronation (Scheme 1 A). However, this approach is only applicable to symmetric trialkylboranes (BR_3_)^13^, ^14^ and borinic esters (BR_2_OR),^15^ which suffer from a number of drawbacks, including difficulty in preparing an enantioenriched form, poor stability, and the poor atom economy of subsequent transformations (two R groups are wasted in borane transformations). As such, the majority of examples involve simple, non‐chiral boron reagents and the only enantiospecific examples utilize secondary borinic esters that require lengthy syntheses.15d,15e Furthermore, these methods are not applicable to direct synthesis of terminal alkynes and cannot be used to access alkynes with α‐quaternary stereocenters.

**Scheme 1:**
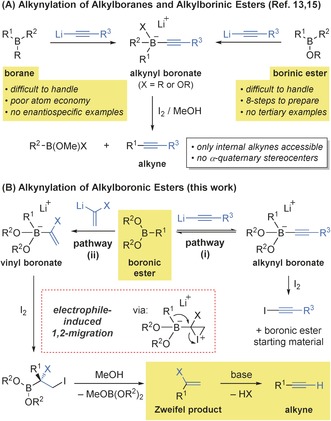
Alkynylation of alkyl boron species.

Compared to boranes and borinic esters, boronic esters [BR(OR)_2_] are atom‐economic substrates, which are easier to prepare and handle; however, they do not undergo alkynylation reactions owing to the reversibility of alkynyl boronate complex formation.15c Here, the addition of electrophiles leads to trapping of the acetylide and recovery of the boronic ester starting material (Scheme 1 B, pathway (i)).^16^ In contrast, vinyl boronate complexes of alkylboronic esters are much more stable with respect to fragmentation and undergo facile electrophile‐induced 1,2‐migration and deboronation by the so‐called Zweifel olefination.^17^ We questioned whether alkynes could be generated using a variant of this transformation by implementing suitably functionalized vinyl anions that would allow the resulting substituted alkene (Zweifel product) to undergo elimination and thereby unmask the alkyne (Scheme 1 B, pathway (ii)).^18^ Such a method would enable the use of readily available alkylboronic esters to prepare chiral alkynes that are not easily accessed using established methods. Herein, we report the development of an alkynylation method that enables enantiospecific transformation of structurally diverse secondary and tertiary pinacolboronic esters into terminal and internal alkynes.

To test our strategy, we started with commercially available vinyl bromide **2 a**, which can be lithiated at the α‐position with LDA at low temperature.^19^ Initially, we focused on optimizing conditions for the preparation of bromoalkene intermediate **3 a** (Table 1). Treatment of a THF solution of vinyl bromide (**2 a**, 1.3 equiv) and secondary boronic ester **1 a** at −78 °C with LDA (1.3 equiv) presumably generated vinylboronate **5**, which, after addition of a methanolic solution of I_2_ (1.5 equiv) and subsequent warming to room‐temperature, gave the required alkene **3 a** together with starting material **1 a** in a circa 1:1 ratio (entry 1, Table 1). Switching the solvent to less coordinating TBME (*tert*‐butyl methyl ether) or Et_2_O (albeit with some THF present owing to **2 a** being added as a 1.0 m solution in THF) led to increased conversion to **3 a**. However, small amounts of insertion product **4 a**, a compound formed by 1,2‐migration of vinylboronate **5**, were also detected (entries 2 and 3, Table 1).^20^ The significant amounts of **1 a** recovered (likely because of the instability of 1,1‐lithiobromoethene)19b,19d,19e and competing rearrangement of **5** into **4 a**, prompted us to conduct the transformation at a lower temperature (−95 °C) and increase the relative amounts of vinyl bromide and LDA used (2.0 equiv). Under these conditions, conversion into **3 a** was improved and **4 a** was not detected (entry 4, Table 1). Finally, conducting the reaction in Et_2_O, whilst adding both vinyl bromide and LDA as solutions in THF^21^ (Et_2_O/THF 3:2) gave a 96 % conversion to **3 a** (entry 6, Table 1). Use of this mixture of solvents was superior to exclusive use of either THF or Et_2_O, the latter leading to complete recovery of starting material (entries 5 and 7, Table 1).

**Table 1:** Optimization of conditions for generating 1,1‐bromoalkylalkenes from alkylboronic esters. 
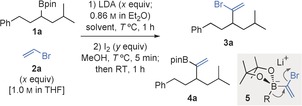

Entry^[a]^	LDA/**2 a** (*x* equiv)	*T* [°C]	Solvent	I_2_ (*y* equiv)	**3 a**:**1 a**:**4 a** ^[b]^
1	1.3	−78	THF	1.5	49:51:0
2	1.3	−78	TBME	1.5	79:18:3
3	1.3	−78	Et_2_O	1.5	81:14:5
4	2.0	−95	Et_2_O	2.2	87:13:0
5^[c]^	2.0	−95	THF	2.2	91:9:0
6^[c]^	2.0	−95	Et_2_O	2.2	96:4:0
7^[d]^	2.0	−95	Et_2_O	2.2	0:100:0

[a] Reactions were conducted using **1 a** (0.15 mmol) in solvent (1.0 mL; not including the solvent used to add **2 a** and LDA); I_2_ was added as a solution in MeOH (2.0 mL). [b] Ratio determined by GC‐MS analysis of the crude reaction mixture. [c] Using LDA (0.86 m in THF). [d] Using **2 a** (1.0 m in Et_2_O). TBME=*tert*‐butyl methyl ether; pin=pinacol; LDA=lithium diisopropylamide.

Once an efficient process was established for generating the 1,1‐bromoalkylalkene **3 a**, we investigated a variety of conditions for effecting dehydrobromination. Our attempts to unmask the alkyne in situ, by addition of basic reagents after treatment with I_2_/MeOH, only led to isolation of **3 a**. However, subsequent work‐up and treatment of a solution of crude **3 a** with either TBAF (5.0 equiv, DMF, 60 °C, 1 h)^22^ or LDA (2.6 equiv, THF, −78 °C to RT)^23^ led to isolation of alkyne **6 a** in good yield and with complete enantiospecificity (Scheme 2). Using the optimized conditions, we converted a variety of enantioenriched secondary boronic esters **1** into their corresponding alkynyl derivatives **6** (Scheme 2). The transformation occurred cleanly in the presence of cyclopropyl, alkene, azide, electron‐rich aryl, silyl ether, and *tert*‐butyl ester functional groups, and showed essentially complete enantiospecificity. The alkynylation method was also successfully applied to hindered boronic ester **1 h**, derived from menthol, leading to alkynes **6 h/6 h′**.

**Scheme 2:**
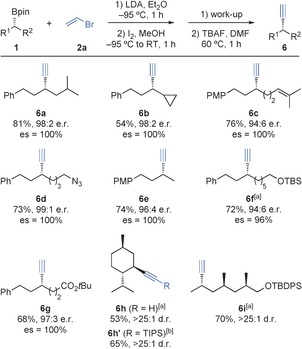
Scope of alkynylation of secondary boronic esters. Reactions were carried out with **1** (0.30 mmol), **2 a** (1.0 m in THF, 0.60 mmol), LDA (0.86 m in THF, 0.60 mmol), I_2_ (0.66 mmol), and TBAF⋅3 H_2_O (1.5 mmol). [a] LDA (0.86 m in THF, 0.75 mmol) was used instead of TBAF for dehydrobromination. [b] TIPSCl was added after dehydrobromination with LDA. The higher yield of **6 h′** was attributed to the volatility of **6 h**. TBAF=tetra‐*n*‐butylammonium fluoride; TBDPS=*tert*‐butyldiphenylsilyl; TBS=*tert*‐butyldimethylsilyl; TIPS=triisopropylsilyl; PMP=*para*‐methoxyphenyl.

Alkynylation of tertiary boronic esters was more challenging. Using conditions optimized for the alkenylation of secondary boronic esters, only 42 % of substrate **1 j** was converted into vinyl bromide **3 j**, and significant amounts of insertion product **4 j** and starting material were also detected (10 % and 48 %, respectively; Scheme 3). Presumably, increased steric hindrance close to the boron atom slows both vinyl boronate formation and subsequent electrophilic activation, thus allowing decomposition of the vinyllithium reagent and 1,2‐migration of the vinyl boronate to become competing reaction pathways. In an effort to diminish side‐reactions, we investigated the use of vinyl carbamate **2 b** (readily prepared from THF, ^*n*^BuLi, and CbCl (Cb=*N*,*N‐* ^*i*^Pr_2_NCO))^24^ in place of vinyl bromide **2 a**. We anticipated that the corresponding vinyllithium species would be more stable with respect to decomposition, and that the poorer leaving‐group ability of the carbamate would engender slower 1,2‐migration and faster electrophilic activation of the corresponding vinyl boronate. Indeed, treating a THF solution of vinyl carbamate **2 b** and tertiary boronic ester **1 j** with LDA (−78 °C, 1 h) followed by I_2_/MeOH gave the 1,1‐*O*‐carbamoylalkylalkene **3 j′** with excellent conversion (Scheme 3).

**Scheme 3:**
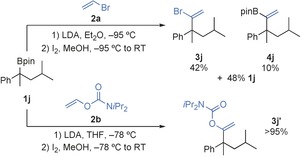
Zweifel reaction of tertiary benzylic boronic esters.

Dehydro‐*O*‐carbamoylation was effected using either ^*t*^BuLi/Et_2_O or LDA/THF to give the corresponding alkyne derivative **6 j** in good yield (89 % and 88 % yield, respectively, from **1 j** in 100 % es; Scheme 4). Other enantioenriched tertiary boronic esters, including alkene‐bearing, diaryl, and non‐benzylic substrates, were converted into their alkyne derivatives in good yield and with complete enantiospecificity (**6 k**–**m**, Scheme 4). Sterically hindered secondary boronic esters, such as cyclopropyl substrate **1 b** and cholesterol derivative **1 o**, were transformed into the corresponding alkyne derivatives **6 b** and **6 o** 25 in good yield and enantio‐/diastereospecificity using the vinyl carbamate technique. By comparison, boronic esters **1 b** and **1 o** produced low to moderate yields when vinyl bromide was used as the reagent. Pleasingly, double alkynylation of 1,2‐bis(boronic ester) **1 n** was also achieved to give 1,2‐diyne **6 n** in high yield and enantiospecificity. However, application of the alkynylation procedure to allylic boronic ester **1 p** failed because the intermediate vinyl boronate complex reacted with iodine as an allylic metal reagent, leading to fragmentation.10f

**Scheme 4:**
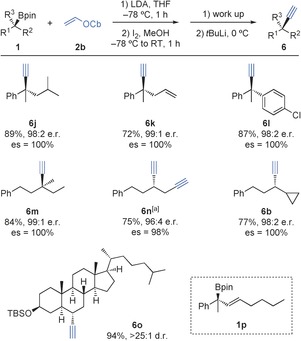
Alkynylation of tertiary and hindered secondary boronic esters. Reactions were carried out with **1** (0.3 mmol), **2 b** (0.4 mmol), LDA (0.4 mmol), I_2_ (0.4 mmol), and ^*t*^BuLi (0.75 mmol). [a] **2 b** (1.2 mmol) and LDA (1.2 mmol) were used.

The alkynylation of secondary benzylic boronic esters presented additional challenges owing to the enhanced acidity of the sp^3^ benzylic center. Under the optimized conditions enantioenriched substrate **1 q** was converted into vinyl carbamate intermediate **3 q′** in excellent yield. However, subsequent elimination with ^*t*^BuLi resulted in significant racemization, with the alkyne product **6 q** being formed in 78 % yield but with only 22 % es (entry 1, Table 2). We hypothesized that the excess ^*t*^BuLi present after elimination resulted in post‐reaction racemization and that this process could be prevented by reducing the amount of base used. Indeed, reducing the stoichiometry of ^*t*^BuLi from 2.5 to 1.1 equivalents led to much improved enantiospecificity (essentially complete), albeit with a concomitant reduction in yield (entries 1–4, Table 2). Interestingly, use of LDA resulted in considerably higher levels of racemization (entries 5 and 6, Table 2).

**Table 2:** Alkynylation of secondary benzylic boronic esters. 
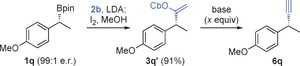

Entry^[a]^	Base (*x* equiv)		conv. [%]^[b]^	yield [%]^[b]^	e.r.^[c]^	es [%]
1	^*t*^BuLi	(2.5)	>99	78	61:39	22
2	^*t*^BuLi	(2.0)	>99	70	76:24	53
3	^*t*^BuLi	(1.5)	75	60	95:5	92
4	^*t*^BuLi	(1.1)	60	43 (37)	98:2	98
5	LDA	(2.5)	>99	87	50:50	0
6	LDA	(1.1)	49	39	63:37	27

[a] The elimination reactions were conducted using **3 q′** (0.25 mmol) in Et_2_O (2.5 mL) at −78 °C, then allowed to warm to 0 °C for 0.5 h. [b] Determined by ^1^H NMR spectroscopy using 1,3,5‐trimethoxybenzene as an internal standard. Number in parentheses shows the yield of isolated product after column chromatography. [c] Determined by chiral‐phase GC.

Finally, we wished to demonstrate the versatility of the alkynylation method by extending it to the synthesis of internal and protected alkynes. This was achieved by taking advantage of the acetylide intermediate, which formed upon elimination of bromide or carbamate en route to the alkyne and could be trapped with a variety of electrophiles. For example, carbon electrophiles produced internal alkynes **7** and **8**, whereas silyl chlorides generated alkyne **9**; all in very high yield (Scheme 5).

**Scheme 5:**
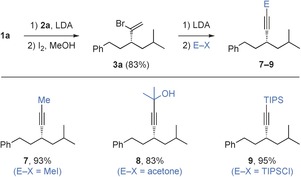
Trapping of intermediate acetylides with electrophiles.

In summary, enantioenriched secondary and tertiary boronic esters can be alkynylated in good yield and with high levels of enantiospecificity using a method involving a Zweifel‐type olefination followed by a 1,2‐elimination reaction. Either vinyl carbamates or vinyl bromides can be employed; the former demonstrates a broader substrate scope (applicable to secondary and tertiary boronic esters), whereas the latter can be eliminated using mild base (TBAF). Owing to the variety of functional groups into which alkynes can be transformed, we believe that these methods significantly contribute to the realization of a future where most organic molecules could be prepared from boronic ester building blocks.

## Supporting information

As a service to our authors and readers, this journal provides supporting information supplied by the authors. Such materials are peer reviewed and may be re‐organized for online delivery, but are not copy‐edited or typeset. Technical support issues arising from supporting information (other than missing files) should be addressed to the authors.

Supplementary
