# Supplementary material for: Enantiospecific Alkynylation of Alkylboronic Esters
Source: Angew Chem Int Ed Engl. 2016 Mar 2;55(13):4270–4. doi: 10.1002/anie.201600599 (PMC4804747; doi:10.1002/anie.201600599)

Angewandte Chemie
Eine Zeitschrift der Gesellschaft Deutscher Chemiker

Supporting Information

Enantiospecific Alkynylation of Alkylboronic Esters

*Yahui Wang, Adam Noble, Eddie L. Myers, and Varinder K. Aggarwal**

anie_201600599_sm_miscellaneous_information.pdf

Supplementary Information for

Contents

1. General Experimental 2
2. Reaction Optimization 4
3. Alkynylation General Procedures........ 5
4. Product Characterization (From Vinyl Bromide)........ 8
5. Product Characterization (From Vinyl Carbamate)........ 16
6. Synthesis of Internal Alkynes from Vinyl Bromide 3a 23
7. ^{1}H and ^{13}C NMR spectra 26

1. General Experimental

1.1 Solvents and Reagents

All air and water-sensitive reactions were carried out in flame-dried glassware under a nitrogen atmosphere using standard Schlenk manifold techniques. Bulk solutions were evaporated under reduced pressure using a Büchi rotary evaporator. All solvents were commercially supplied or provided by the communal stills of the School of Chemistry, University of Bristol. Petroleum ether refers to the fraction collected between 40 – 60 °C. *n*-BuLi was purchased from Acros. The molarity of organolithium solutions was determined by titration using *N*-benzyl benzamide as an indicator.[1] Vinyl bromide (1.0 M in THF solution) and all other reagents were purchased from commercial sources and used as received. Lithium diisopropylamide (LDA) solutions (0.86 M) were freshly prepared using diisopropylamine (0.603 mL, 4.30 mmol), *n*-BuLi (1.6 M in hexanes, 2.67 mL, 4.30 mmol) and THF or Et_2O (1.71 mL). Cryogenic temperatures were achieved used the following cold baths: acetone/CO_2 (–78 °C); MeOH/N_2 (–95 °C); EtOH/N_2 (–110 °C).

1.2 Chromatography and Spectroscopy

Flash column chromatography (FCC) was carried out using silica gel LC60A-40 (63 μm). All reactions were followed by thin-layer chromatography (TLC) when practical, using Merck Kieselgel 60 F_{254} fluorescent treated silica which was visualised under UV light or by staining with aqueous basic potassium permanganate or an ethanolic solution of phosphomolybdic acid.

^{1}H and ^{13}C NMR spectra were recorded using Jeol ECS 300 MHz, Jeol ECS 400 MHz, Varian VNMR 400 MHz and Varian VNMR 500 MHz spectrometers. Chemical shifts (δ) are given in parts per million (ppm), and coupling constants (*J*) are given in Hertz (Hz). The ^{1}H NMR spectra are reported as follows: ppm (multiplicity, coupling constants, number of protons).

High resolution mass spectra (**HRMS**) were recorded on a VG Analytical Autospec by Electron Ionisation (EI) or Chemical Ionisation (CI) or on a Brüker Daltonics Apex IV by Electrospray Ionisation (ESI). **IR spectra** were recorded on a Perkin Elmer Spectrum One FT-IR as a thin film. Only selected absorption maxima (ν_{max}) are reported in wavenumbers (cm^{-1}). **Melting points** were recorded in degrees Celsius (°C),

[1] A. F. Burchat, J. M. Chong, N. Nielsen, *J. Organomet. Chem.* **1997**, *542*, 281-283.

using a Kofler hot-stage microscope apparatus and are reported uncorrected. **Optical rotation** ($[\alpha]_D^T$) was measured on a Bellingham and Stanley Ltd. ADP220 polarimeter and is quoted in (° ml)(g dm)$^{-1}$. **Chiral HPLC** was performed on a HP Agilent 1100 with a Chiralpak IA and IB column and monitored by DAD (Diode Array Detector). **Chiral GC** was performed on an Agilent 7890A using Chiraldex DM 120 (30 m × 0.25 mm × 0.25 μm) and Chiraldex DP 120 (30 m × 0.25 mm × 0.25 μm). **GC-MS** was performed on an Agilent 7820A using a HP-5MS UI column (30 m × 0.25 mm × 0.25 μm). Enantiospecificity: **Es** = 100 × (ee product) / (ee reactant).

1.3 Naming of compounds

Compound names are those generated by ChemBioDraw 13.0 software (PerkinElmer), following the IUPAC nomenclature.

1.4 Synthesis of Starting Materials

All of the boronic esters used in this paper have been reported previously and were prepared according to the literature: **1a**[2i], **1b**[2a], **1c**[2a], **1d**[2a], **1e**[2j], **1f**[2a], **1g**[2a], **1h**[2h], **1i**[2k], **1j**[2d], **1k**[2b], **1l**[2b], **1m**[2c], **1n**[2f,g], **1o**[2d], **1p**[2l], **1q**[2e].

[2] (a) C. Sandford, R. Rasappan, V. K. Aggarwal, *J. Am. Chem. Soc.* **2015**, *137*, 10100-10103. (b) J. L. Stymiest, V. Bagutski, R. M. French, V. K. Aggarwal, *Nature* **2008**, *456*, 778-782. (c) A. P. Pulis, D. J. Blair, E. Torres, V. K. Aggarwal, *J. Am. Chem. Soc.* **2013**, *135*, 16054-16057. (d) A. Bonet, M. Odachowski, D. Leonori, S. Essafi, V. K. Aggarwal, *Nat. Chem.* **2014**, *6*, 584-589. (e) D. Noh, S. K. Yoon, J. Won, J. Y. Lee, J. Yun, *Chem. Asian J.* **2011**, *6*, 1967-1969. (f) K. Toribatake, H. Nishiyama, *Angew. Chem. Int. Ed.* **2013**, *52*, 11011-11015. (g) J. R. Coombs, F. Haeffner, L. T. Kliman, J. P. Morken, *J. Am. Chem. Soc.* **2013**, *135*, 11222-11231. (h) H. Ito, K. Kubota, *Org. Lett.* **2012**, *14*, 890-893. (i) M. P. Webster, B. M. Patridge, V. K. Aggarwal, *Org. Synth.* **2011**, *88*, 247-259. (j) R. Larouche-Gauthier, T. G. Elford, V. K. Aggarwal, *J. Am. Chem. Soc.* **2011**, *133*, 16794-16797. (k) R. Rasappan, V. K. Aggarwal, *Nat. Chem.* **2014**, *6*, 810-814. (l) Y. Li, S. Chakrabarty, A. Studer, *Angew. Chem. Int. Ed.* **2015**, *54*, 3587-3591.

2. Reaction Optimization

To a stirred solution of **1a** (0.15 mmol, 1.0 equiv.) and **2a** (1.0 M in THF, 1.3-2.0 equiv.) in solvent (1.0 mL) under N_2 at **T** °C (see table below) was added freshly prepared LDA (0.86 M in THF or Et_2O, 1.3-2.0 equiv.) dropwise (approximately rate of addition = 10 μL every 10 s). The solution was then stirred at **T** °C for 1 h[3] before the addition of a solution of I-X (1.5-2.2 equiv.) in methanol (2.0 mL) dropwise over 5 min. The reaction was stirred for 5 min at **T** °C before warming to r.t. and stirred for a further 1 h. After this time a sample was removed and analysed by GCMS.

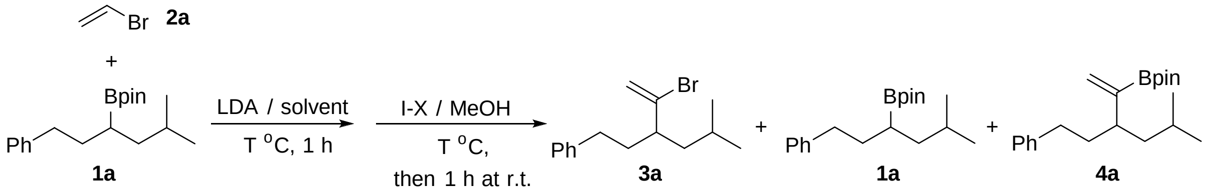

Entry	Vinyl bromide (equiv.)	LDA equiv. (solvent)[a]	T (°C)	Solvent	I-X (equiv.)	Product Ratio (3a : 1a : 4a)[b]
1	**1.3**	1.3 (Et_2O)	–78	THF	I_2 (1.5)	49 : 51 : 0
2	**1.3**	1.3 (Et_2O)	–78	THF	NIS (1.5)	55 : 45 : 0
3	**1.3**	1.3 (Et_2O)	–78	Et_2O	I_2 (1.5)	81 : 14 : 5
4	**1.3**	1.3 (Et_2O)	–78	TBME	I_2 (1.5)	79 : 18 : 3
5	**1.3**	1.3 (Et_2O)	–78	Et_2O	NIS (1.5)	83 : 17 : 0
6	**1.3**	1.3 (Et_2O)	–78	Et_2O	ICl (1.5)	83 : 17 : 0
7	**1.3**	1.3 (Et_2O)	–110	Et_2O	I_2 (1.5)	52 : 48 : 0
8	**2.0**	2.0 (Et_2O)	–110	Et_2O	I_2 (2.2)	88 : 12 : 0
9	**2.0**	2.0 (Et_2O)	–78	Et_2O	I_2 (2.2)	84 : 16 : 0
10	**2.0**	2.0 (Et_2O)	–95	Et_2O	I_2 (2.2)	87 : 13 : 0
11	**2.0**	2.0 (Et_2O)	–95[c]	Et_2O	I_2 (2.2)	87 : 13 : 0
12	**2.0**	2.0 (THF)	–95	THF	I_2 (2.2)	91 : 9 : 0
13	**2.0**	**2.0 (THF)**	**–95**	**Et_2O**	**I_2 (2.2)**	**96 : 4 : 0**
14	**2.0[d]**	2.0 (Et_2O)	–95	Et_2O	I_2 (2.2)	0 : 100 : 0

[a] solvent used to prepare the LDA solution. [b] determined by GCMS. [c] the reaction was stirred at –95 °C for 2 h before the addition of I_2. [d] vinyl bromide used as a 1.0 M solution in Et_2O.[4]

[3] The generation of the ate complex between the boronic ester and lithiated vinyl bromide cannot be followed by ^{11}B NMR spectroscopy due to the instability of the ate complex at higher temperatures. However, after the addition of methanol (1.0 mL) to this solution, the ate complex can be observed by ^{11}B NMR after warming to room temperature.

[4] It is important to note that this reaction cannot be performed without THF, as the reaction fails to give any product when Et_2O is used as the only solvent (entry 14). This demonstrates the dramatic influence of THF on the reactivity of LDA, see: D. B. Collum, A, J. McNeil, A. Ramirez, *Angew. Chem. Int. Ed.* **2007**, *46*, 3002-3017.

3. Alkynylation General Procedures

General Procedure A:

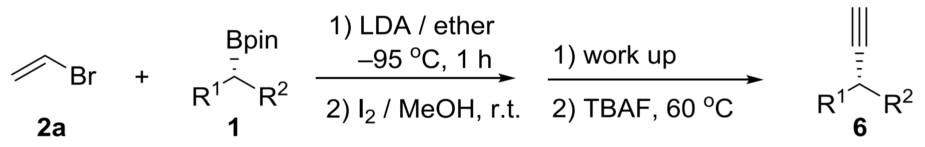

To a stirred solution of **2a** (1.0 M in THF, 0.60 ml, 0.60 mmol) and **1** (0.30 mmol) in diethyl ether (2.0 mL) under N_2 at –95 °C was added freshly prepared LDA (0.86 M in THF, 0.70 mL, 0.60 mmol) dropwise at a rate of approximately 10 µL every 10 s. The resulting solution was stirred for 1 h at –95 °C before the addition of a solution of I_2 (168 mg, 0.66 mmol) in MeOH (2.0 mL) dropwise over 10 min. The reaction was stirred for 5 min at –95 °C before warming to r.t. and stirring for 1 h. The reaction was quenched by the addition of 20% $Na_2S_2O_3$ (10 mL) and the aqueous phase extracted with Et_2O (2 × 15 mL). The combined organic phases were washed with water (15 mL), dried over $MgSO_4$, filtered and concentrated *in vacuo*. The crude product was then re-dissolved in DMF (4.0 mL) and TBAF·$3H_2O$ (473 mg, 1.50 mmol) was added. The resulting solution was heated to 60 °C for 1 h before being cooled to r.t. and diluted with H_2O (10 mL). The aqueous phase was extracted with Et_2O (2 × 15 mL) and the combined organic phases dried over $MgSO_4$, filtered and concentrated *in vacuo*. The crude product purified by flash column chromatography on silica gel.

Representative TLC example shown below for ***General Procedure A***:

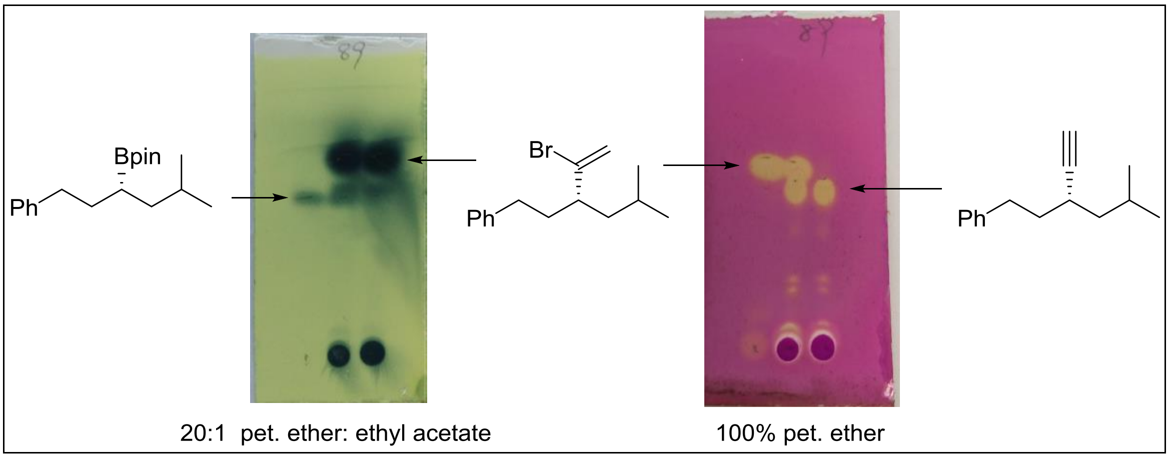

General Procedure B:

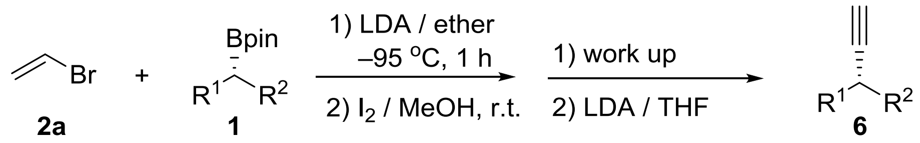

To a stirred solution of **2a** (1.0 M in THF, 0.60 ml, 0.60 mmol) and **1** (0.30 mmol) in diethyl ether (2.0 mL) under N_2 at –95 °C was added freshly prepared LDA (0.86 M in THF, 0.70 mL, 0.60 mmol) dropwise at a rate of approximately 10 µL every 10 s. The resulting solution was stirred for 1 h at –95 °C before the addition of a solution of I_2 (168 mg, 0.66 mmol) in MeOH (2.0 mL) dropwise over 10 min. The reaction was stirred for 5 min at –95 °C before warming to r.t. and stirring for 1 h. The reaction was quenched by the addition of 20% $Na_2S_2O_3$ (10 mL) and the aqueous phase extracted with Et_2O (2 × 15 mL). The combined organic phases were washed with water (15 mL), dried over $MgSO_4$, filtered and concentrated *in vacuo*. The crude product was then re-dissolved in THF (2.0 mL) and cooled to –78 °C before the dropwise addition of LDA (0.86 M, 0.80 mmol, 0.93 mL in THF). The reaction was removed from the cold bath and stirred at r.t. for 1 h before quenching with saturated $NH_4Cl_{(aq)}$ (10 mL). The aqueous phase was extracted with Et_2O (2 × 15 mL) and the combined organic phases dried over $MgSO_4$, filtered and concentrated *in vacuo*. The crude product was purified by flash column chromatography on silica gel.

General Procedure C:

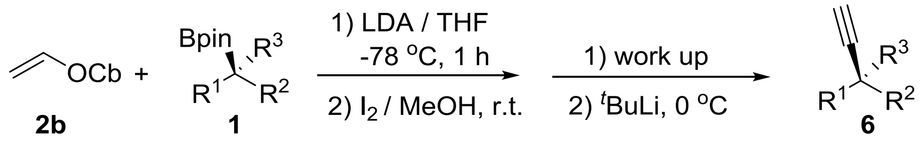

To a stirred solution of **2b** (68 mg, 0.40 mmol)[5] and **1** (0.30 mmol) in THF (2.0 mL) under N_2 at –78 °C was added freshly prepared LDA (0.86 M in THF, 0.47 mL, 0.40 mmol) dropwise at a rate of approximately 10 µL every 10 s. The resulting solution was stirred for 1 h at –78 °C before the addition of a solution of I_2 (102 mg, 0.40 mmol) in MeOH (2.0 ml) dropwise over 5 min. The reaction was stirred for 5 min at –78 °C before warming to r.t. and stirred for 1 h. The reaction was quenched by the addition of 20% $Na_2S_2O_3$ (10 mL) and the aqueous phase extracted with Et_2O (2 × 15 mL). The combined organic phases were washed with water

[5] Prepared according to: (a) A. M. Fournier, J. Clayden, *Org. Lett.* **2012**, *14*, 142-145. (b) N. J. Webb, S. P. Marsden, S. A. Raw, *Org. Lett.* **2014**, *16*, 4718-4721.

(15 mL), dried over $MgSO_4$, filtered through a short pad of silica gel (2 cm) and concentrated *in vacuo*. The crude product was then re-dissolved in Et_2O (3.0 ml) cooled to –78 °C and ^{t}BuLi (1.7 M, 0.44 ml, 0.75 mmol) was added. The reaction was then transferred to a 0 °C bath and stirred for 30 min before the addition of saturated $NH_4Cl_{(aq)}$ (10 mL). The aqueous phase was extracted with Et_2O (2 × 15 mL) and the combined organic phases dried over $MgSO_4$, filtered and concentrated *in vacuo*. The crude product purified by flash column chromatography on silica gel.

Representative TLC example shown below for ***General Procedure C***:

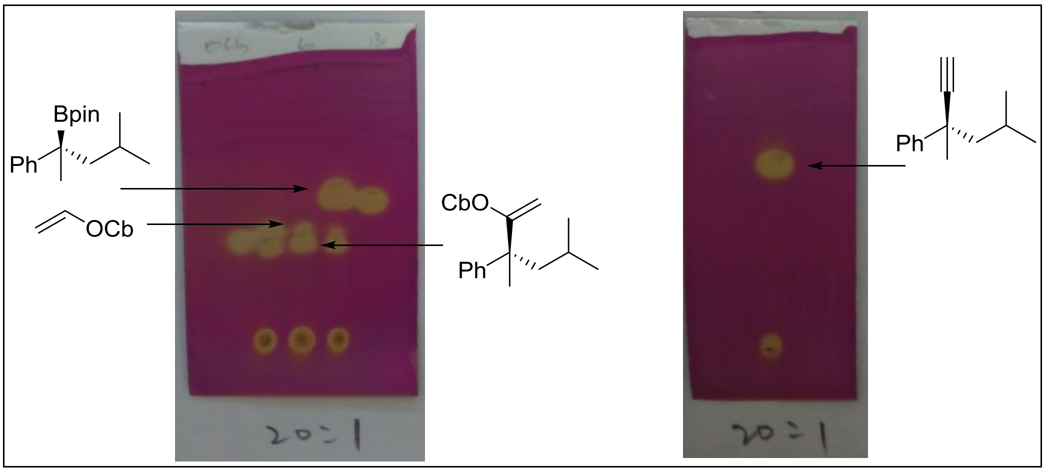

4. Product Characterization (From Vinyl Bromide)

(*S*)-(3-Ethynyl-5-methylhexyl)benzene (6a)

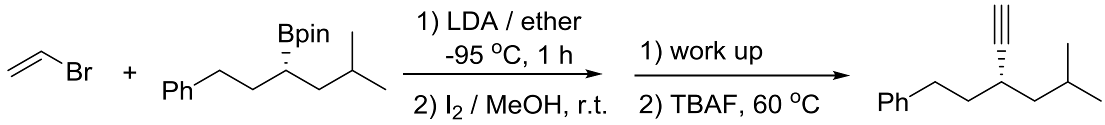

The starting boronic ester **1a** (er 98:2, 151 mg, 0.500 mmol) was reacted with vinyl bromide according to General Procedure A. The crude product was purified by column chromatography (petroleum ether) to afford the title compound as a colourless oil in 81% yield (81 mg) and 98:2 er (100% es).

[α]$_D$^{23} = –27 (*c* 1.0, $CHCl_3$); **R$_f$** (petroleum ether): 0.5; **IR** (film) ν_{max}/cm^{-1}: 3320, 2952, 1442, 7023, 623; **^{1}H NMR** ($CDCl_3$, 400 MHz) δ (ppm): 7.30 – 7.25 (m, 2H), 7.22 – 7.15 (m, 3H), 2.90 – 2.82 (m, 1H), 2.76 – 2.67 (m, 1H), 2.45 – 2.36 (m, 1H), 2.10 (d, *J* = 2.4 Hz, 1H), 1.90 – 1.81 (m, 1H), 1.77 – 1.70 (m, 2H), 1.50 – 1.42 (m, 1H), 1.23 – 1.18 (m, 1H), 0.90 (d, *J* = 6.7 Hz, 3H), 0.86 (d, *J* = 6.6 Hz, 3H); **^{13}C NMR** ($CDCl_3$, 100 MHz) δ (ppm): 142.1, 128.5, 128.4, 125.8, 87.7, 69.6, 44.2, 37.2, 33.5, 29.2, 25.9, 23.3, 21.7; **HRMS** (EI) mass calculated for $[M]^+$ ($C_{15}H_{20}$) requires *m/z* 200.1565, found *m/z* 200.1561.

The er was determined by HPLC [chiralpak IB with guard, hexane/isopropanol 100/0, 0.5 mL/min, rt, λ = 210 nm, t (major) = 10.0 min, t (minor) = 11.7 min] to be 98:2 (100% es):

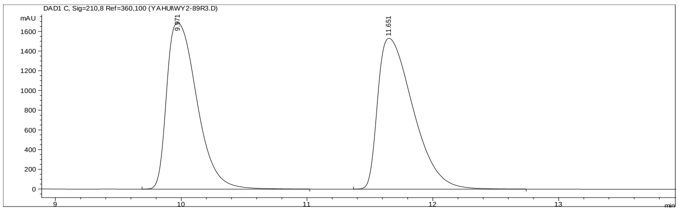

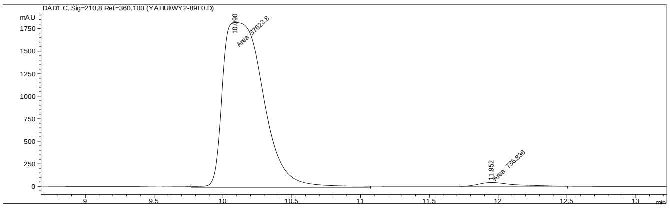

Peak #	RetTime [min]	Type	Width [min]	Area [mAU*s]	Height [mAU]	Area %
1	9.971	BB	0.2606	2.81411e4	1686.77783	48.5420
2	11.651	BB	0.3034	2.98315e4	1530.48181	51.4580

Peak #	RetTime [min]	Type	Width [min]	Area [mAU*s]	Height [mAU]	Area %
1	10.090	MM	0.3428	3.76228e4	1828.99304	98.0791
2	11.952	MM	0.2924	736.83624	42.00341	1.9209

(*S*)-(3-Cyclopropylpent-4-yn-1-yl)benzene (6b)

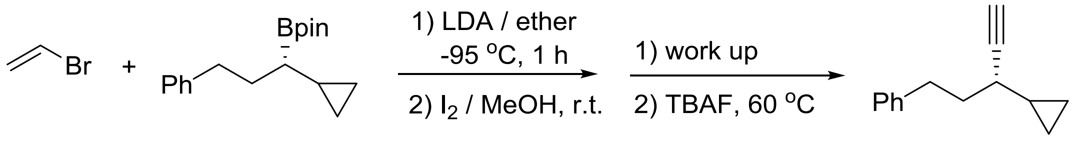

The starting boronic ester **1b** (er 98:2, 86 mg, 0.30 mmol) was reacted with vinyl bromide according to General Procedure A. The crude product was purified by column chromatography (petroleum ether) to afford the title compound as a colourless oil in 54% yield (30 mg) and 98:2 er (100% es).

[α]$_D$^{23} = –9 (*c* 1.0, $CHCl_3$); **R$_f$** (petroleum ether): 0.6; **IR** (film) ν_{max}/cm^{-1}: 3306, 3003, 2924, 2857, 1497, 1455, 1020, 748, 698, 632; **^{1}H NMR** ($CDCl_3$, 300 MHz) δ (ppm): 7.34 – 7.28 (m, 2H), 7.26 – 7.16 (m, 3H), 2.97 – 2.86 (m, 1H), 2.82 – 2.71 (m, 1H), 2.15 – 2.08 (m, 2H), 1.98 – 1.88 (m, 2H), 0.95 – 0.86 (m, 1H), 0.56 – 0.22 (m, 4H).; **^{13}C NMR** ($CDCl_3$, 75 MHz) δ (ppm): 142.1, 128.5, 128.4, 125.9, 85.6, 69.9, 37.1, 34.9, 33.5, 14.9, 3.8, 2.7; **HRMS** (EI) mass calculated for $[M]^+$ ($C_{14}H_{16}$) requires *m/z* 184.1252, found *m/z* 252.1248.

The er was determined by HPLC [chiralpak IB with guard, hexane/isopropanol 100/0, 0.5 mL/min, rt, λ = 210 nm, t (major) = 12.0 min, t (minor) = 13.0 min] to be 98:2 (100% es):

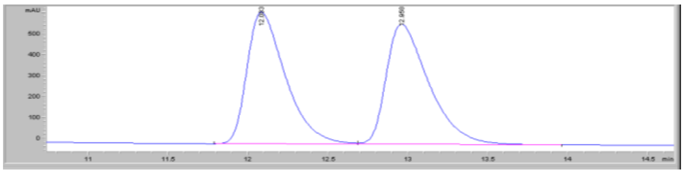

Peak #	RetTime [min]	Type	Width [min]	Area [mAU*s]	Height [mAU]	Area %
1	12.083	PV	0.2535	1.05702e4	630.65308	49.7796
2	12.958	VB	0.2804	1.06639e4	574.98547	50.2204

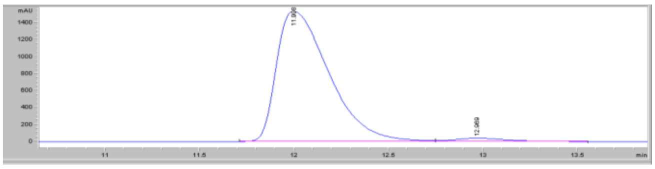

Peak #	RetTime [min]	Type	Width [min]	Area [mAU*s]	Height [mAU]	Area %
1	11.998	BV	0.2983	2.95216e4	1535.43127	97.6089
2	12.969	VB	0.2789	723.19525	38.55061	2.3911

(*R*)-1-(3-Ethynyl-7-methyloct-6-en-1-yl)-4-methoxybenzene (6c)

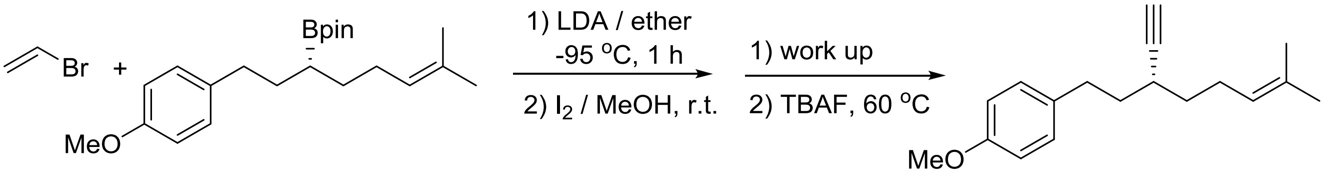

The starting boronic ester **1c** (er 94:6, 108 mg, 0.300 mmol) was reacted with vinyl bromide according to General Procedure A. The crude product was purified by column chromatography (petroleum ether/ethyl acetate = 50:1) to afford the title compound as a colourless oil in 76% yield (58 mg) and 94:6 er (100% es).

[α]$_D$^{23} = –19 (*c* 1.0, $CHCl_3$); **R$_f$** (petroleum ether/EA = 20:1): 0.8; **IR** (film) ν_{max}/cm^{-1}: 3299, 2924, 2856, 1511, 1244, 1176, 1037, 827, 630; **^{1}H NMR** ($CDCl_3$, 400 MHz) δ (ppm): 7.10 (d, *J* = 8.8 Hz, 2H), 6.82 (d, *J* = 8.8 Hz, 2H), 5.08 (d, *J* = 7.2 Hz, 1H), 3.78 (s, 3H), 2.85 – 2.73 (m, 1H), 2.69 – 2.62 (m, 1H), 2.36 – 2.30 (m, 1H), 2.20 – 2.05 (m, 3H), 1.75 – 1.67 (m, 5H), 1.60 (s, 3H), 1.53 – 1.43 (m, 2H). **^{13}C NMR** ($CDCl_3$, 100 MHz)

δ (ppm): δ 157.7, 134.0, 132.1, 129.3, 123.7, 113.7, 87.6, 69.7, 55.2, 36.9, 35.0, 32.5, 30.5, 25.7, 25.7, 17.6; **HRMS** (EI) mass calculated for $[M]^+$ ($C_{18}H_{24}O$) requires *m/z* 256.1827, found *m/z* 256.1820.

The er was determined by HPLC [chiralpak IB with guard, hexane/isopropanol 100/0, 0.5 mL/min, rt, λ = 210 nm, t (major) = 24.7 min, t (minor) = 29.9 min] to be 94:6 (100% es):

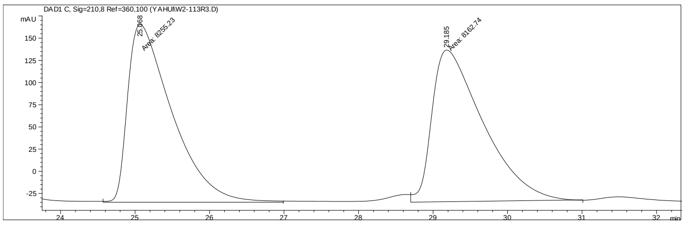

Peak #	RetTime [min]	Type	Width [min]	Area [mAU*s]	Height [mAU]	Area %
1	25.068	MM	0.6873	8255.22949	200.19002	50.2817
2	29.185	MM	0.7963	8162.73828	170.85403	49.7183

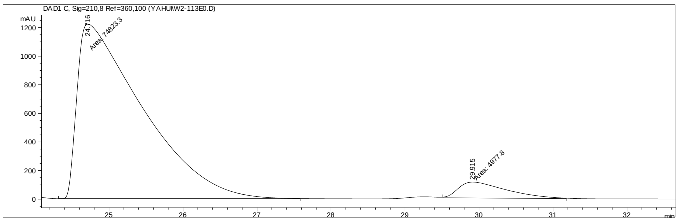

Peak #	RetTime [min]	Type	Width [min]	Area [mAU*s]	Height [mAU]	Area %
1	24.716	MM	1.0219	7.48233e4	1220.31055	93.7622
2	29.915	MM	0.7480	4977.79541	110.91131	6.2378

(*R*)-(7-Azido-3-ethynylheptyl)benzene (6d)

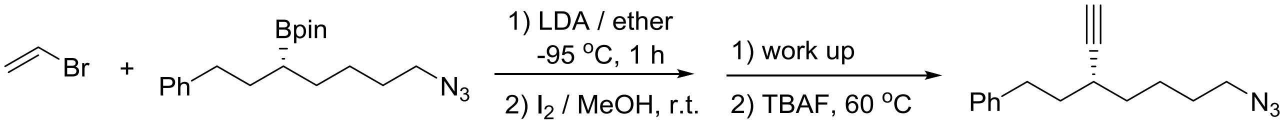

The starting boronic ester **1d** (er 99:1, 172 mg, 0.500 mmol) was reacted with vinyl bromide according to General Procedure A. The crude product was purified by column chromatography (petroleum ether/ether = 100:1) to afford the title compound as a colourless oil in 73% yield (88 mg) and 99:1 er (100% es).

$[\alpha]_D^{23}$ = –30 (*c* 1.0, $CHCl_3$); **R_f** (petroleum ether/EA = 20:1): 0.2; **IR** (film) ν_{max}/cm^{-1}: 3301, 2938, 2861, 2092, 1255, 699, 685; **^{1}H NMR** ($CDCl_3$, 400 MHz) δ (ppm): 7.28 (t, *J* = 8.3 Hz, 2H), 7.23 – 7.15 (m, 3H), 3.26 (t, *J* = 6.6 Hz, 2H), 2.90 – 2.82 (m, 1H), 2.75 – 2.66 (m, 1H), 2.38 – 2.32 (m, 1H), 2.14 (d, *J* = 2.4 Hz, 1H), 1.80 – 1.73 (m, 2H), 1.68 – 1.44 (m, 6H); **^{13}C NMR** ($CDCl_3$, 100 MHz) δ (ppm): 141.8, 128.4, 128.3, 125.8, 87.0, 70.1, 51.3, 36.7, 34.4, 33.4, 30.9, 28.6, 24.4; **HRMS** (CI) mass calculated for $[M–N_2+H]^+$ ($C_{15}H_{20}N$) requires *m/z* 214.1596, found *m/z* 214.1594.

The er was determined by HPLC [chiralpak IA with guard, hexane/isopropanol 100/0, 0.2 mL/min, rt, λ = 210 nm, t (major) = 67.5 min, t (minor) = 73.1 min] to be 99:1 (100% es):

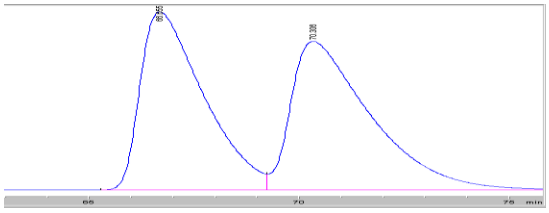

#	Time	Area	Height	Width	Area%	Symmetry
1	66.655	18950.1	180.3	1.5423	48.797	0.419
2	70.306	19884.4	150.4	1.8805	51.203	0.365

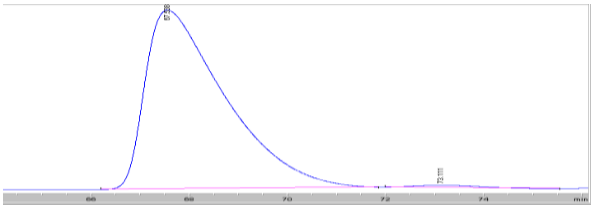

#	Time	Area	Height	Width	Area%	Symmetry
1	67.538	27008.9	239.2	1.6332	99.000	0.363
2	73.111	272.9	3.1	1.0345	1.000	0.694

(*R*)-1-Methoxy-4-(3-methylpent-4-yn-1-yl)benzene (6e)

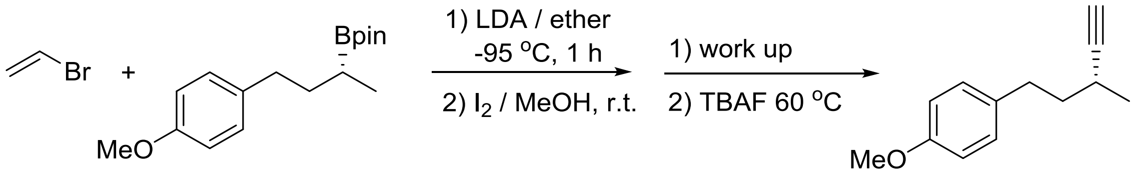

The starting boronic ester **1e** (er 96:4, 87 mg, 0.30 mmol) was reacted with vinyl bromide according to General Procedure A. The crude product was purified by column chromatography (petroleum ether/ethyl acetate = 50:1) to afford the title compound as a colourless oil in 74% yield (42 mg) and 96:4 er (100% es).

[α]D^{23} = –50 (*c* 1.0, $CHCl_3$); **R$_f$** (petroleum ether/ethyl acetate = 20:1): 0.3; **IR** (film) ν_{max}/cm^{-1}: 3294, 2931, 2110, 1611, 1511, 1242, 1176, 1036, 824, 630; **^{1}H NMR** ($CDCl_3$, 300 MHz) δ (ppm): 7.12 (d, *J* = 8.3 Hz, 2H), 6.83 (d, *J* = 8.3 Hz, 3H), 3.78 (d, *J* = 0.9 Hz, 3H), 2.84 – 2.57 (m, 2H), 2.49 – 2.34 (m, 1H), 2.14 – 2.05 (m, 1H), 1.81 – 1.61 (m, 2H), 1.20 (dd, *J* = 6.9, 1.0 Hz, 3H); **^{13}C NMR** ($CDCl_3$, 75 MHz) δ (ppm): 157.8, 134.0, 129.4, 113.8, 88.8, 68.7, 55.3, 38.7, 32.6, 25.1, 21.0; **HRMS** (EI) mass calculated for $[M]^+$ ($C_{13}H_{16}O$) requires *m/z* 188.1201, found *m/z* 188.1200.

The er was determined by HPLC [chiralpak IB with guard, hexane/isopropanol 100/0, 0.5 mL/min, rt, λ = 210 nm, t (major) = 24.5 min, t (minor) = 28.2 min] to be 96:4 (100% es):

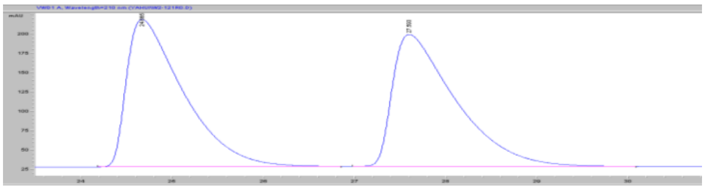

#	Time	Area	Height	Width	Area%	Symmetry
1	24.665	8227.5	190.4	0.6438	50.093	0.347
2	27.593	8197.1	170.6	0.7107	49.907	0.335

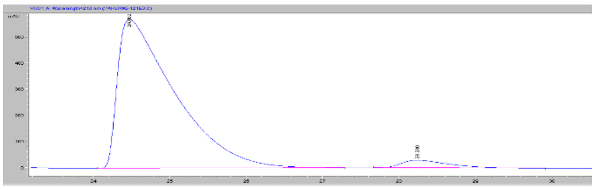

#	Time	Area	Height	Width	Area%	Symmetry
1	24.462	29629.3	569.4	0.7498	95.848	0.238
2	28.23	1283.4	29.8	0.6496	4.152	0.478

(*R*)-*tert*-Butyldimethyl((7-phenethylnon-8-yn-1-yl)oxy)silane (6f)

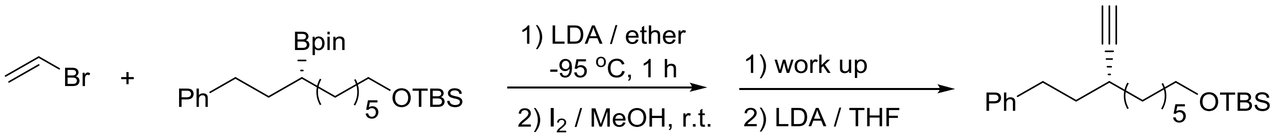

The starting boronic ester **1f** (er 96:4, 138 mg, 0.300 mmol) was reacted with vinyl bromide according to General Procedure B. The crude product was purified by column chromatography (petroleum ether/ethyl acetate = 100:1) to afford the title compound as a colourless oil in 72% yield (77 mg) and 94:6 er (96% es).

$[\alpha]_D^{23}$ = –15 (*c* 1.0, $CHCl_3$); **R_f** (petroleum ether/ethyl acetate = 100:1): 0.2; **IR** (film) ν_{max}/cm^{-1}: 2928, 2856, 1255, 1094, 833, 697, 627; **^{1}H NMR** ($CDCl_3$, 300 MHz) δ (ppm): 7.34 – 7.14 (m, 5H), 3.60 (td, *J* = 6.5, 0.6 Hz, 2H), 2.87 (dt, *J* = 14.6, 7.5 Hz, 1H), 2.71 (dt, *J* = 13.7, 8.2 Hz, 1H), 2.43 – 2.26 (m, 1H), 2.13 (d, *J* = 2.4 Hz, 1H), 1.76 (dd, *J* = 8.2, 7.1 Hz, 2H), 1.58 – 1.28 (m, 10H), 0.91 (s, 9H), 0.06 (s, 6H).**^{13}C NMR** ($CDCl_3$, 75 MHz) δ (ppm): 142.1, 128.5, 128.4, 125.9, 87.7, 69.7, 63.3, 36.8, 35.0, 33.6, 32.9, 31.1, 29.3, 27.2, 26.1, 25.8, 18.4, -5.1; **HRMS** (CI) mass calculated for $[M+H]^+$ ($C_{23}H_{39}OSi$) requires *m/z* 359.2770, found *m/z* 359.2774.

The er was determined by HPLC [chiralpak IB with guard, hexane/isopropanol 100/0, 0.5 mL/min, rt, λ = 210 nm, t (major) = 12.8 min, t (minor) = 14.3 min] to be 94:6 (96% es):

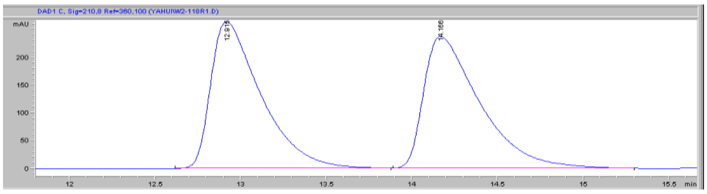

Peak #	RetTime [min]	Type	Width [min]	Area [mAU*s]	Height [mAU]	Area %
1	12.915	PB	0.3007	5407.94141	264.29913	49.8607
2	14.166	BB	0.3410	5438.16650	237.57841	50.1393

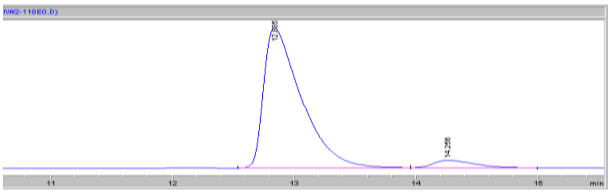

Peak #	RetTime [min]	Type	Width [min]	Area [mAU*s]	Height [mAU]	Area %
1	12.835	BB	0.3208	1.15019e4	530.85284	94.3598
2	14.256	BB	0.3509	687.50049	29.17407	5.6402

***tert*-Butyl (*S*)-4-phenethylhex-5-ynoate (6g)**

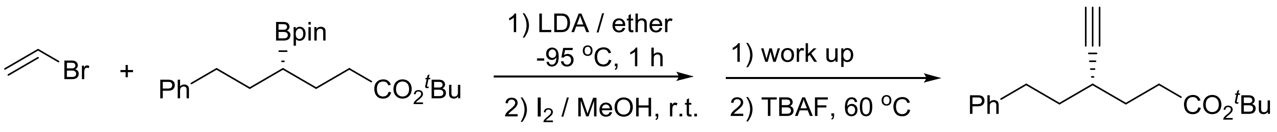

The starting boronic ester **1g** (er 97:3, 70 mg, 0.19 mmol) was reacted with vinyl bromide according to General Procedure A. The crude product was purified by column chromatography (petroleum ether/ethyl acetate = 100:1) to afford the title compound as a colourless oil in 68% yield (35 mg) and 97:3 er (100% es).

[α]$_D$^{23} = –17 (*c* 1.0, $CHCl_3$); **R$_f$** (petroleum ether/EA = 20:1): 0.7; **IR** (film) ν_{max}/cm^{-1}: 3312, 2927, 2857, 1728, 1454, 1366, 1251, 1148, 850, 744; **^{1}H NMR** ($CDCl_3$, 400 MHz) δ (ppm): 7.30 – 7.23 (m, 2H), 7.22 – 7.14 (m, 3H), 2.91 – 2.78 (m, 1H), 2.71 (dt, *J* = 13.7, 8.3 Hz, 1H), 2.50 – 2.28 (m, 3H), 2.13 (d, *J* = 2.4 Hz, 1H), 1.87 – 1.63 (m, 4H), 1.42 (s, 9H); **^{13}C NMR** ($CDCl_3$, 100 MHz) δ (ppm): δ 172.7, 141.8, 128.5, 128.4, 125.9, 86.5, 80.3, 70.6, 36.7, 33.5, 33.3, 30.5, 30.1, 28.1; **HRMS** (ESI) mass calculated for $[M+Na]^+$ ($C_{18}H_{24}NaO_2$) requires *m/z* 295.1669, found *m/z* 295.1672.

The er was determined by HPLC [chiralpak IB with guard, hexane/isopropanol 100/0, 1 mL/min, rt, λ = 210 nm, t (minor) = 27.6 min, t (major) = 29.3 min] to be 97:3 (100% es):

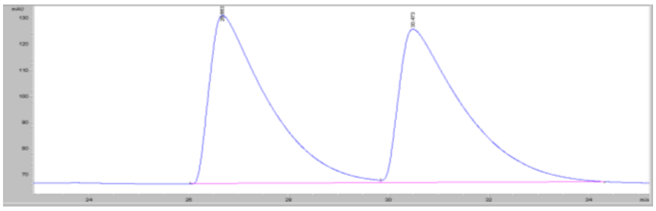

#	Time	Area	Height	Width	Area%	Symmetry
1	26.663	5230	64.6	1.1553	49.932	0.269
2	30.473	5244.2	59	1.2625	50.068	0.262

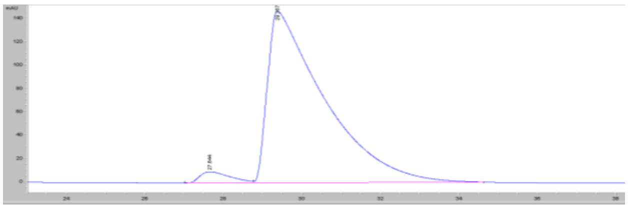

#	Time	Area	Height	Width	Area%	Symmetry
1	27.644	504	9.5	0.7896	3.300	0.467
2	29.367	14767.7	146.9	1.3605	96.700	0.199

(1*R*,2*R*,4*R*)-2-Ethynyl-1-isopropyl-4-methylcyclohexane (6h)

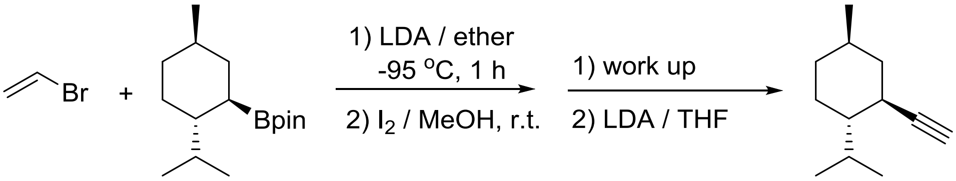

The starting boronic ester **1h** (>25:1 dr, 80 mg, 0.30 mmol) was reacted with vinyl bromide according to General Procedure B. The crude product was purified by column chromatography (petroleum ether) to afford the title compound as a colourless oil in 53% yield (26 mg) and dr >25:1 [ds 100%, determined by ^{1}H NMR].

[α]$_D$^{23} = –30 (*c* 0.37, $CHCl_3$); **R$_f$** (petroleum ether): 0.6; **^{1}H NMR** ($CDCl_3$, 400 MHz) δ (ppm): 2.28 (heptd, *J* = 7.0, 2.8 Hz, 1H), 2.17 (dddd, *J* = 11.9, 11.1, 3.6, 2.4 Hz, 1H), 2.06 (d, *J* = 2.4 Hz, 1H), 2.01 (dtd, *J* = 12.9, 3.5, 2.2 Hz, 1H), 1.76 – 1.69 (m, 1H), 1.67 – 1.60 (m, 1H), 1.41 – 1.27 (m, 0H), 1.24 – 1.07 (m, 2H), 1.02 –

0.84 (m, 2H), 0.94 (d, *J* = 7.0 Hz, 3H), 0.90 (d, *J* = 6.6 Hz, 3H), 0.79 (d, *J* = 6.9 Hz, 3H); **^{13}C NMR** ($CDCl_3$, 126 MHz) δ (ppm): 88.0, 68.8, 47.1, 42.4, 34.7, 33.2, 32.4, 28.5, 24.0, 22.2, 21.3, 15.6. Spectral data are in agreement with those reported in the literature.[6]

Triisopropyl(((1*R*,2*S*,5*R*)-2-isopropyl-5-methylcyclohexyl)ethynyl)silane (6h')

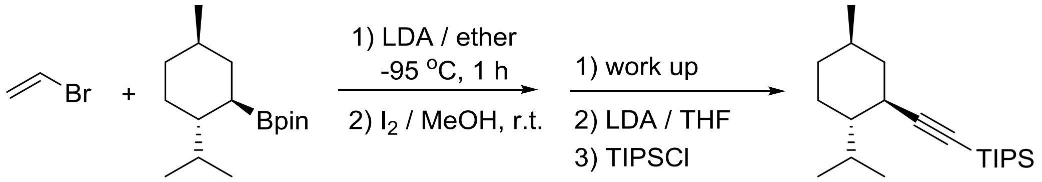

The starting boronic ester **1h** (>25:1 dr, 80 mg, 0.30 mmol) was reacted with vinyl bromide according to modified General Procedure B. After formation of the vinyl bromide intermediate, the elimination and trapping with TIPSCl was performed as follows: To a solution of crude vinyl bromide (0.30 mmol) in THF (2.0 mL) at –78 °C was added LDA (0.86 M, 0.80 mmol, 0.93 mL in THF) dropwise over 1 min. The reaction was removed from the cold bath and stirred for 1 h before the addition of triisopropylsilyl chloride (TIPSCl, 0.50 mmol, 0.11 mL). After stirring for an additional 1 h at rt saturated $NH_4Cl_{(aq)}$ (10 mL) was added and the aqueous phase was extracted with Et_2O (2 × 15 mL), the combined organic phases dried over $MgSO_4$, filtered and concentrated *in vacuo*. The crude product was purified by column chromatography (petroleum ether) to afford the title compound as a colourless oil in 65% yield (63 mg) and >25:1 dr [100% ds, determined by ^{1}H NMR].

$[\alpha]_D^{23}$ = –34 (*c* 1.0, $CHCl_3$); **R_f** (petroleum ether): 0.8; **IR** (film) ν_{max}/cm^{-1}: 2955, 2941, 2926, 2896, 2865, 2170, 1463, 1385, 1369; **^{1}H NMR** ($CDCl_3$, 400 MHz) δ (ppm): 2.36 (heptd, *J* = 6.9, 2.9 Hz, 1H), 2.19 (td, *J* = 11.5, 3.6 Hz, 1H), 2.00 (dtd, *J* = 13.0, 3.6, 2.2 Hz, 1H), 1.74 – 1.68 (m, 1H), 1.65 – 1.59 (m, 1H), 1.38 – 1.28 (m, 1H), 1.22 – 1.08 (m, 2H), 1.08 – 0.87, (m, 23H), 0.92 (d, *J* = 7.0 Hz, 3H), 0.89 (d, *J* = 6.5 Hz, 3H), 0.79 (d, *J* = 7.0 Hz, 3H); **^{13}C NMR** ($CDCl_3$, 126 MHz) δ (ppm): 112.6, 80.2, 47.5, 42.6, 34.9, 34.6, 32.5, 28.6, 24.2, 22.3, 21.2, 18.63, 18.62, 15.7, 11.3; **HRMS** (EI$^+$) calculated for $[M]^+$ ($C_{21}H_{40}Si$) requires *m/z* 320.2899, found *m/z* 320.2894.

[6] J. D. Spence, J. K. Wyatt, D. M. Bender, D. K. Moss, M. H. Nantz, *J. Org. Chem.* **1996**, *61*, 4014-4021.

tert-Butyldiphenyl(((2_R_,4_R_,6_S_)-2,4,6-trimethyloct-7-yn-1-yl)oxy)silane (6i)

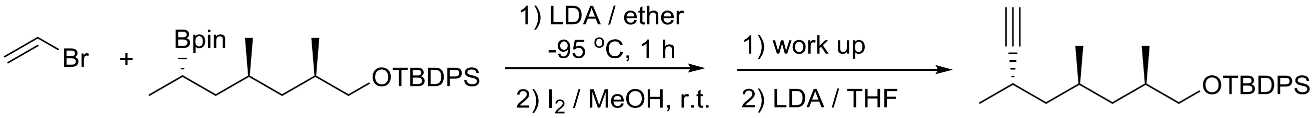

The starting boronic ester **1i** (>25:1 dr, 102 mg, 0.200 mmol) was reacted with vinyl bromide according to General Procedure B. The elimination was performed with LDA (0.80 mmol) in THF (3.0 mL) at rt for 1 h. The crude product was purified by column chromatography (petroleum ether/ethyl acetate = 100:1) to afford the title compound as a colourless oil in 70% yield (57 mg) and >25:1 dr [100% ds, determined by ^{1}H NMR].

$[\alpha]_D^{23}$ = +21 (*c* 1.0, $CHCl_3$); **R_f** (petroleum ether/ethyl acetate = 50:1): 0.3; **IR** (film) ν_{max}/cm^{-1}: 3310, 2927, 1427, 1110, 700, 614; **^{1}H NMR** ($CDCl_3$, 400 MHz) δ (ppm): 7.70 – 7.64 (m, 4H), 7.45 – 7.34 (m, 6H), 3.50 (dd, *J* = 9.8, 5.4 Hz, 1H), 3.44 (dd, *J* = 9.8, 6.3 Hz, 1H), 2.56 – 2.45 (m, 1H), 1.98 (dd, *J* = 2.4, 0.7 Hz, 1H), 1.85 – 1.70 (m, 2H), 1.52 – 1.43 (m, 1H), 1.34 (ddd, *J* = 13.7, 7.8, 6.0 Hz, 1H), 1.17 (dd, *J* = 6.9, 0.7 Hz, 3H), 1.06 (d, *J* = 0.7 Hz, 9H), 0.99 – 0.84 (m, 8H); **^{13}C NMR** ($CDCl_3$, 100 MHz) δ (ppm): 135.7, 134.2, 129.5, 127.6, 89.0, 69.1, 68.2, 44.0, 41.5, 33.1, 28.3, 27.0, 23.6, 21.8, 20.1, 19.4, 17.4; **HRMS** (CI) mass calculated for $[M+H]^+$ ($C_{27}H_{39}OSi$) requires *m/z* 407.2770, found *m/z* 407.2788.

5. Product Characterization (From Vinyl Carbamate)

(*S*)-(3,5-Dimethylhex-1-yn-3-yl)benzene (6j)

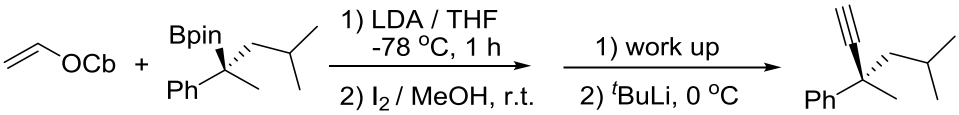

The starting boronic ester **1j** (er 98:2, 86 mg, 0.30 mmol) was reacted with vinyl diisopropylcarbamate according to General Procedure C. The crude product was purified by column chromatography (petroleum ether) to afford the title compound as a colourless oil in 89% yield (50 mg) and 98:2 er (100% es).

$[\alpha]_D^{23}$ = +10 (*c* 1.0, $CHCl_3$); **R_f** (petroleum ether/ethyl acetate = 20:1): 0.8; **IR** (film) ν_{max}/cm^{-1}: 3307, 2954, 2927, 1600, 1466, 1446, 762, 698, 626; **^{1}H NMR** ($CDCl_3$, 400 MHz) δ (ppm): 7.61 – 7.52 (m, 2H), 7.38 – 7.27 (m, 2H), 7.28 – 7.18 (m, 1H), 2.43 (d, *J* = 0.6 Hz, 1H), 1.83 – 1.64 (m, 3H), 1.60 (s, 3H), 0.94 (d, *J* = 6.5 Hz, 3H), 0.67 (d, *J* = 6.5 Hz, 3H); **^{13}C NMR** ($CDCl_3$, 100 MHz) δ (ppm): 145.2, 128.2, 126.4, 126.1, 89.8, 72.0, 52.5, 40.2, 31.8, 25.9, 24.3, 24.2; **HRMS** (EI) mass calculated for $[M]^+$ ($C_{14}H_{18}$) requires *m/z* 186.1409, found *m/z* 186.1403.

The er was determined by Chiral GC [Chiraldex β-DM, injector T = 250 °C, detector T = 300 °C. Oven conditions: T = 70 °C for 5 min then ramp (1 °C min^{-1}) until 140 °C. He carrier gas at 1.0 mL min^{-1}. t (minor) = 27.9 min, t (major) = 28.7 min] to be 98:2 (100% es):

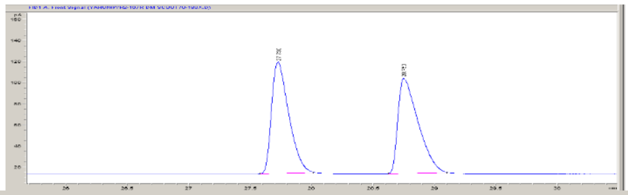

#	Time	Area	Height	Width	Area%	Symmetry
1	27.73	1030.7	105.8	0.1276	50.023	0.602
2	28.75	1029.8	90.4	0.141	49.977	0.378

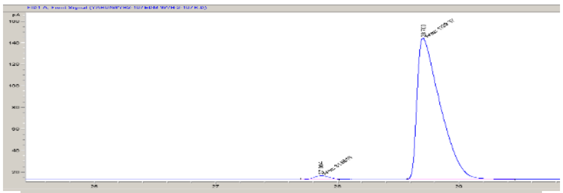

#	Time	Area	Height	Width	Symmetry
1	27.864	32	3.7	0.1455	0.835
2	28.703	1739.4	131.2	0.2209	0.329

(*S*)-(3-Methylhex-5-en-1-yn-3-yl)benzene (6k)

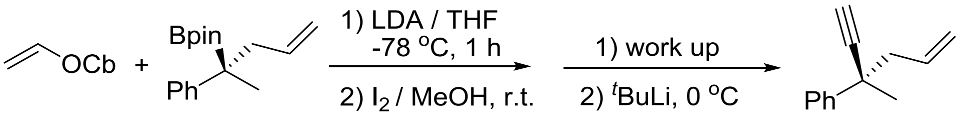

The starting boronic ester **1k** (er 99:1, 82 mg, 0.30 mmol) was reacted with vinyl diisopropylcarbamate according to General Procedure C. The crude product was purified by column chromatography (petroleum ether) to afford the title compound as a colourless oil in 72% yield (37 mg) and 99:1 er (100% es).

[α]$_D^{23}$ = +15 (*c* 1.0, $CHCl_3$); **R_f** (petroleum ether): 0.3; **IR** (film) ν_{max}/cm^{-1}: 3302, 2978, 1496, 1446, 1028, 995, 916, 763, 698, 637; **^{1}H NMR** ($CDCl_3$, 400 MHz) δ (ppm): 7.57 – 7.50 (m, 2H), 7.37 – 7.29 (m, 2H), 7.27 – 7.19 (m, 1H), 5.84 – 5.71 (m, 1H), 5.05 (td, *J* = 1.2, 0.5 Hz, 1H), 5.04 – 4.99 (m, 1H), 2.63 – 2.47 (m, 2H), 2.41 (s, 1H), 1.59 (s, 3H); **^{13}C NMR** ($CDCl_3$, 75 MHz) δ (ppm): 144.6, 134.5, 128.3, 126.6, 126.1, 117.9, 89.1, 71.7, 48.4, 40.0, 29.1; **HRMS** (EI) mass calculated for $[M]^+$ ($C_{13}H_{14}$) requires *m/z* 170.1096, found *m/z* 170.1094.

The er was determined by HPLC [chiralpak IA with guard, hexane/isopropanol 100/0, 0.3 mL/min, rt, λ = 210 nm, t (minor) = 16.4 min, t (major) = 17.0 min] to be 99:1 (100% es):

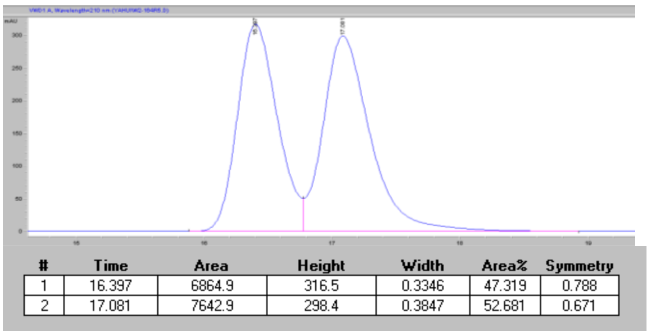

#	Time	Area	Height	Width	Area%	Symmetry
1	16.397	6864.9	316.5	0.3346	47.319	0.788
2	17.081	7642.9	298.4	0.3847	52.681	0.671

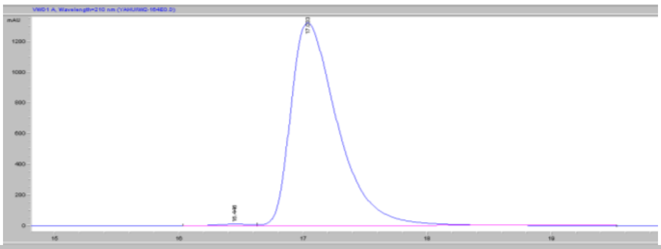

#	Time	Area	Height	Width	Area%	Symmetry
1	16.446	184.1	10	0.2899	0.527	1.137
2	17.033	34757.8	1322.8	0.4012	99.473	0.521

(*S*)-1-Chloro-4-(2-phenylbut-3-yn-2-yl)benzene (6l)

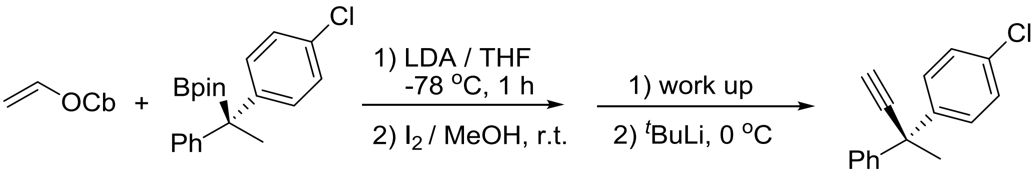

The starting boronic ester **1l** (er 98:2, 103 mg, 0.300 mmol) was reacted with vinyl diisopropylcarbamate according to General Procedure C. The crude product was purified by column chromatography (petroleum ether) to afford the title compound as a colourless oil in 87% yield (63 mg) and 98:2 er (100% es).

[α]$_D^{23}$ = –1 (*c* 1.0, $CHCl_3$); **R_f** (petroleum ether): 0.4; **IR** (film) ν_{max}/cm^{-1}: 3297, 2982, 1489, 1094, 1012, 759, 697, 637, 537; **^{1}H NMR** ($CDCl_3$, 400 MHz) δ (ppm): 7.47 – 7.42 (m, 2H), 7.39 (d, *J* = 8.3 Hz, 2H), 7.36 –

7.22 (m, 5H), 2.58 (s, 1H), 1.98 (s, 3H); **^{13}C NMR** ($CDCl_3$, 100 MHz) δ (ppm): 145.5, 144.7, 132.6, 128.5, 128.4, 128.4, 126.9, 126.9, 89.2, 72.7, 44.2, 30.5; **HRMS** (EI) mass calculated for $[M]^+$ ($C_{16}H_{13}Cl$) requires *m/z* 240.0706, found *m/z* 240.0702.

The er was determined by HPLC [chiralpak IA with guard, hexane/isopropanol 100/0, 0.3 mL/min, rt, λ = 210 nm, t (minor) = 27.5 min, t (major) = 28.7 min] to be 98:2 (100% es):

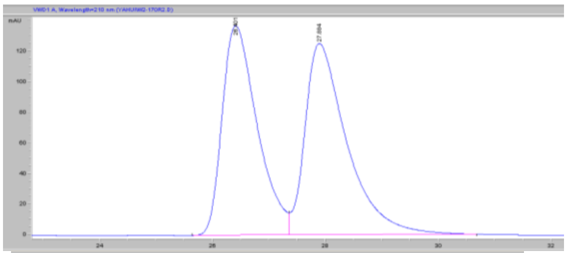

#	Time	Area	Height	Width	Area%	Symmetry
1	26.401	5920.5	137.1	0.6565	47.723	0.588
2	27.884	6485.5	125.1	0.7649	52.277	0.483

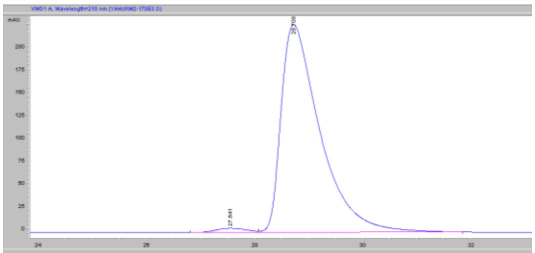

#	Time	Area	Height	Width	Area%	Symmetry
1	27.541	182.8	4.7	0.6041	1.578	0.877
2	28.708	11404.2	228.2	0.7462	98.422	0.43

(*S*)-(3-Ethyl-3-methylpent-4-yn-1-yl)benzene (6m)

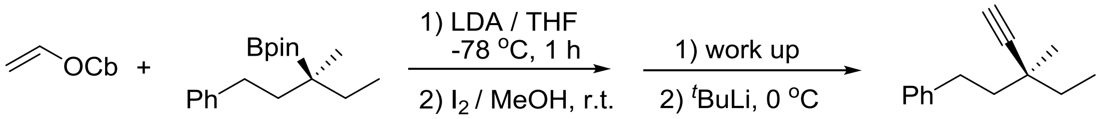

The starting boronic ester **1m** (er 99:1, 86 mg, 0.30 mmol) was reacted with vinyl diisopropylcarbamate according to General Procedure C. The crude product was purified by column chromatography (petroleum ether) to afford the title compound as a colourless oil in 84% yield (47 mg) and 99:1 er (100% es).

$[\alpha]_D^{23}$ = –17 (*c* 1.0, $CHCl_3$); **R_f** (petroleum ether): 0.3; **IR** (film) ν_{max}/cm^{-1}: 3306, 2968, 2925, 1455, 700, 627, 503; **^{1}H NMR** ($CDCl_3$, 400 MHz) δ (ppm): 7.33 – 7.15 (m, 5H), 2.86 – 2.67 (m, 2H), 2.16 (s, 1H), 1.78 (ddd, *J* = 13.4, 11.5, 5.8 Hz, 1H), 1.69 – 1.55 (m, 2H), 1.52 – 1.39 (m, 1H), 1.23 (s, 3H), 1.02 (t, *J* = 7.4 Hz, 3H); **^{13}C NMR** ($CDCl_3$, 100 MHz) δ (ppm): 142.7, 128.3, 128.3, 125.6, 90.3, 69.2, 43.1, 35.3, 34.1, 31.4, 25.7, 9.0; **HRMS** (EI) mass calculated for $[M]^+$ ($C_{14}H_{18}$) requires *m/z* 186.1409, found *m/z* 186.1407.

The er was determined by chiral GC [Chiraldex β-DM, injector T = 250 °C, detector T = 300 °C. Oven conditions: T = 70 °C ramp (0.1 °C min^{-1}) until 100 °C. He carrier gas at 1 mL min^{-1}. t (major) = 97.3 min, t (minor) = 101.4 min] to be 99:1 (100% es):

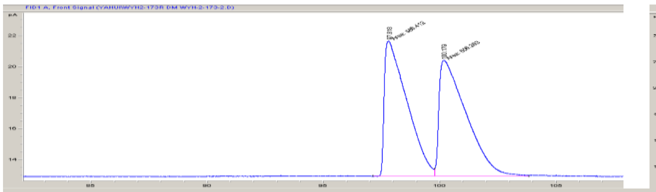

#	Time	Area	Height	Width	Area%	Symmetry
1	97.818	586.4	8.7	1.1239	49.168	0.237
2	100.179	606.3	7.4	1.3604	50.832	0.202

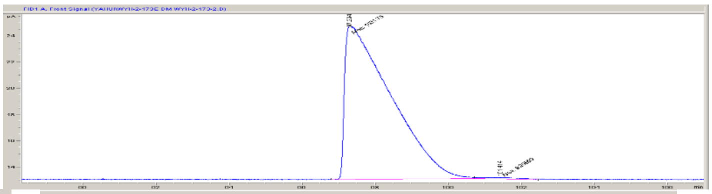

#	Time	Area	Height	Width	Area%	Symmetry
1	97.294	1031.8	11.8	1.4615	99.115	0.124
2	101.414	9.2	1.3E-1	1.2022	0.885	0.687

(*S*)-(3-Ethynylhex-5-yn-1-yl)benzene (6n)

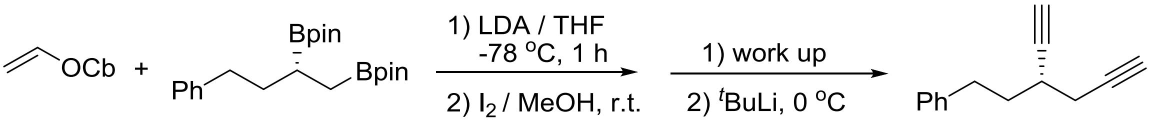

The starting boronic ester **1n** (er 97:3, 116 mg, 0.300 mmol) was reacted with vinyl diisopropylcarbamate (205 mg, 1.20 mmol), LDA (0.86 M, 1.40 mL, 1.20 mmol), I_2 (305 mg, 1.20 mmol, in 2.0 mL methanol) and ^{t}BuLi (1.7 M, 1.5 mmol, 0.88 mL) according to General Procedure C. The crude product was purified by column chromatography (petroleum ether) to afford the title compound as a colourless oil in 75% yield (41 mg) and 96:4 er (98% es).

[α]$_D^{23}$ = +56 (*c* 1.0, $CHCl_3$); **R$_f$** (petroleum ether/ethyl acetate = 3:1): 0.9; **IR** (film) ν_{max}/cm^{-1}: 3294, 2925, 2120, 1603, 1496, 1454, 699, 638; **^{1}H NMR** ($CDCl_3$, 400 MHz) δ (ppm): 7.33 – 7.25 (m, 2H), 7.24 – 7.16 (m, 3H), 2.88 (ddd, *J* = 14.3, 9.3, 5.3 Hz, 1H), 2.73 (ddd, *J* = 13.7, 9.4, 7.2 Hz, 1H), 2.60 – 2.53 (m, 1H), 2.51 – 2.34 (m, 2H), 2.21 (d, *J* = 2.4 Hz, 1H), 2.04 (t, *J* = 2.6 Hz, 1H), 1.99 – 1.79 (m, 2H); **^{13}C NMR** ($CDCl_3$, 100 MHz) δ (ppm): 141.5, 128.5, 128.5, 126.0, 85.6, 81.4, 70.6, 70.2, 35.5, 33.2, 30.3, 24.7; **HRMS** (EI) mass calculated for $[M–H]^+$ ($C_{14}H_{13}$) requires *m/z* 181.1017, found *m/z* 181.1017.

The er was determined by HPLC [chiralpak IB with guard, hexane/isopropanol 100/0, 0.5 mL/min, rt, λ = 210 nm, t (minor) = 18.3 min, t (major) = 19.5 min] to be 96:4 (98% es):

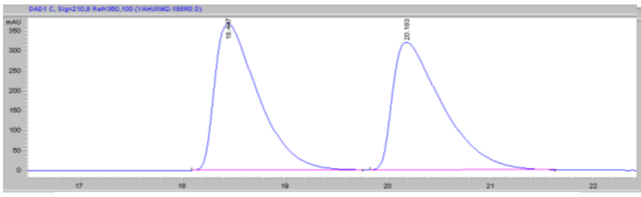

Peak #	RetTime [min]	Type	Width [min]	Area [mAU*s]	Height [mAU]	Area %
1	18.447	BB	0.4448	1.08401e4	368.92822	50.0205
2	20.183	BB	0.4960	1.08312e4	320.71317	49.9795

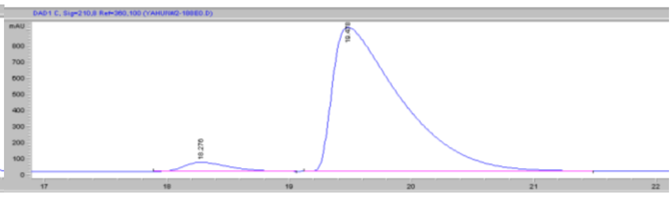

Peak #	RetTime [min]	Type	Width [min]	Area [mAU*s]	Height [mAU]	Area %
1	18.276	BB	0.3788	1398.30176	55.71978	3.8852
2	19.478	BB	0.5474	3.45922e4	894.02936	96.1148

(*S*)-(3-Cyclopropylpent-4-yn-1-yl)benzene (6b)

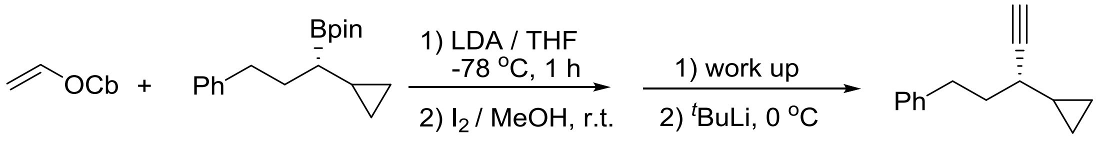

The starting boronic ester **1b** (er 98:2, 86 mg, 0.30 mmol) was reacted with vinyl diisopropylcarbamate according to General Procedure C. The crude product was purified by column chromatography (petroleum ether) to afford the title compound as a colourless oil in 77% yield (43 mg) and 98:2 er (100% es). The NMR data and HPLC analysis match with those reported for **6b** in Chapter 4 of this Supporting Information.

***tert*-Butyl(((3*S*,5*R*,6*S*,8*S*,9*S*,10*R*,13*R*,14*S*,17*R*)-6-ethynyl-10,13-dimethyl-17-((*R*)-6-methylheptan-2-yl)hexadecahydro-1*H*-cyclopenta[a]phenanthren-3-yl)oxy)dimethylsilane (6o)**

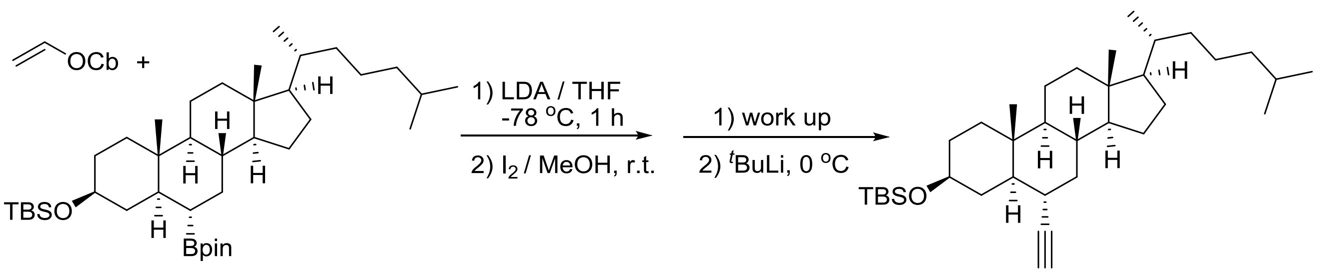

The starting boronic ester **1o** (>25:1 dr, 189 mg, 0.300 mmol) was reacted with vinyl diisopropylcarbamate according to General Procedure C. The crude product was purified by column chromatography (petroleum ether/ethyl acetate = 100:1) to afford the title compound as a white solid in 94% yield (149 mg) and >25:1 dr [100% ds, determined by ^{1}H NMR]. The crystal for x-ray analysis was obtained by slow evaporation of its pentane solution at room temperature.

M.p. = 102.1 – 104.7 °C; **$[\alpha]_D^{23}$** = +33 (*c* 1.0, $CHCl_3$); **R_f** (petroleum ether/ethyl acetate = 20:1): 0.8; **IR** (film) ν_{max}/cm^{-1}: 3311, 2931, 2853, 1470, 1250, 1099, 835, 774, 628; **^{1}H NMR** ($CDCl_3$, 400 MHz) δ (ppm): 3.60 – 3.48 (m, 1H), 2.24 – 2.13 (m, 2H), 2.05 (d, *J* = 2.4 Hz, 1H), 1.95 (d, *J* = 12.6 Hz, 2H), 1.87 – 1.75 (m, 1H), 1.72 – 1.62 (m, 2H), 1.59 – 0.83 (m, 40H), 0.78 (s, 3H), 0.68 – 0.60 (m, 4H), 0.05 (s, 6H); **^{13}C NMR** ($CDCl_3$, 75 MHz) δ (ppm): 88.0, 72.2, 69.2, 56.2, 56.0, 54.0, 48.7, 42.6, 39.9, 39.5, 39.2, 37.1, 36.2, 35.8, 35.6, 35.3, 34.8, 31.8, 30.5, 28.3, 28.0, 26.0, 24.1, 23.9, 22.9, 22.6, 21.2, 18.7, 18.3, 12.8, 12.1, -4.4, -4.5; **HRMS** (ESI) mass calculated for $[M+H]^+$ ($C_{35}H_{63}OSi$) requires *m/z* 527.4643, found *m/z* 527.4644.

(*S*)-1-(But-3-yn-2-yl)-4-methoxybenzene (6q)

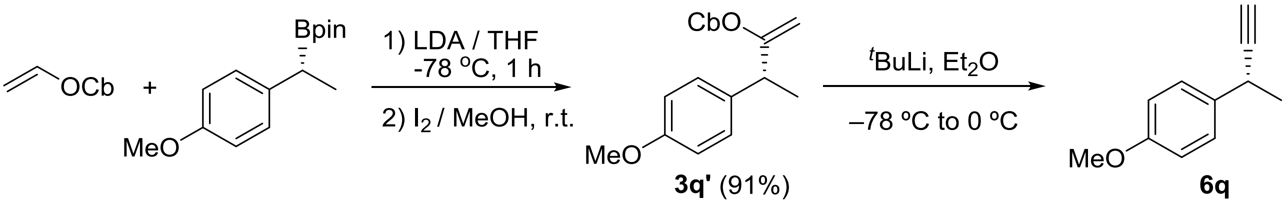

To a stirred solution of starting boronic ester **1q** (er 99:1, 524 mg, 2.00 mmol) and vinyl diisopropylcarbamate (457 mg, 2.67 mmol) in THF (8.0 mL) under N_2 at –78 °C was added freshly prepared LDA (0.86 M in THF, 3.10 mL, 2.67 mmol) dropwise. The resulting solution was stirred for 1 h at –78 °C before the addition of a solution of I_2 (678 mg, 2.67 mmol) in MeOH (6.0 ml) dropwise over 5 min. The reaction was stirred for 5 min at –78 °C before warming to r.t. and stirred for 1 h. The reaction was quenched by the addition of 20% $Na_2S_2O_3$ (10 mL) and the aqueous phase extracted with Et_2O (2 × 15 mL). The combined organic phases were washed with water (15 mL), dried over $MgSO_4$, filtered and concentrated *in vacuo*. The crude product purified by flash column chromatography on silica gel (petroleum ether/ethyl acetate = 15:1) to give **3q'** as a colourless oil (558 mg, yield 91%).

$[\alpha]_D^{23}$ = –47 (*c* 1.0, $CHCl_3$); **R_f** (petroleum ether/ethyl acetate = 20:1): 0.1; **IR** (film) ν_{max}/cm^{-1}: 2969, 1701, 1511, 1430, 1301, 1242, 1151, 1041, 831; **^{1}H NMR** ($CDCl_3$, 300 MHz) δ (ppm): 7.19 – 7.11 (m, 2H), 6.84 – 6.78 (m, 2H), 4.81 (dd, *J* = 1.5, 0.8 Hz, 1H), 4.79 – 4.75 (m, 1H), 4.01 – 3.53 (m, 6H), 1.39 (d, *J* = 7.1 Hz, 3H), 1.31 – 0.80 (m, 12H); **^{13}C NMR** ($CDCl_3$, 75 MHz) δ (ppm): 159.9, 158.3, 153.2, 135.5, 128.7, 113.7, 99.9, 55.3, 46.2, 46.0, 42.5, 20.9, 20.5, 19.8; **HRMS** (ESI) mass calculated for $[M+Na]^+$ ($C_{18}H_{27}NNaO_3$) requires *m/z* 328.1883, found *m/z* 328.1894.

The product **3q'** (122 mg, 0.400 mmol) was then dissolved in Et_2O (4.0 ml) cooled to –78 °C and ^{t}BuLi (1.7 M, 0.26 ml, 0.44 mmol) was added. The reaction was then transferred to a 0 °C bath and stirred for 30 min before the addition of saturated $NH_4Cl_{(aq)}$ (10 mL). The aqueous phase was extracted with Et_2O (2 × 15 mL) and the combined organic phases dried over $MgSO_4$, filtered and concentrated *in vacuo*. The crude product purified by preparative TLC on silica gel (petroleum ether/ethyl acetate = 10:1) to give **6q** as a colourless oil in 37% yield (24 mg) and 98:2 er (98% es) along with recovered **3q'** (42 mg). Yield of **6q** based on recovered starting material = 56%.

[α]$_D^{23}$ = +14 (*c* 1.0, $CHCl_3$); **R_f** (petroleum ether/ethyl acetate = 100:1): 0.2; **IR** (film) ν_{max}/cm^{-1}: 3290, 2974, 2932, 1608, 1509, 1243, 1177, 1032, 829, 636; **^{1}H NMR** ($CDCl_3$, 400 MHz) 7.34 – 7.27 (m, 2H), 6.90 – 6.83 (m, 2H), 3.79 (s, 3H), 3.72 (qd, *J* = 7.2, 2.5 Hz, 1H), 2.25 (d, *J* = 2.5 Hz, 1H), 1.49 (d, *J* = 7.1 Hz, 3H); **^{13}C NMR** ($CDCl_3$, 100 MHz) δ (ppm): 158.5, 134.9, 127.8, 114.0, 87.5, 69.9, 55.3, 30.8, 24.3. **HRMS** (EI) mass calculated for $[M]^+$ ($C_{11}H_{12}O$) requires *m/z* 160.0888, found *m/z* 160.0860.

The er was determined by chiral GC [Chiraldex β-DP, injector T = 250 °C, detector T = 300 °C. Oven conditions: T = 70 °C ramp (1 °C min^{-1}) until 180 °C. He carrier gas at 1 mL min^{-1}. t (minor) = 48.4 min, t (major) = 50.0 min] to be 98:2 (98% es).

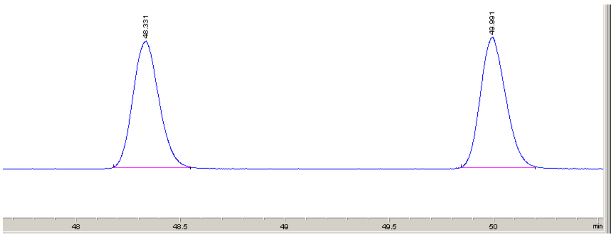

#	Time	Area	Height	Width	Area%	Symmetry
1	48.331	82	9.5	0.1285	49.889	0.899
2	49.991	82.4	9.8	0.1188	50.111	0.838

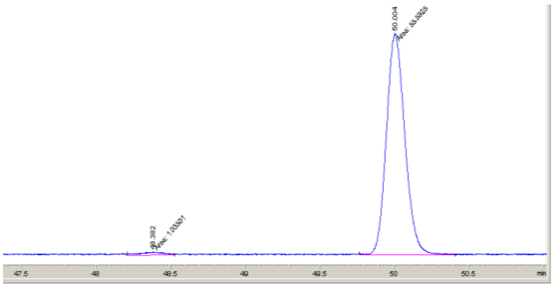

#	Time	Area	Height	Width	Area%	Symmetry
1	48.382	1	9.5E-2	0.1811	1.828	1.207
2	50.004	55.6	6.5	0.1427	98.172	0.846

6. Synthesis of Internal Alkynes from Vinyl Bromide 3a

(*S*)-(3-(1-Bromovinyl)-5-methylhexyl)benzene (3a)

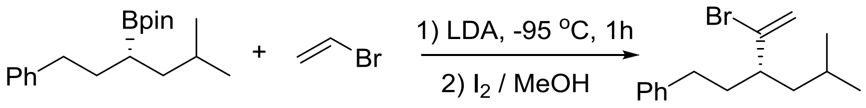

The starting boronic ester **1a** (er 98:2, 500 mg, 1.65 mmol) was reacted with vinyl bromide according to General Procedure A without elimination. The crude product was purified by column chromatography (petroleum ether) to afford the title compound as a colourless oil in 83% yield (384 mg).

$[\alpha]_D^{23}$ = +19 (*c* 1.0, $CHCl_3$); **R_f** (petroleum ether/ethyl acetate = 20:1): 0.8; **IR** (film) ν_{max}/cm^{-1}: 2927, 1623, 1454, 886, 698; **^{1}H NMR** ($CDCl_3$, 400 MHz) δ (ppm): 7.31 – 7.24 (m, 2H), 7.23 – 7.13 (m, 3H), 5.63 (d, *J* = 1.4 Hz, 1H), 5.50 (dd, *J* = 1.5, 0.5 Hz, 1H), 2.65 (ddd, *J* = 13.8, 10.2, 5.1 Hz, 1H), 2.46 (ddd, *J* = 13.8, 10.0, 6.7 Hz, 1H), 2.27 (tt, *J* = 9.6, 4.7 Hz, 1H), 1.75 (dtd, *J* = 13.7, 9.9, 5.2 Hz, 1H), 1.67 – 1.53 (m, 2H), 1.46 (ddd, *J* = 14.1, 9.7, 4.6 Hz, 1H), 1.08 (ddd, *J* = 13.9, 9.4, 4.9 Hz, 1H), 0.88 (d, *J* = 6.6 Hz, 3H), 0.84 (d, *J* = 6.5 Hz, 3H); **^{13}C NMR** ($CDCl_3$, 100 MHz) δ (ppm): 142.2, 140.0, 128.5, 128.4, 125.8, 117.7, 46.9, 42.9, 35.6, 33.3, 25.1, 23.5, 21.6; **HRMS** (EI) mass calculated for $[M]^+$ ($C_{15}H_{21}Br$) requires *m/z* 280.0827, found *m/z* 280.0832.

(*S*)-(3-Isobutylhex-4-yn-1-yl)benzene (7)

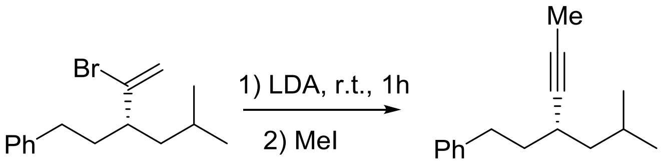

LDA (0.86 M, 0.80 mmol, 0.93 mL in THF) was added slowly to solution of (*S*)-(3-(1-bromovinyl)-5-methylhexyl)benzene **3a** (83 mg, 0.30 mmol) in THF (2.0 mL) at –78 °C. The reaction was removed from the cold bath and stirred at r.t. for 1 h before the addition of MeI (0.50 mmol, 31 µL) and stirring at r.t. for a further 1 h. Saturated $NH_4Cl_{(aq)}$ (10 mL) was added and the aqueous phase was extracted with Et_2O (2 × 15 mL). The combined organic phases were dried over $MgSO_4$, filtered and concentrated *in vacuo*. The crude product was purified by column chromatography (petroleum ether) to afford the title compound as a colourless oil in 93% yield (60 mg).

$[\alpha]_D^{23}$ = –17 (*c* 1.0, $CHCl_3$); **R_f** (petroleum ether): 0.5; **IR** (film) ν_{max}/cm^{-1}: 2953, 2919, 2868, 1495, 1454, 747, 697; **^{1}H NMR** ($CDCl_3$, 400 MHz) δ (ppm): 7.38 – 7.10 (m, 5H), 2.87 (ddd, *J* = 13.6, 9.2, 6.1 Hz, 1H), 2.71 (ddd, *J* = 13.7, 9.5, 7.1 Hz, 1H), 2.42 – 2.34 (m, 1H), 1.89 – 1.82 (m, 4H), 1.78 – 1.63 (m, 2H), 1.43 (ddd, *J* = 13.0, 9.9, 5.0 Hz, 1H), 1.19 (ddd, *J* = 13.1, 9.0, 5.3 Hz, 1H), 0.92 (d, *J* = 6.7 Hz, 3H), 0.88 (d, *J* = 6.7 Hz, 3H); **^{13}C NMR** ($CDCl_3$, 100 MHz) δ (ppm): 142.5, 128.5, 128.3, 125.7, 82.3, 76.9, 44.8, 37.7, 33.8, 29.5, 26.0, 23.4, 21.8, 3.6; **HRMS** (EI) mass calculated for $[M–CH_3]^+$ ($C_{15}H_{19}$) requires *m/z* 199.1487, found *m/z* 199.1480.

(*S*)-2,7-Dimethyl-5-phenethyloct-3-yn-2-ol (8)

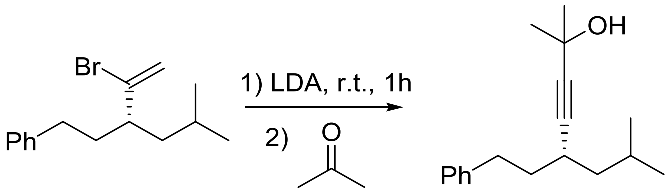

LDA (0.86 M, 0.80 mmol, 0.93 mL in THF) was added slowly to solution of (*S*)-(3-(1-bromovinyl)-5-methylhexyl)benzene **3a** (83 mg, 0.30 mmol) in THF (2.0 mL) at –78 °C. The reaction was removed from the cold bath and stirred at r.t. for 1 h before the addition of acetone (0.50 mmol, 37 µL) and stirring at r.t. for a further 4 h. Saturated $NH_4Cl_{(aq)}$ (10 mL) was added and the aqueous phase was extracted with Et_2O (2 × 15 mL). The combined organic phases were dried over $MgSO_4$, filtered and concentrated *in vacuo*. The crude product was purified by column chromatography (petroleum ether/ethyl acetate = 5:1) to afford the title compound as a colourless oil in 83% yield (64 mg).

$[\alpha]_D^{23}$ = –26 (*c* 1.0, $CHCl_3$); **R_f** (petroleum ether/ethyl acetate = 10:1): 0.2; **IR** (film) ν_{max}/cm^{-1}: 2954, 2926, 1455, 1366, 1165, 941, 746, 698; **^{1}H NMR** ($CDCl_3$, 300 MHz) δ (ppm): 7.32 – 7.15 (m, 5H), 2.88 – 2.74 (m, 1H), 2.74 – 2.61 (m, 1H), 2.46 – 2.32 (m, 1H), 1.85 – 1.68 (m, 3H), 1.51 (s, 6H), 1.47 – 1.33 (m, 1H), 1.27 – 1.14 (m, 2H), 0.88 (d, *J* = 6.6 Hz, 3H), 0.87 (d, *J* = 6.6 Hz, 3H); **^{13}C NMR** ($CDCl_3$, 75 MHz) δ (ppm): 142.3, 128.5, 128.4, 125.8, 86.8, 85.5, 65.4, 44.3, 37.2, 33.7, 31.9, 29.2, 26.0, 23.3, 21.9; **HRMS** (EI) mass calculated for $[M]^+$ ($C_{18}H_{26}O$) requires *m/z* 258.1984, found *m/z* 258.1988.

(*S*)-Triisopropyl(5-methyl-3-phenethylhex-1-yn-1-yl)silane (9)

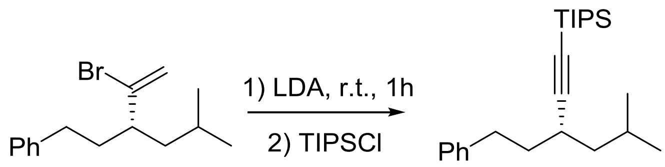

LDA (0.86 M, 0.80 mmol, 0.93 mL in THF) was added slowly to solution of (*S*)-(3-(1-bromovinyl)-5-methylhexyl)benzene **3a** (83 mg, 0.30 mmol) in THF (2.0 mL) at –78 °C. The reaction was removed from the cold bath and stirred at r.t. for 1 h before the addition of TIPSCl (0.50 mmol, 96 mg) and stirring at r.t. for a further 1 h. Saturated $NH_4Cl_{(aq)}$ (10 mL) was added and the aqueous phase was extracted with Et_2O (2 × 15 mL). The combined organic phases were dried over $MgSO_4$, filtered and concentrated *in vacuo*. The crude product was purified by column chromatography (petroleum ether) to afford the title compound as a colourless oil in 95% yield (102 mg).

$[\alpha]_D^{23}$ = –18 (*c* 1.0, $CHCl_3$); R_f (petroleum ether): 0.8; **IR** (film) ν_{max}/cm^{-1}: 2941, 2864, 1463, 995, 882, 746, 660; **^{1}H NMR** ($CDCl_3$, 300 MHz) δ (ppm): 7.31 – 7.14 (m, 5H), 2.90 (ddd, *J* = 13.6, 9.3, 5.9 Hz, 1H), 2.73 (ddd, *J* = 13.6, 9.6, 7.3 Hz, 1H), 2.45 (ddt, *J* = 10.2, 8.6, 5.3 Hz, 1H), 1.88 (dtt, *J* = 11.7, 9.0, 5.8 Hz, 1H), 1.80 – 1.62 (m, 2H), 1.46 (ddd, *J* = 13.0, 10.0, 5.1 Hz, 1H), 1.35 – 0.94 (m, 22H), 0.90 (d, *J* = 6.6 Hz, 3H), 0.87 (d, *J* = 6.6 Hz, 3H); **^{13}C NMR** ($CDCl_3$, 75 MHz) δ (ppm): 142.5, 128.5, 128.4, 125.8, 112.3, 81.4, 44.5, 37.8, 33.8, 30.6, 26.1, 23.4, 21.8, 18.7, 11.4; **HRMS** (EI) mass calculated for $[M–C_3H_7]^+$ ($C_{21}H_{33}Si$) requires *m/z* 313.2352, found *m/z* 313.2360.

7. ^{1}H and ^{13}C NMR spectra

va/yw35722-wyh-2-89
single_pulse

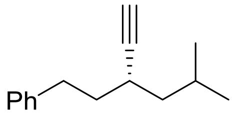

6a

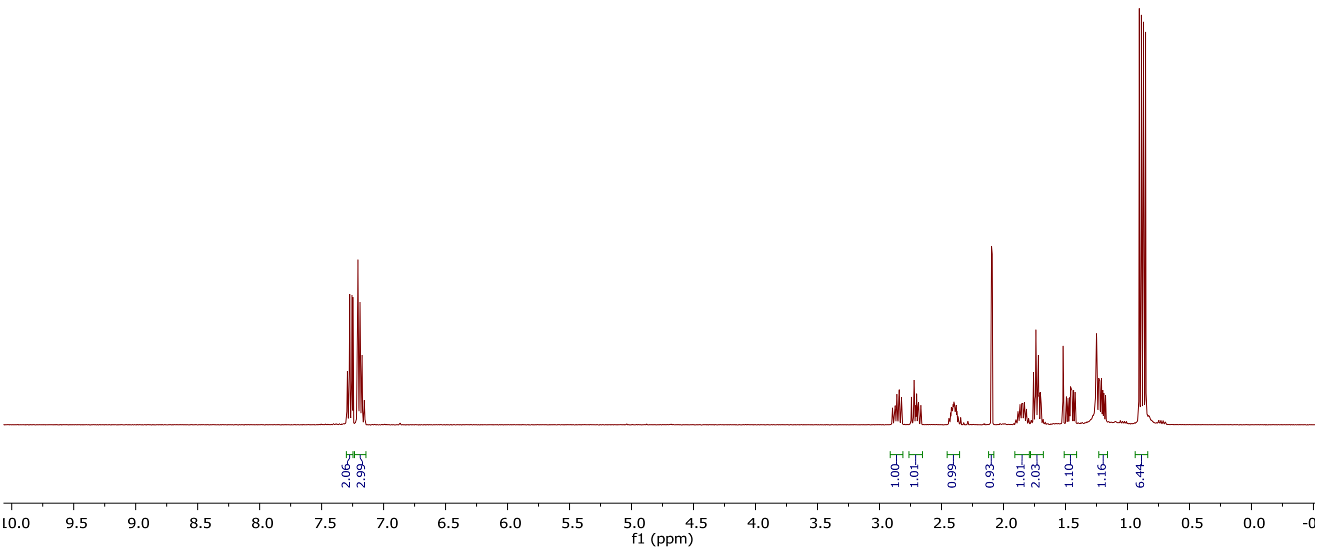

va/yw35722-wyh-2-89
single pulse decoupled gated NOE

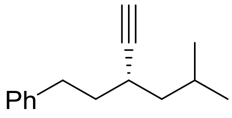

6a

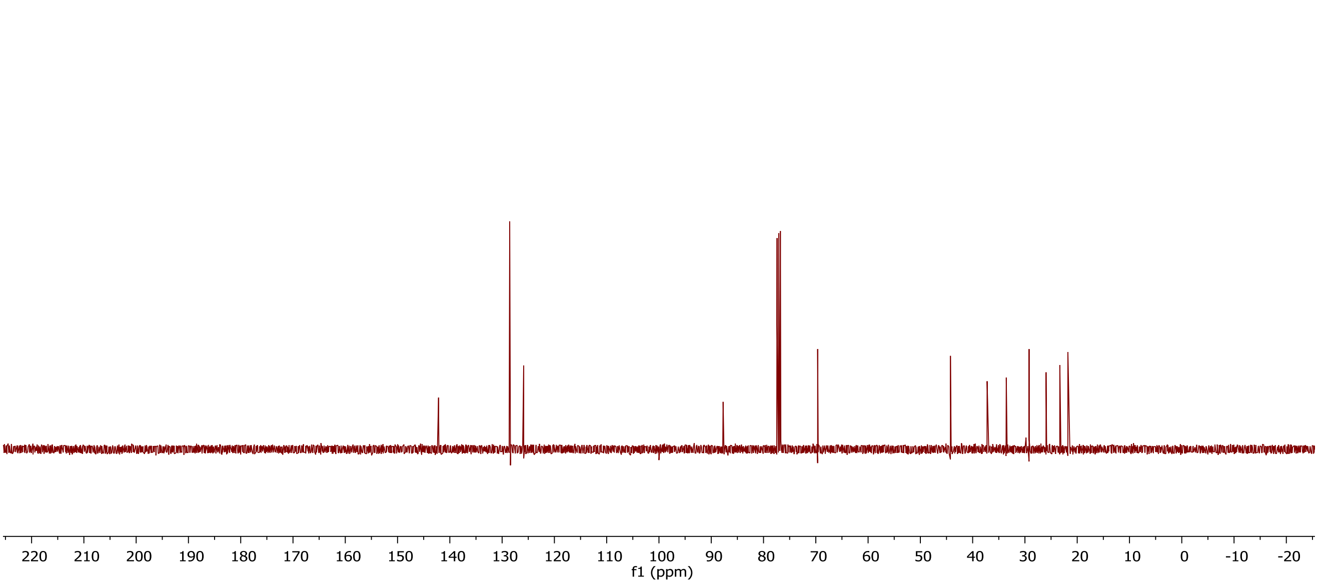

va/yw19099-wyh-3-8
single_pulse

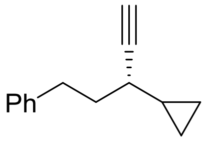

6b

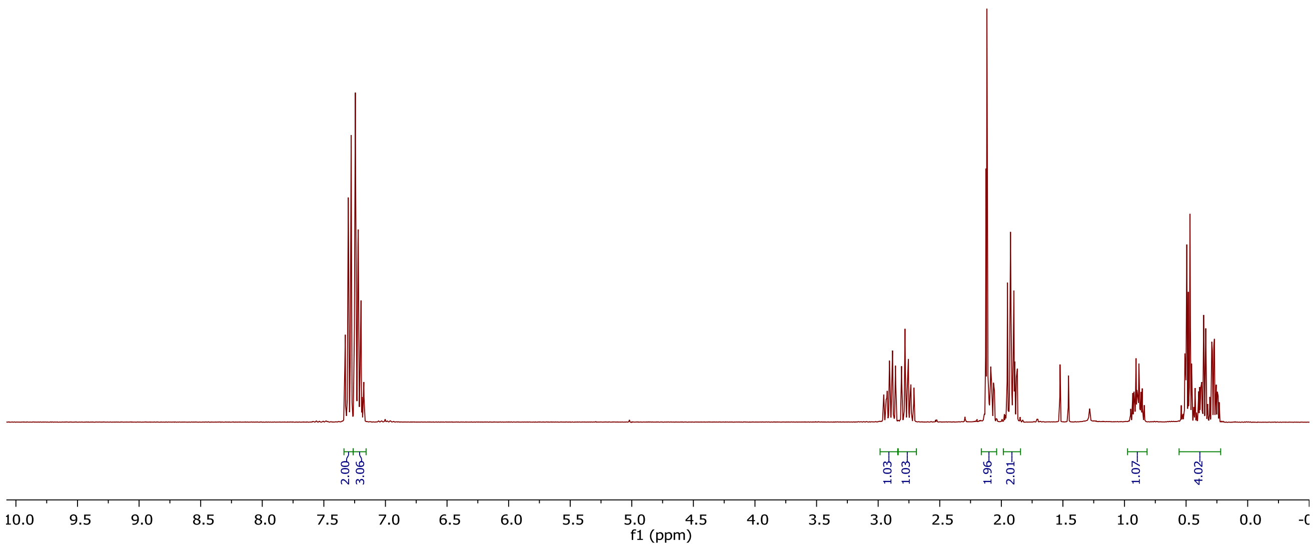

va/yw19099-wyh-3-8
single pulse decoupled gated NOE

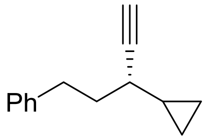

6b

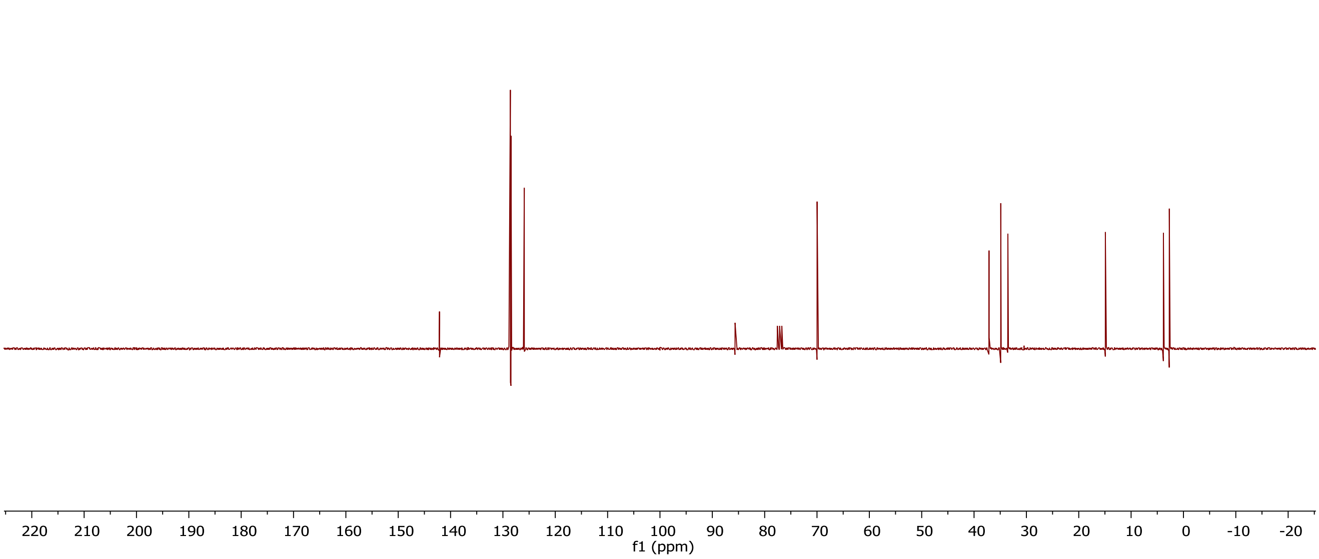

va/yw36501-wyh-2-113
single_pulse

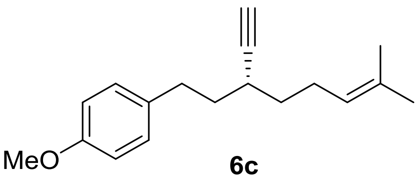

6c

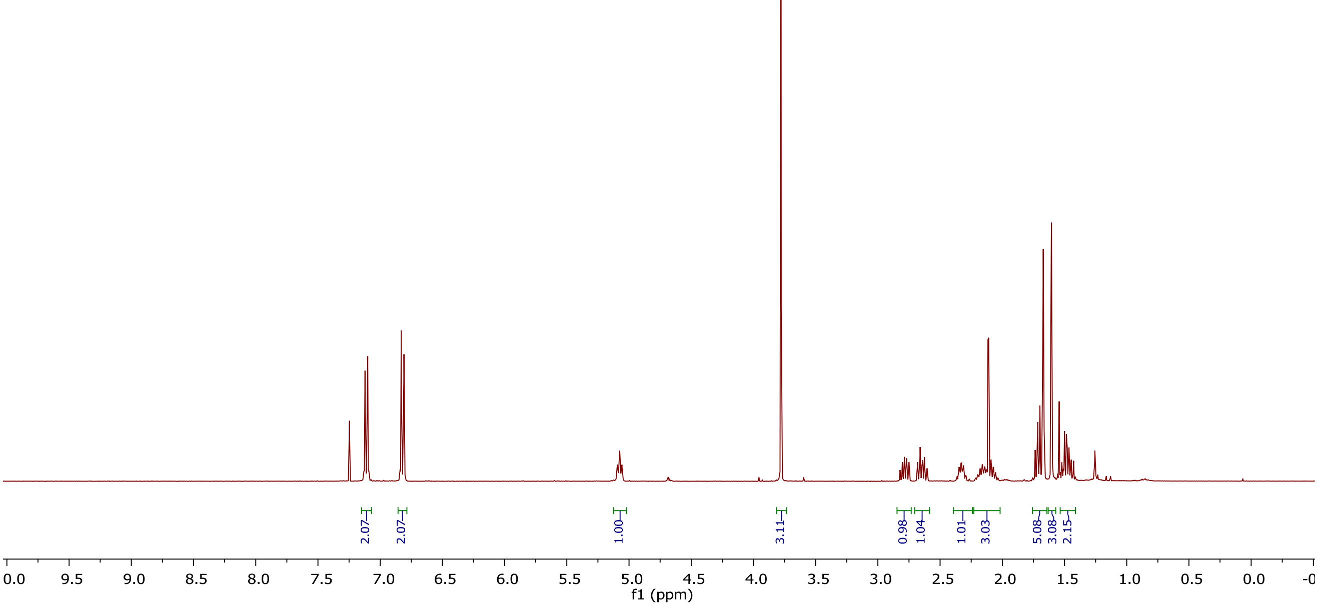

wyh-2-111_CARBON_01

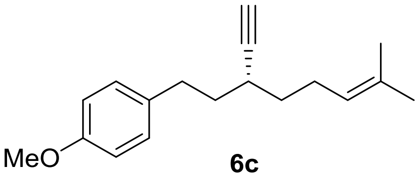

6c

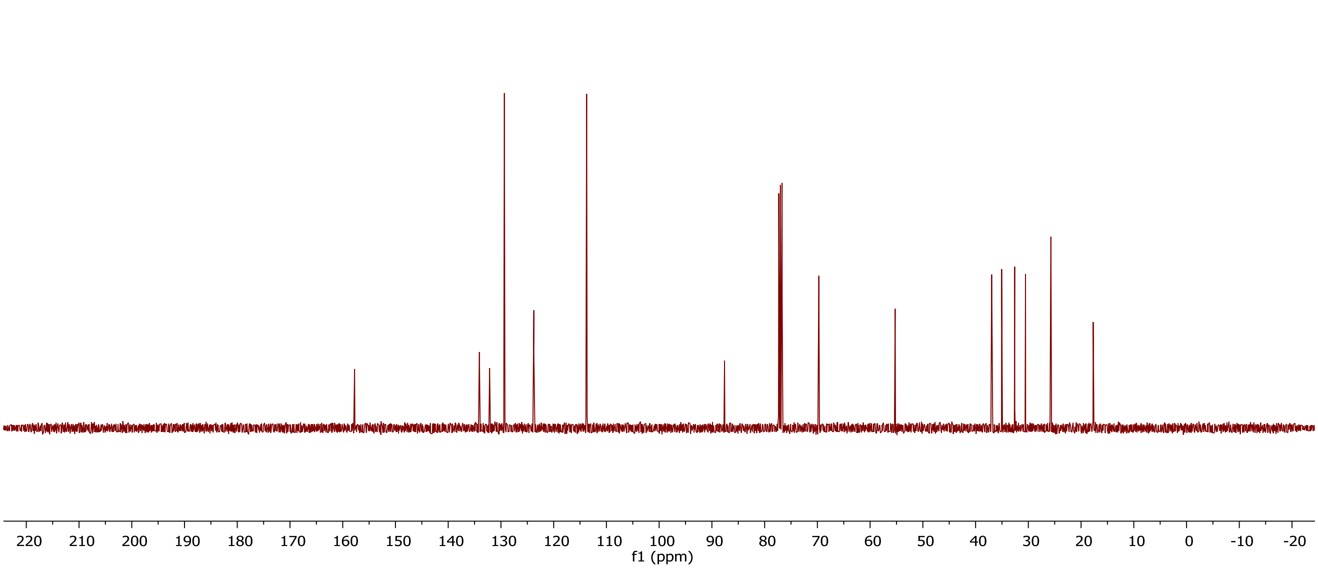

va/yw35723-wyh-2-90
single_pulse

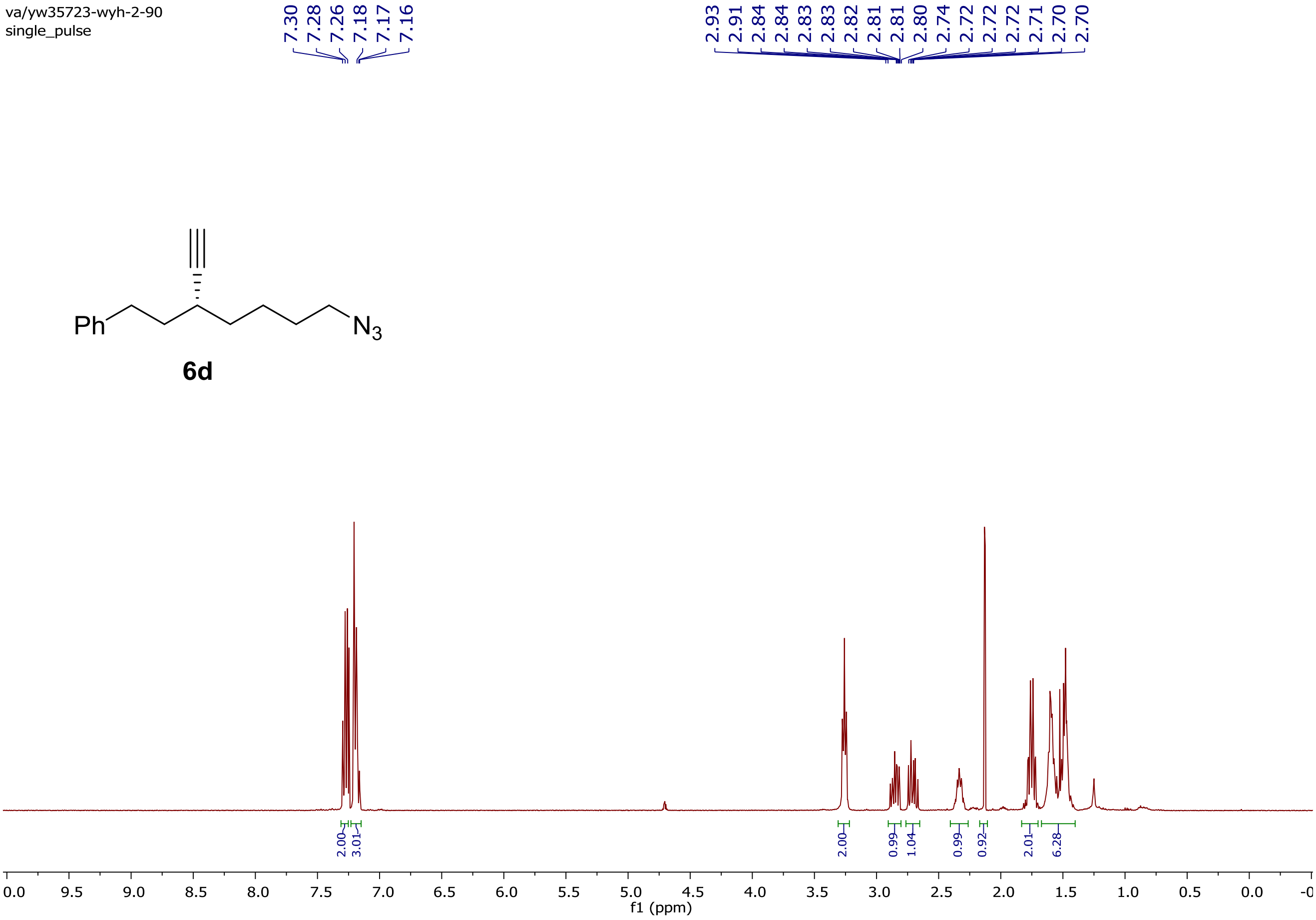

va/yw35795-wyh-2-90
single pulse decoupled gated NOE

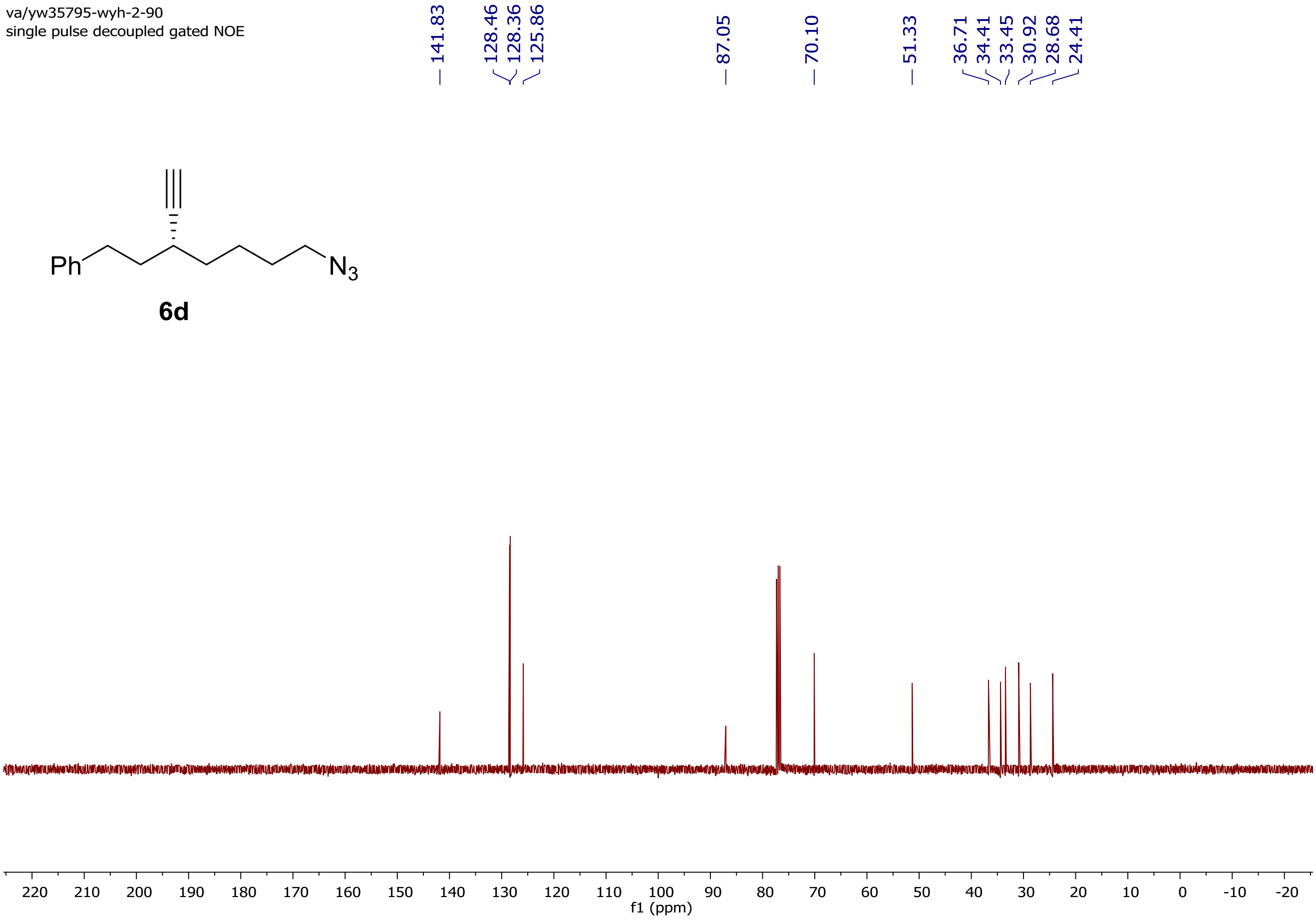

va/yw17051-wyh-2-120
single_pulse

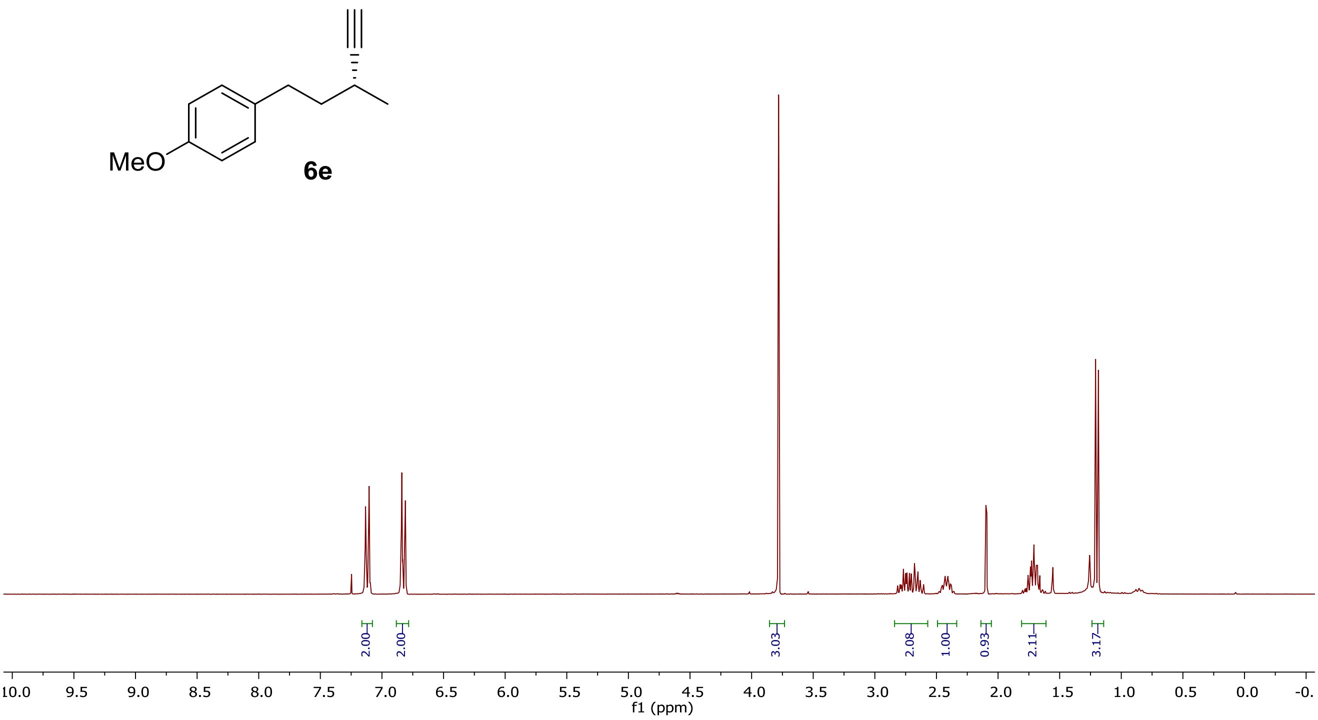

va/yw17051-wyh-2-120
single pulse decoupled gated NOE

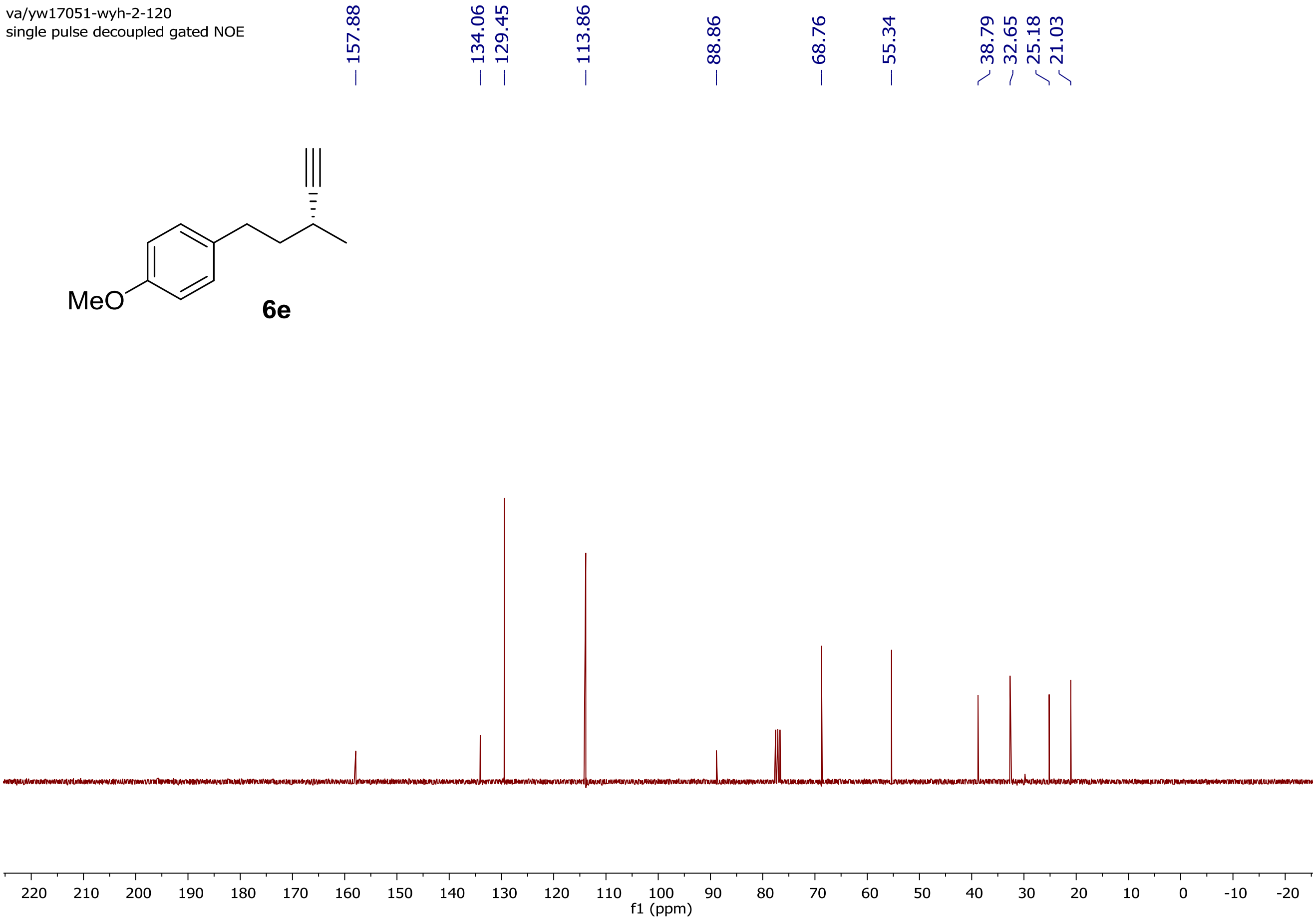

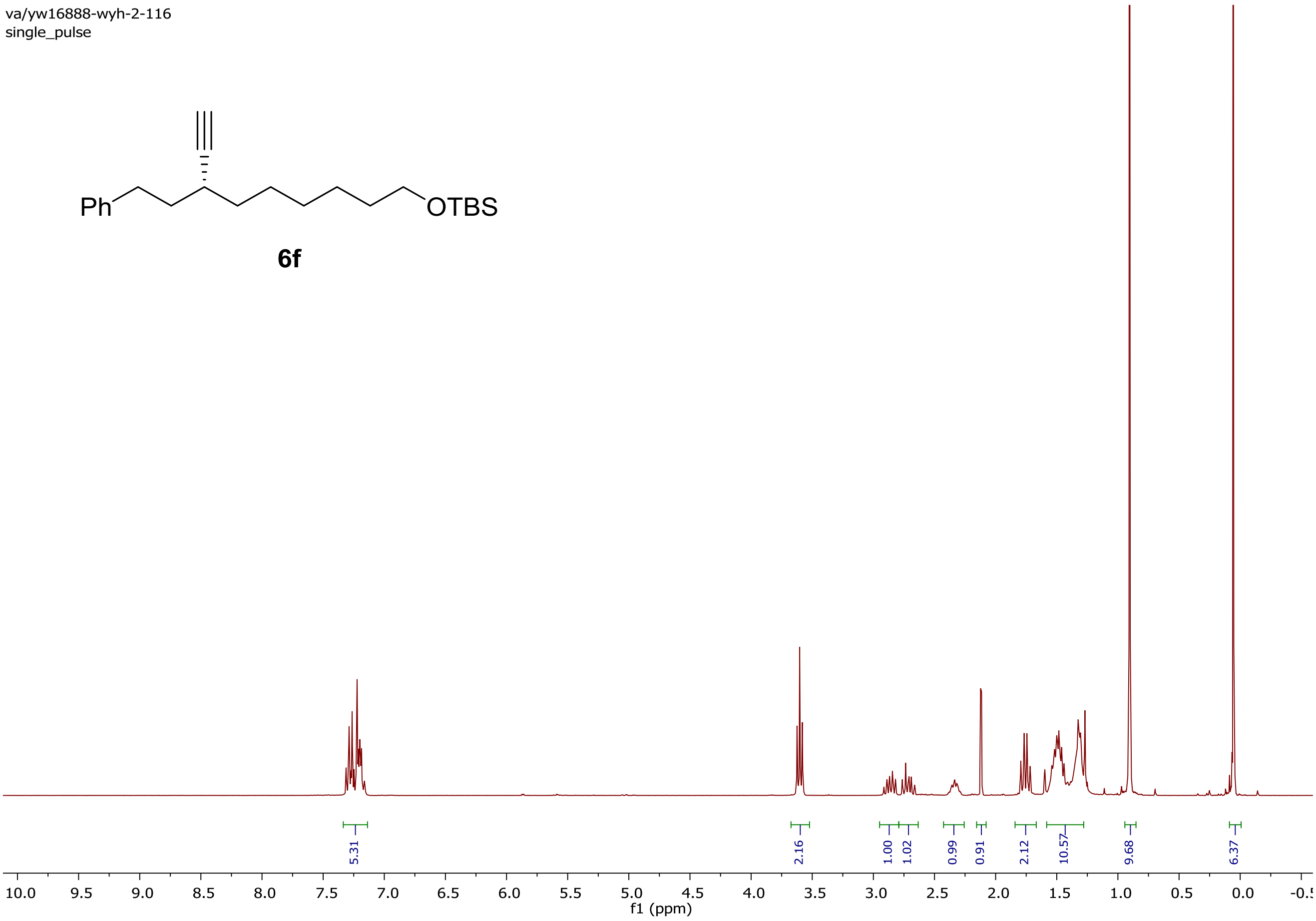
va/yw16888-wyh-2-116
single_pulse
Ph
OTBS
6f
5.31
2.16
1.00
1.02
0.99
0.91
2.12
10.57
9.68
6.37
f1 (ppm)

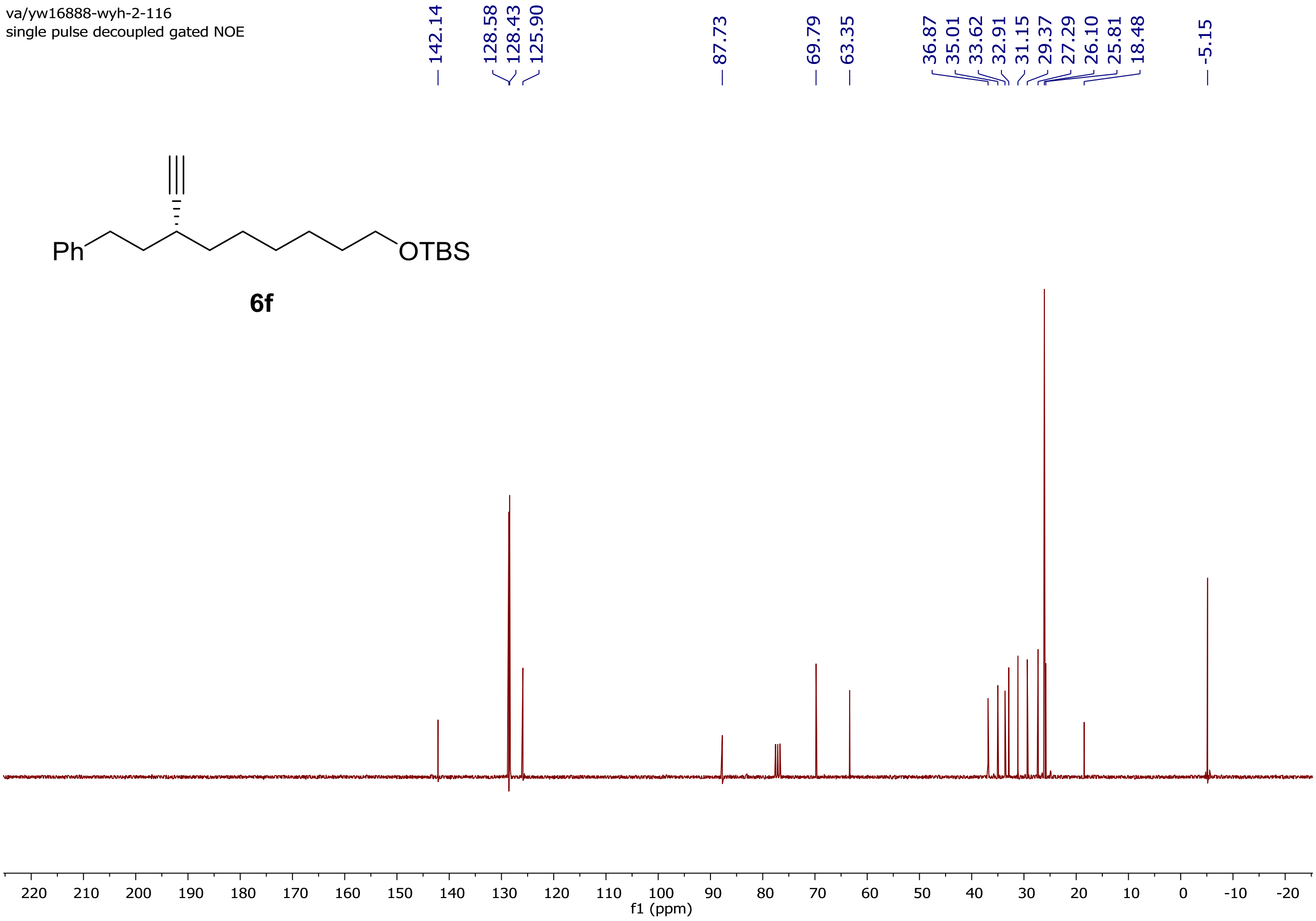
va/yw16888-wyh-2-116
single pulse decoupled gated NOE
142.14
128.58
128.43
125.90
87.73
69.79
63.35
36.87
35.01
33.62
32.91
31.15
29.37
27.29
26.10
25.81
18.48
-5.15
Ph
OTBS
6f
f1 (ppm)

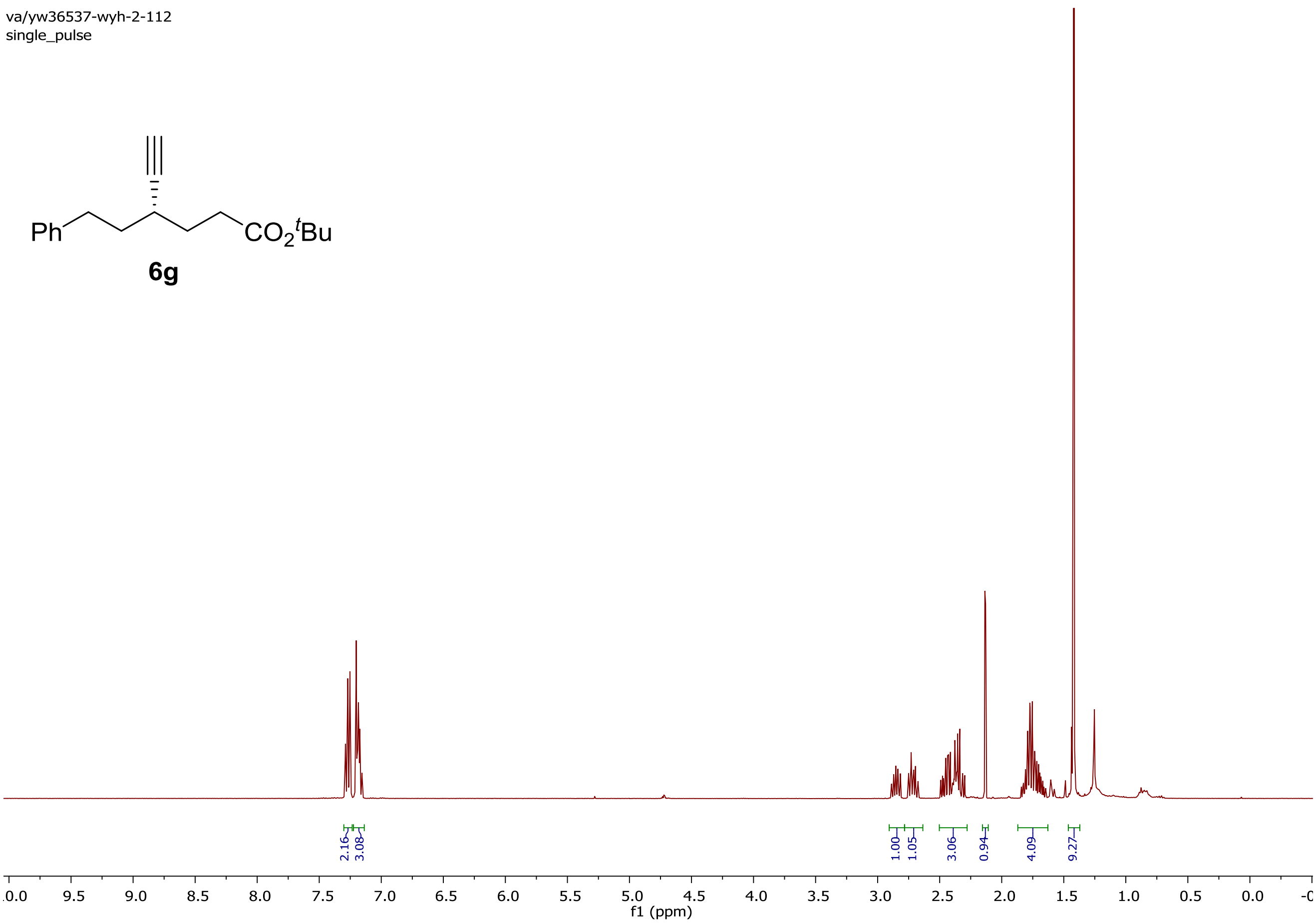
va/yw36537-wyh-2-112
single_pulse
Ph
$CO_2{}^tBu$
6g
2.16
3.08
1.00
1.05
3.06
0.94
4.09
9.27
f1 (ppm)

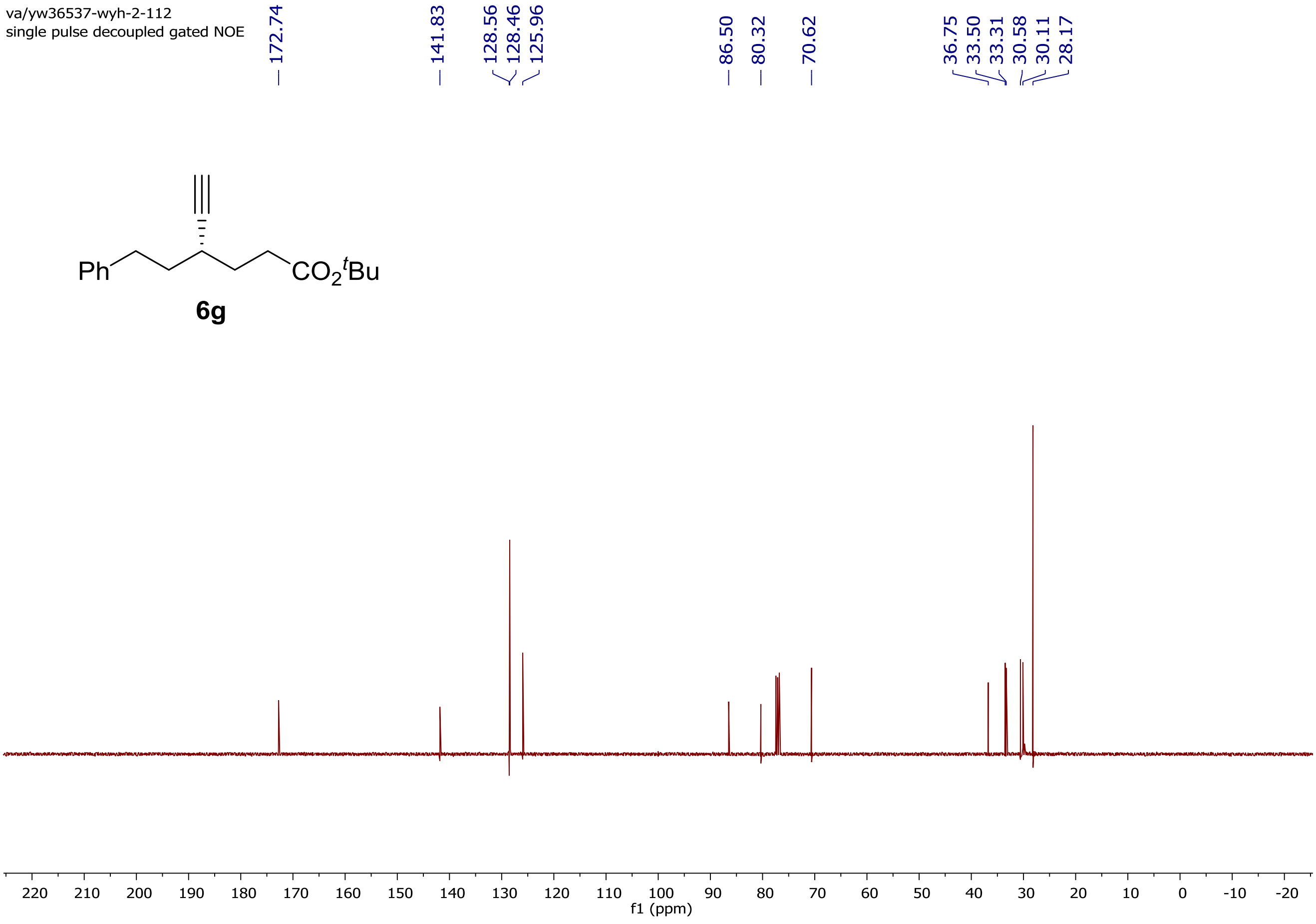
va/yw36537-wyh-2-112
single pulse decoupled gated NOE
172.74
141.83
128.56
128.46
125.96
86.50
80.32
70.62
36.75
33.50
33.31
30.58
30.11
28.17
Ph
$CO_2{}^tBu$
6g
f1 (ppm)

an141175_AN-3-551_PROTON_01

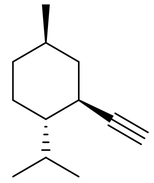

6h

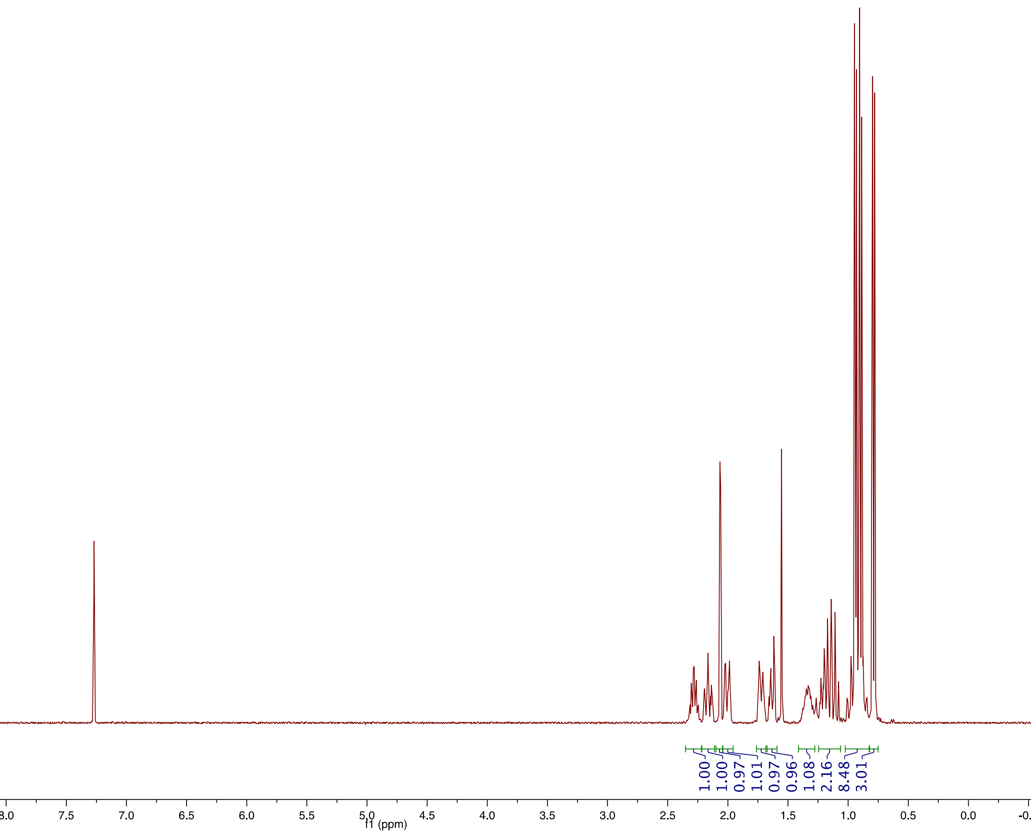

an21723_AN-3-551_CARBON_001

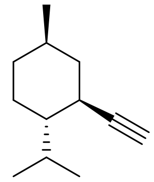

6h

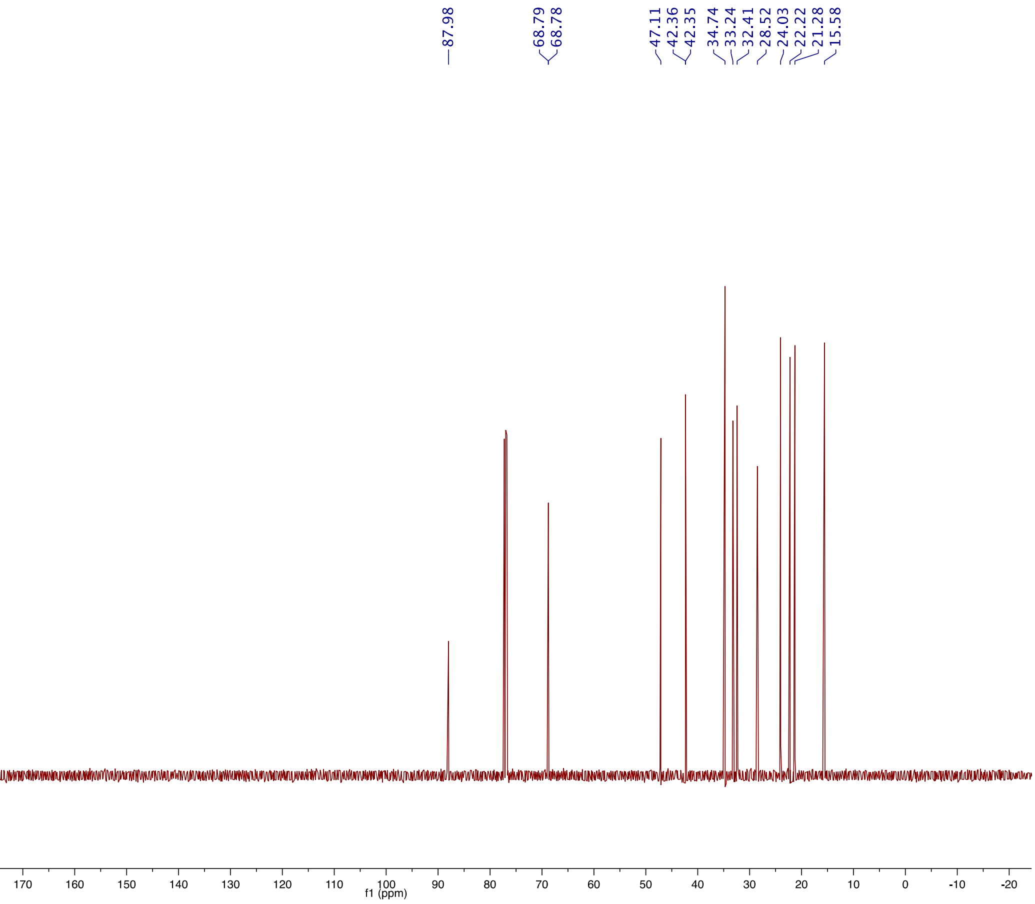

an21739_AN-3-552_PROTON_001

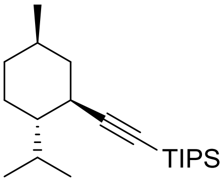

6h'

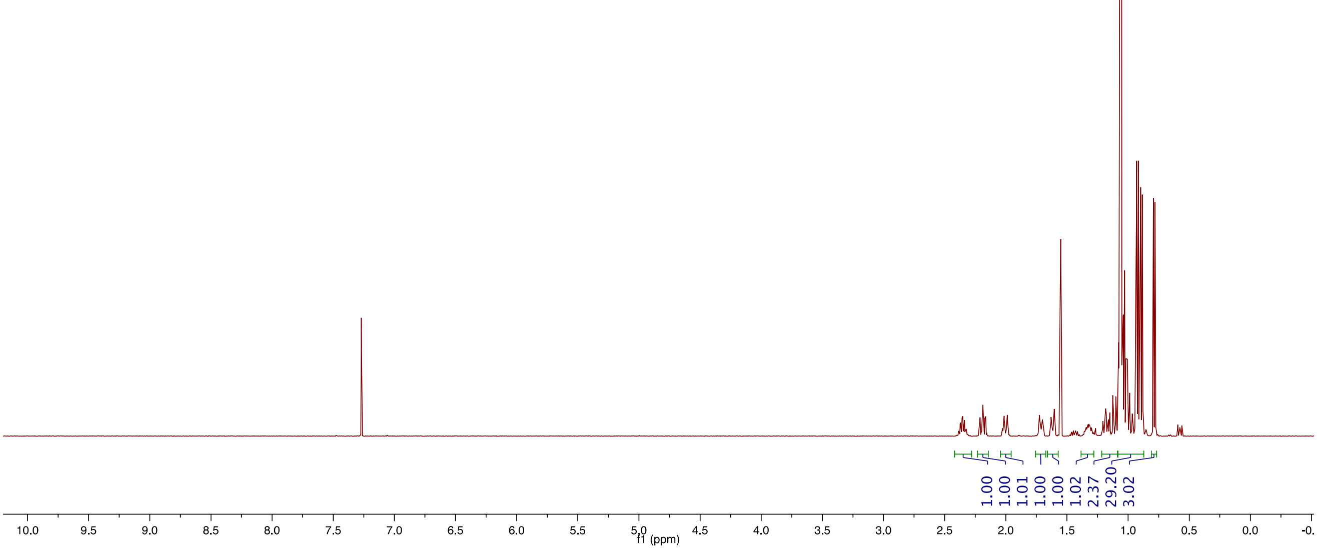

an21739_AN-3-552_CARBON_001

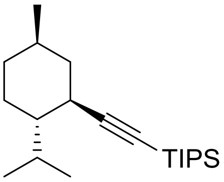

6h'

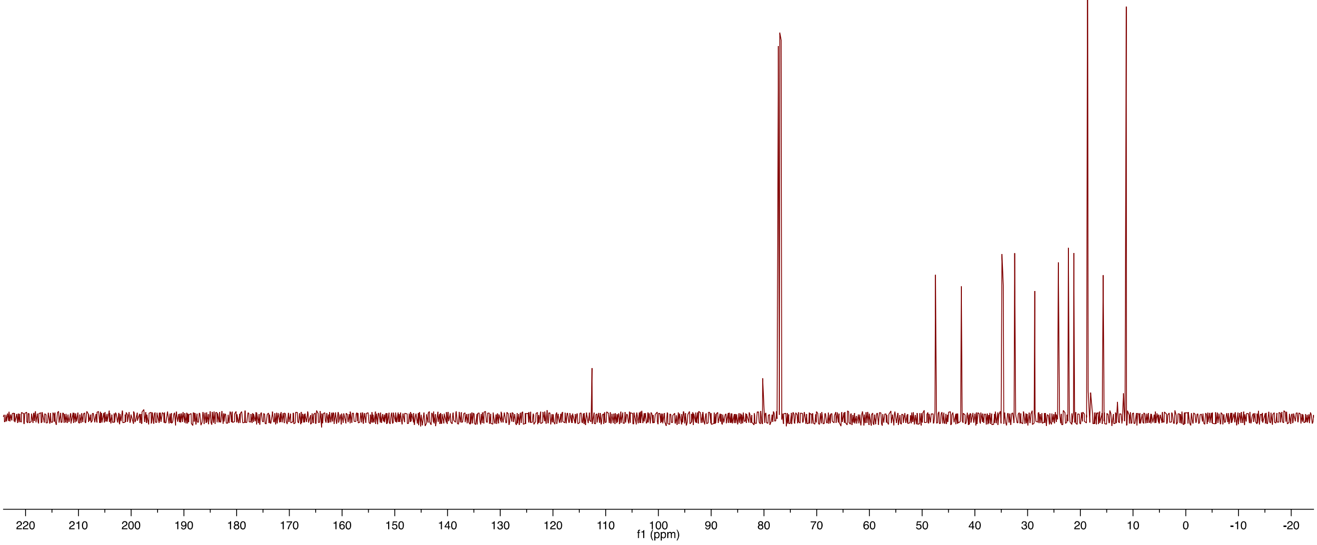

va/yw35980-wyh-2-95
single_pulse

6i

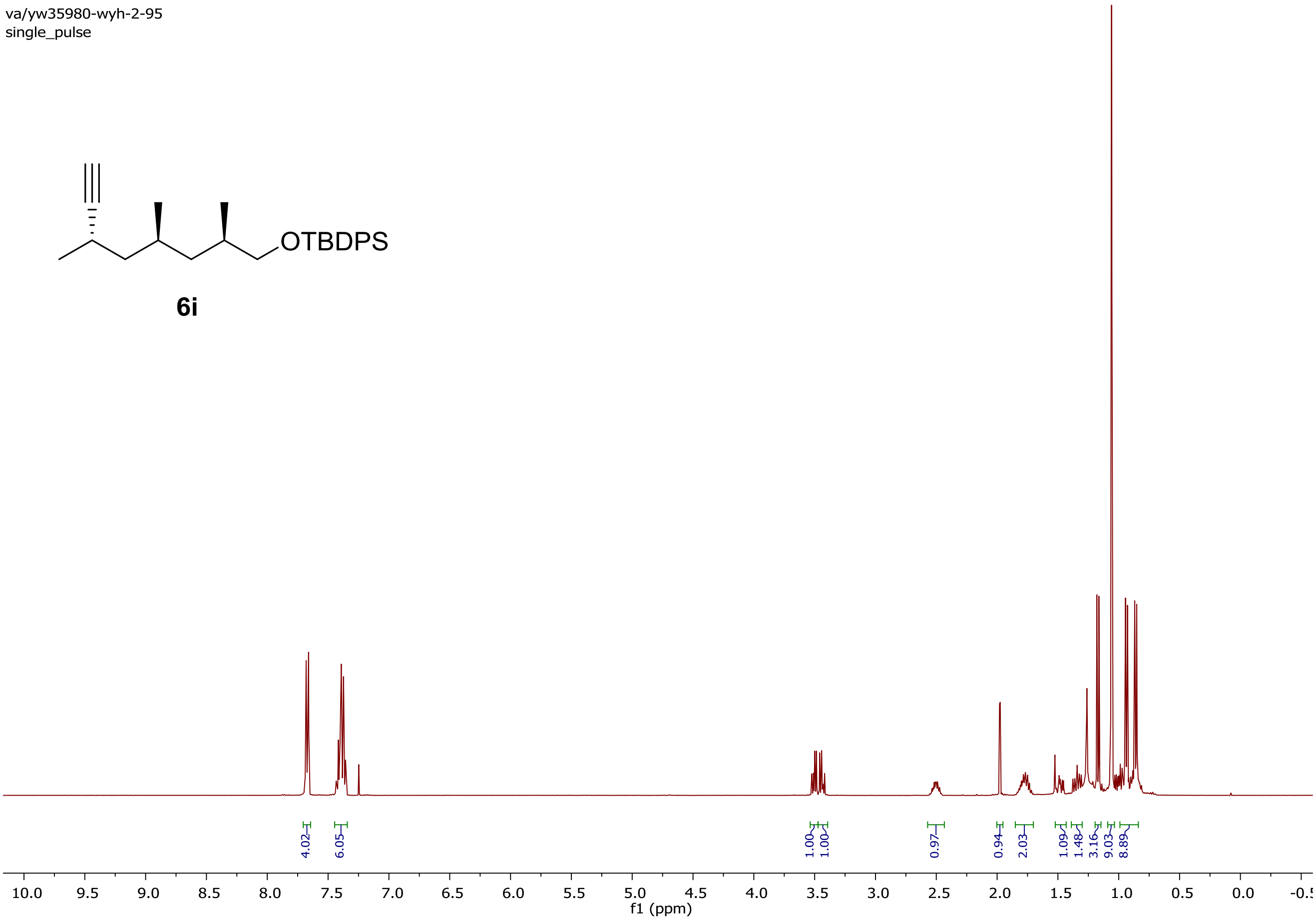

va/yw35980-wyh-2-95
single pulse decoupled gated NOE

6i

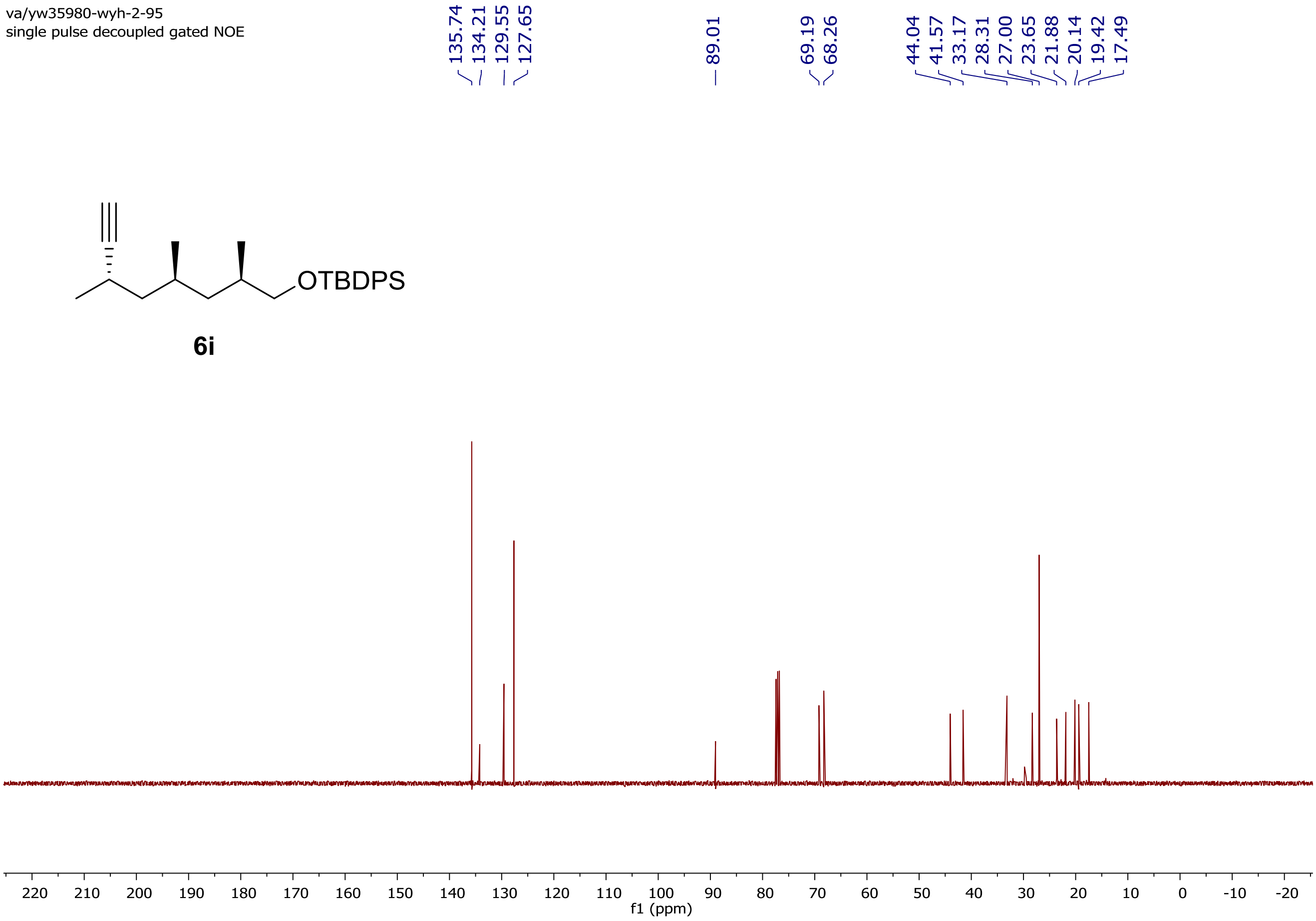

va/yw37538-wyh-2-161
single_pulse

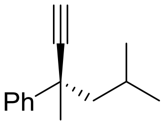

6j

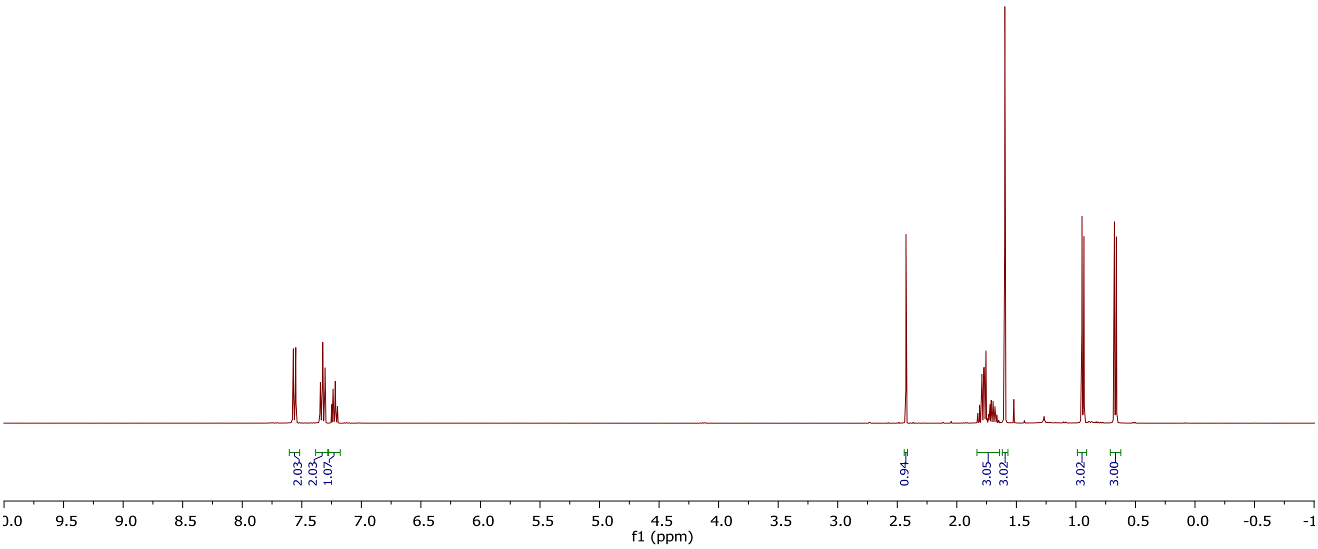

va/yw37825-wyh-2-167
single pulse decoupled gated NOE

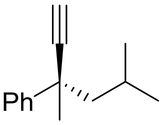

6j

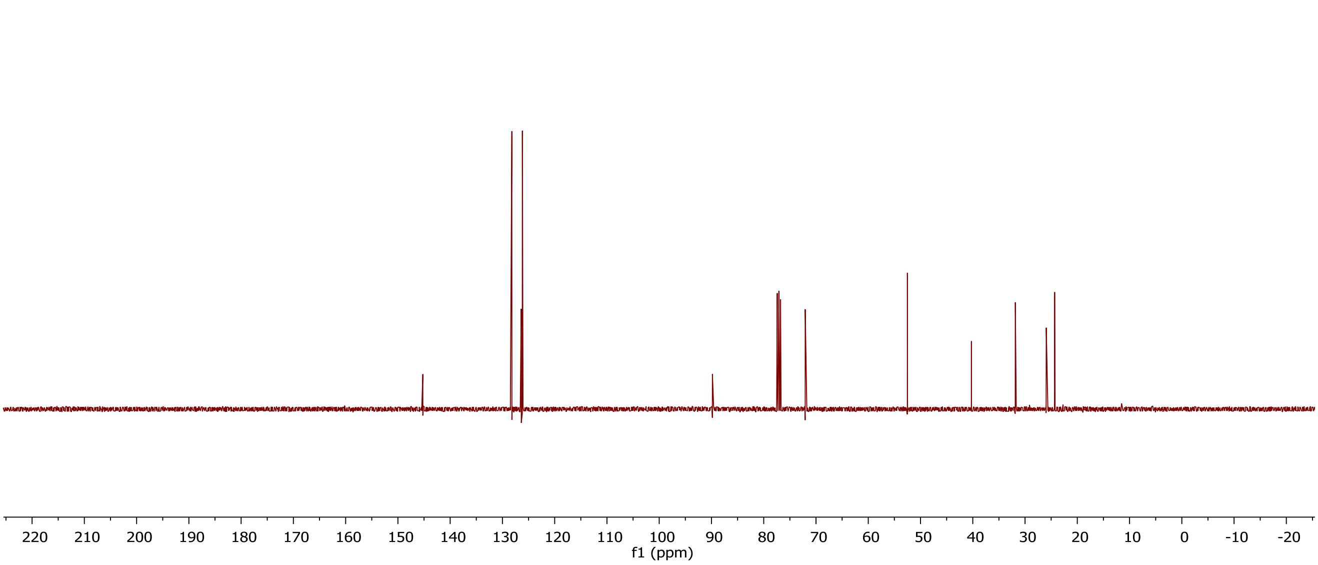

va/yw37672-wyh-2-164
single_pulse

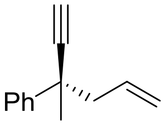

6k

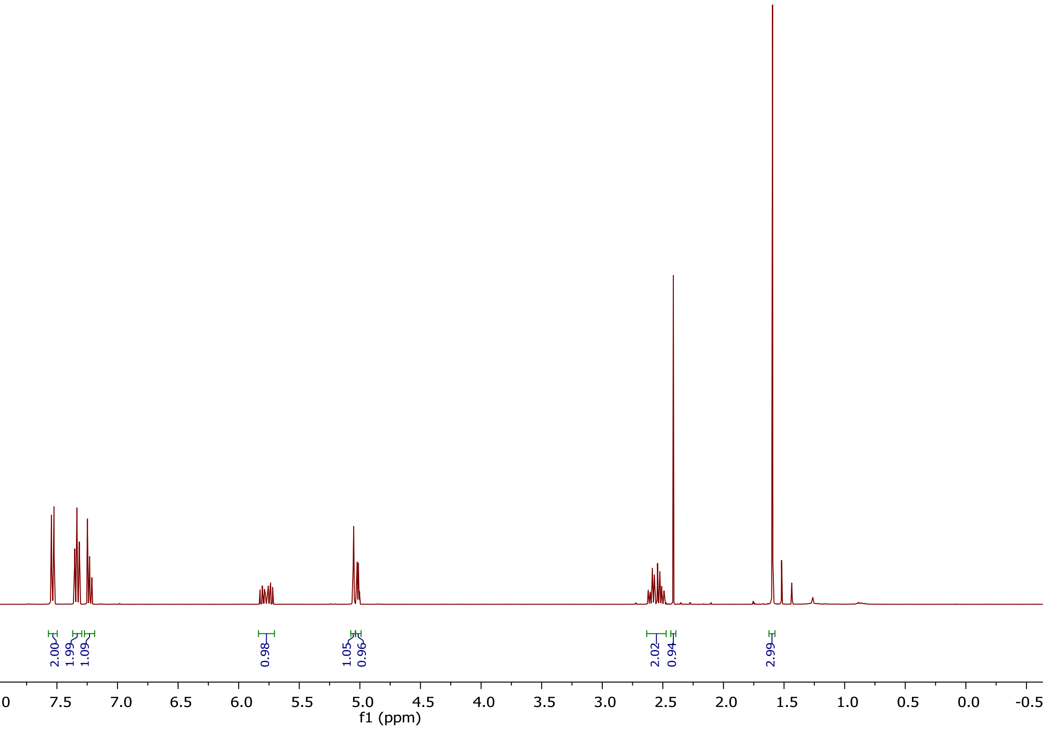

va/yw19013-wyh-3-7
single pulse decoupled gated NOE

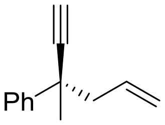

6k

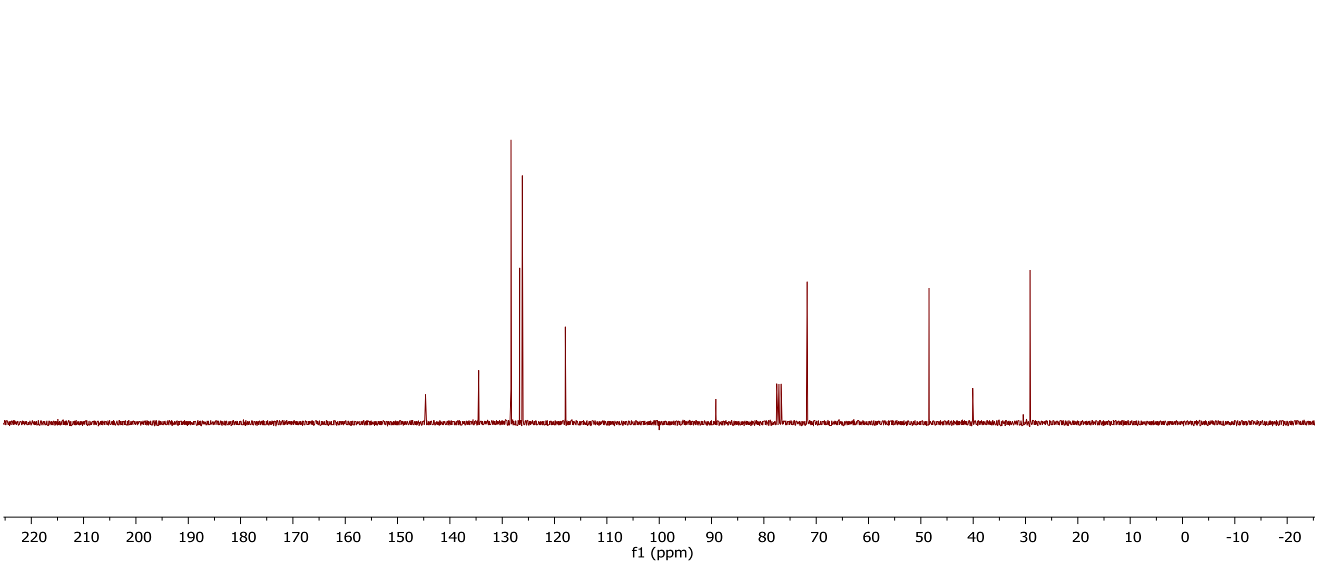

va/yw39431-wyh-3-1
single_pulse

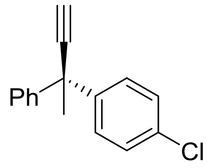

6l

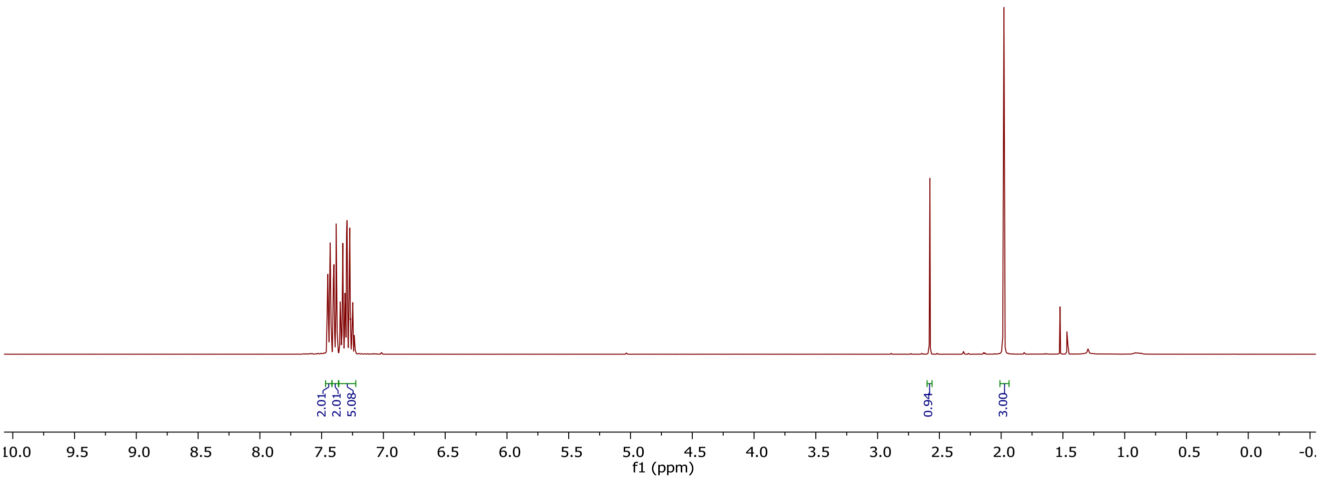

va/yw39431-wyh-3-1
single pulse decoupled gated NOE

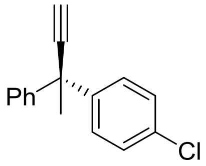

6l

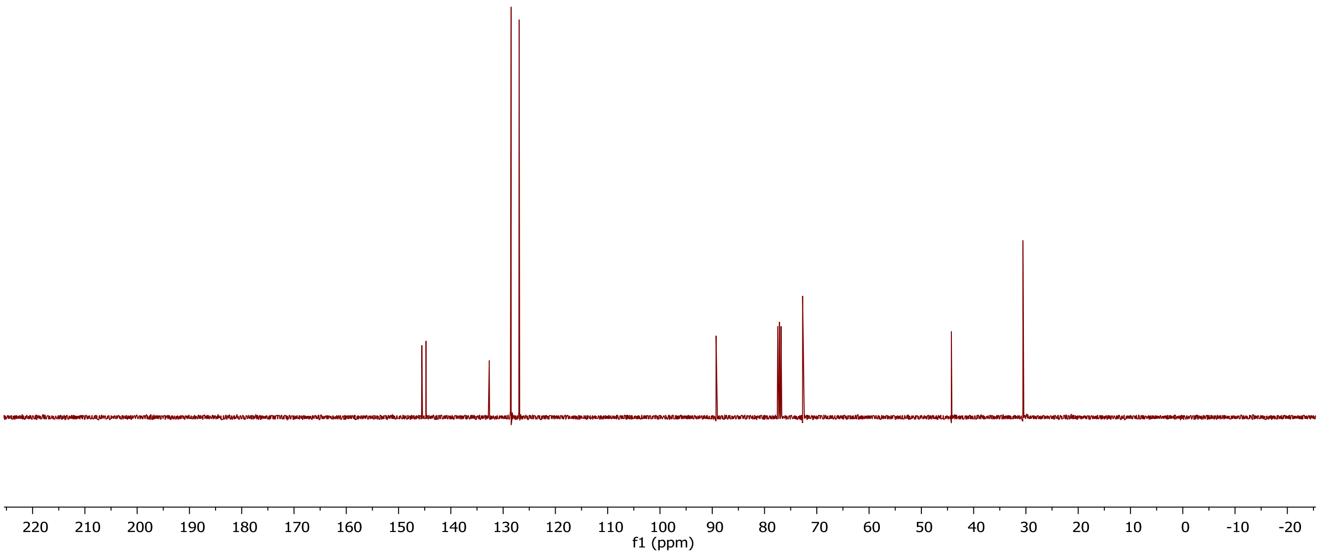

wyh-2-173_PROTON_01

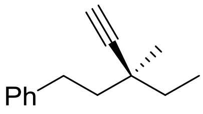

6m

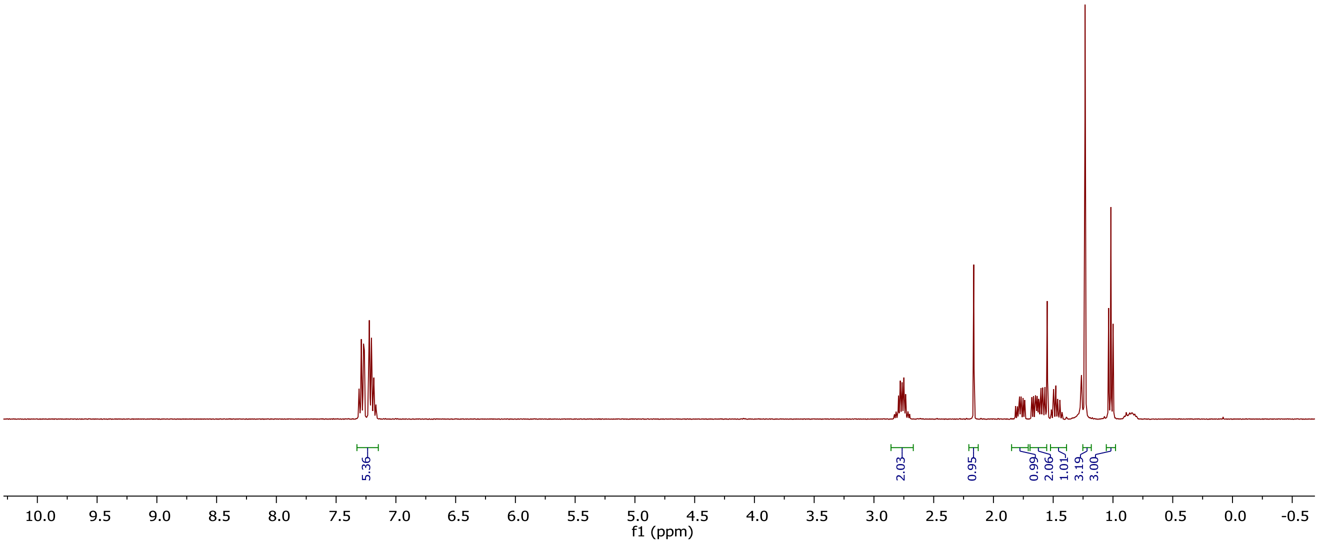

yw139445_wyh-2-173_CARBON_01

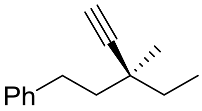

6m

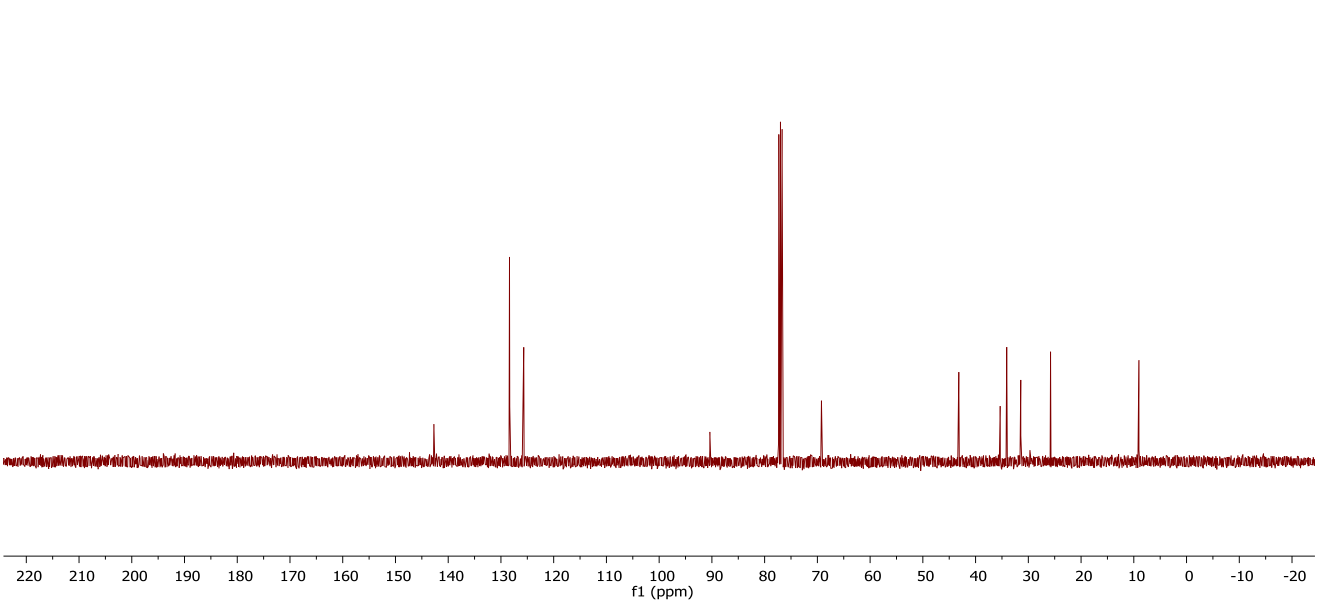

va/yw39432-wyh-2-188
single_pulse

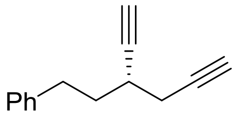

6n

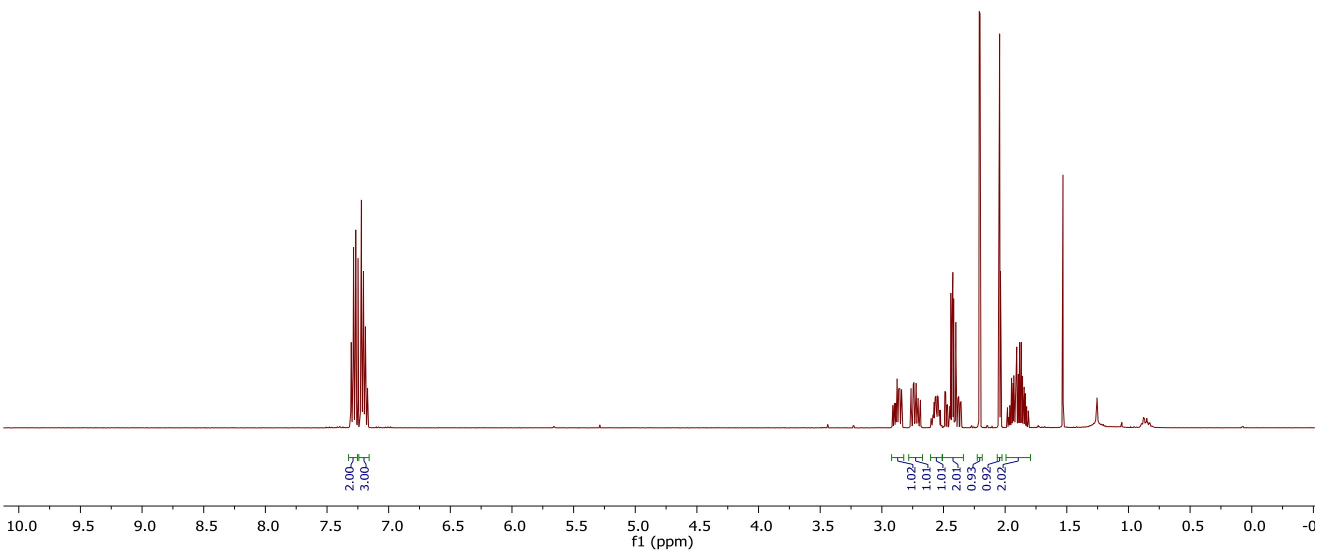

va/yw39432-wyh-2-188
single pulse decoupled gated NOE

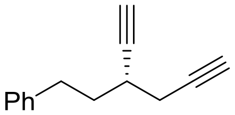

6n

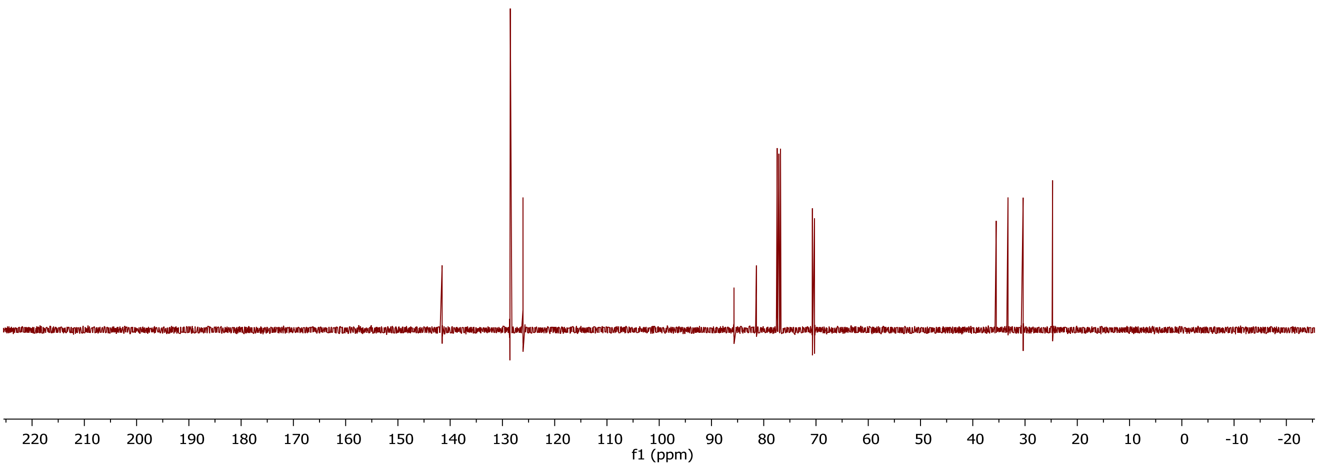

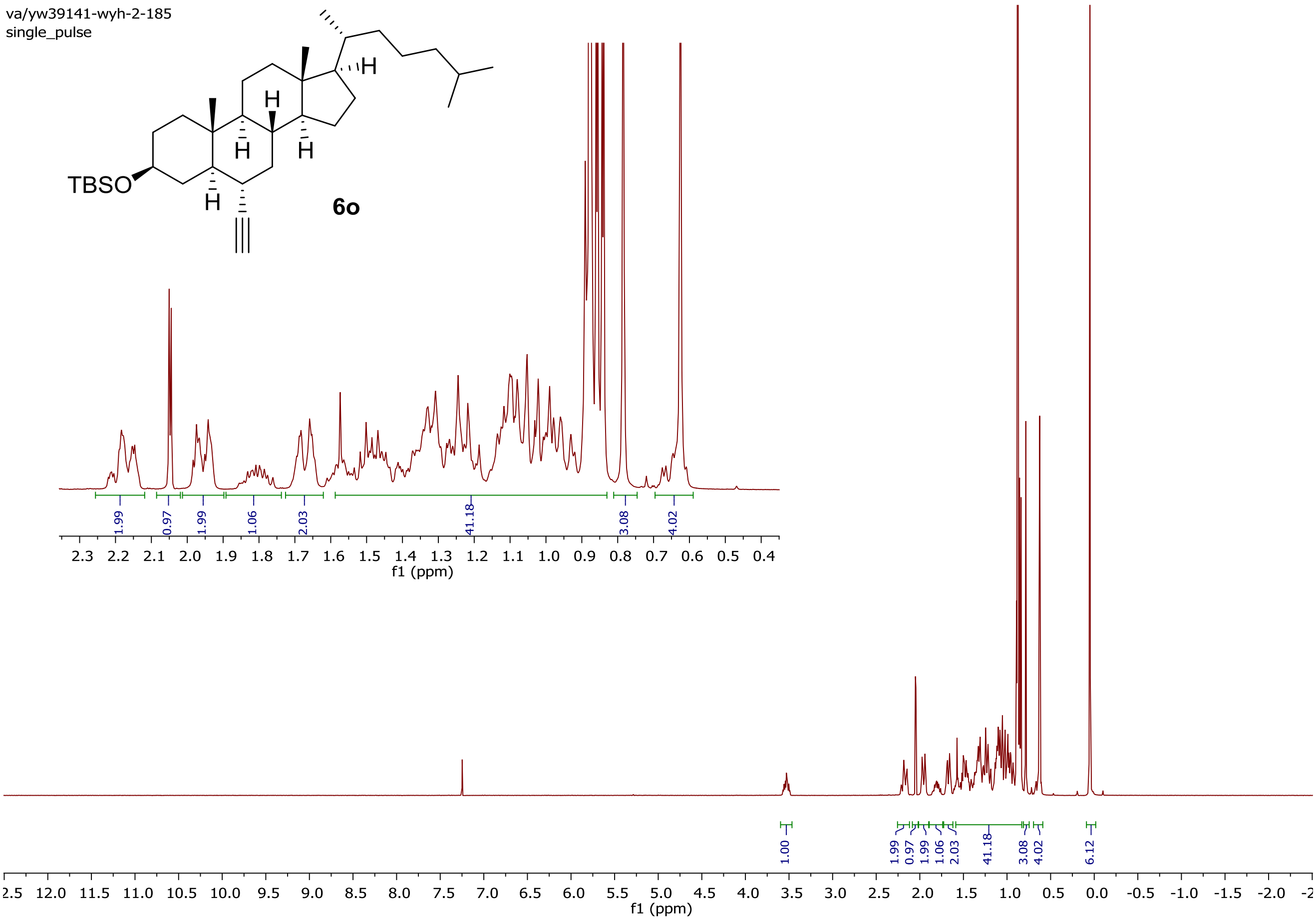
va/yw39141-wyh-2-185
single_pulse
TBSO
6o
1.99 0.97 1.99 1.06 2.03 41.18 3.08 4.02
1.00 1.99 0.97 1.99 1.06 2.03 41.18 3.08 4.02 6.12
f1 (ppm)

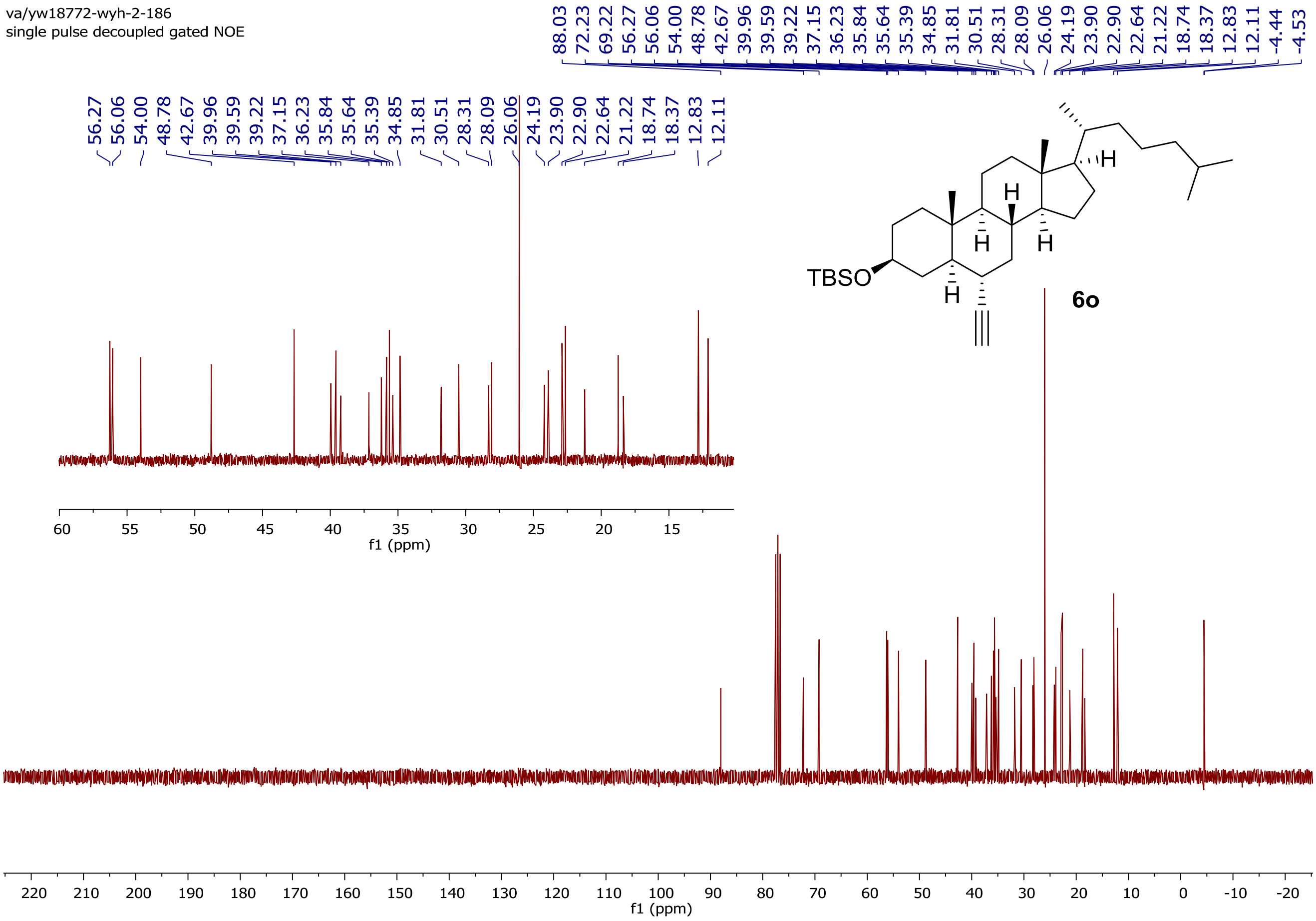
va/yw18772-wyh-2-186
single pulse decoupled gated NOE
88.03 72.23 69.22 56.27 56.06 54.00 48.78 42.67 39.96 39.59 39.22 37.15 36.23 35.84 35.64 35.39 34.85 31.81 30.51 28.31 28.09 26.06 24.19 23.90 22.90 22.64 21.22 18.74 18.37 12.83 12.11 -4.44 -4.53
56.27 56.06 54.00 48.78 42.67 39.96 39.59 39.22 37.15 36.23 35.84 35.64 35.39 34.85 31.81 30.51 28.31 28.09 26.06 24.19 23.90 22.90 22.64 21.22 18.74 18.37 12.83 12.11
TBSO
6o
f1 (ppm)

va/yw19836-wyh-3-24-f-s
single_pulse

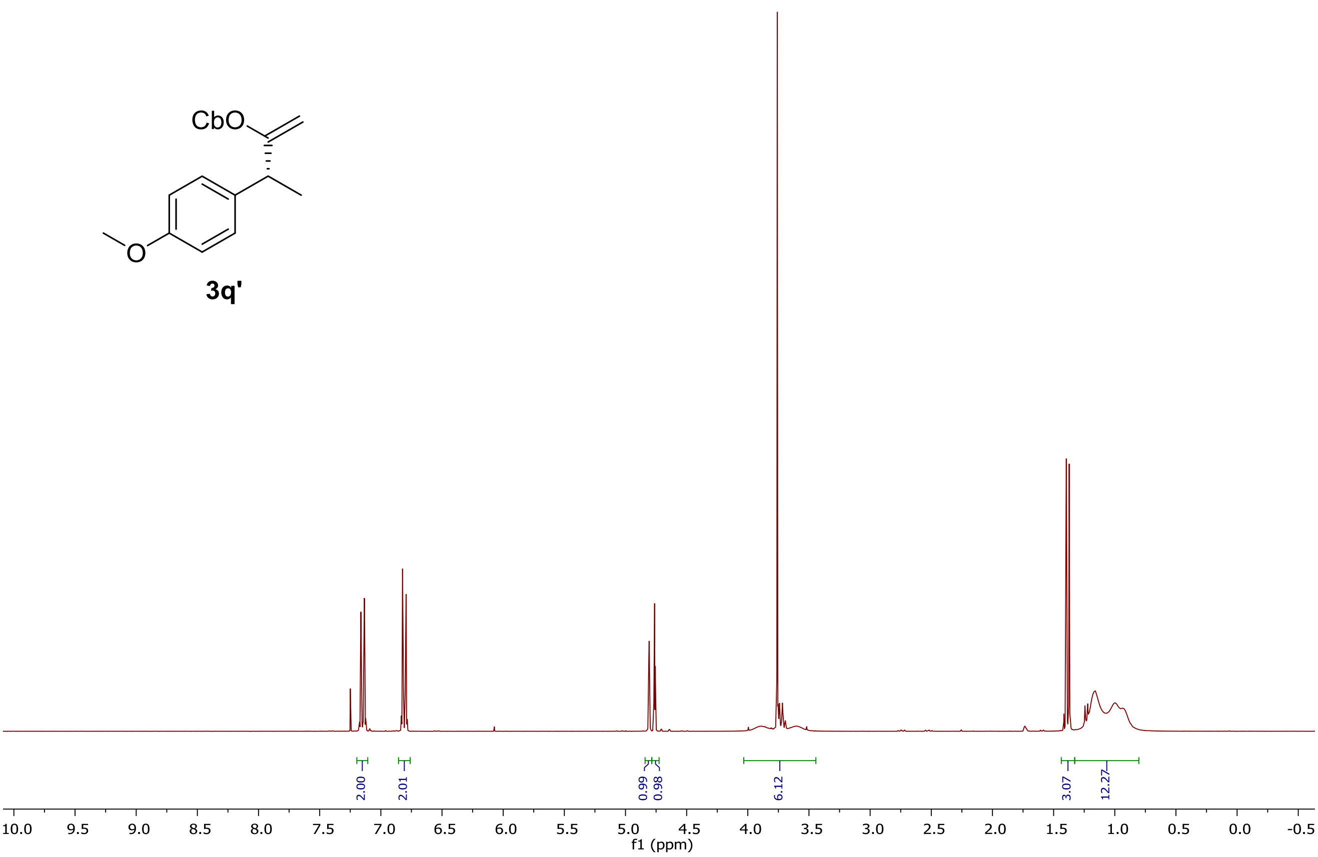

va/yw19836-wyh-3-24-f-s
single pulse decoupled gated NOE

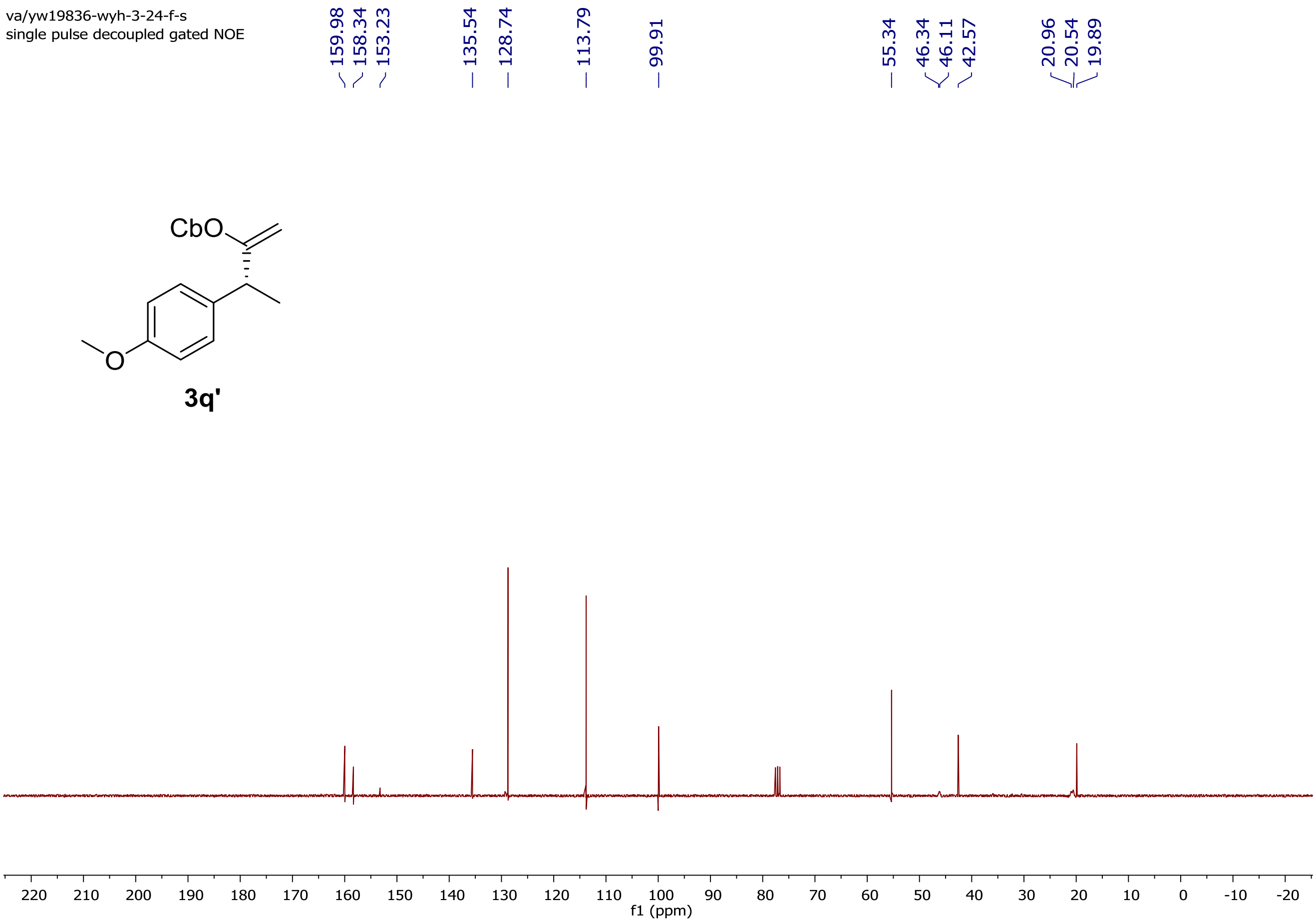

va/yw41506-wyh-3-24
single_pulse

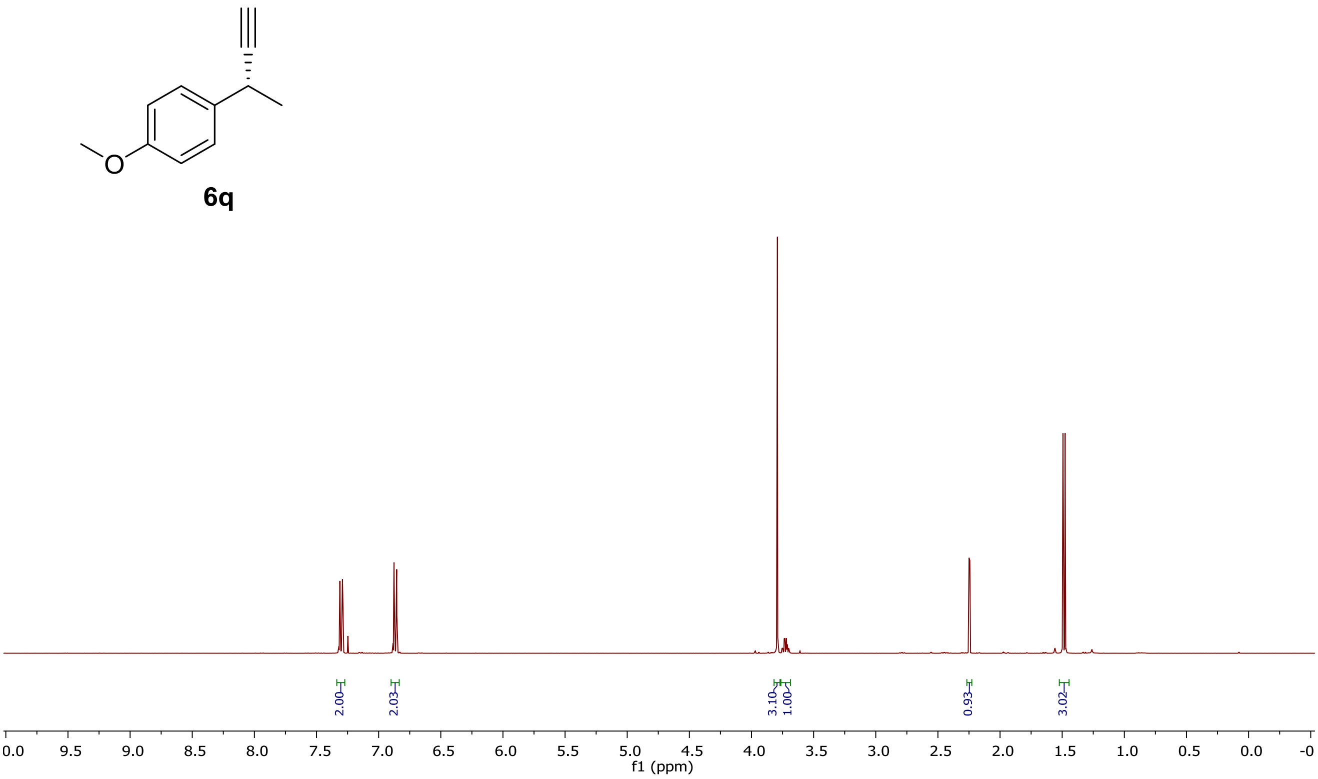

va/yw41506-wyh-3-24
single pulse decoupled gated NOE

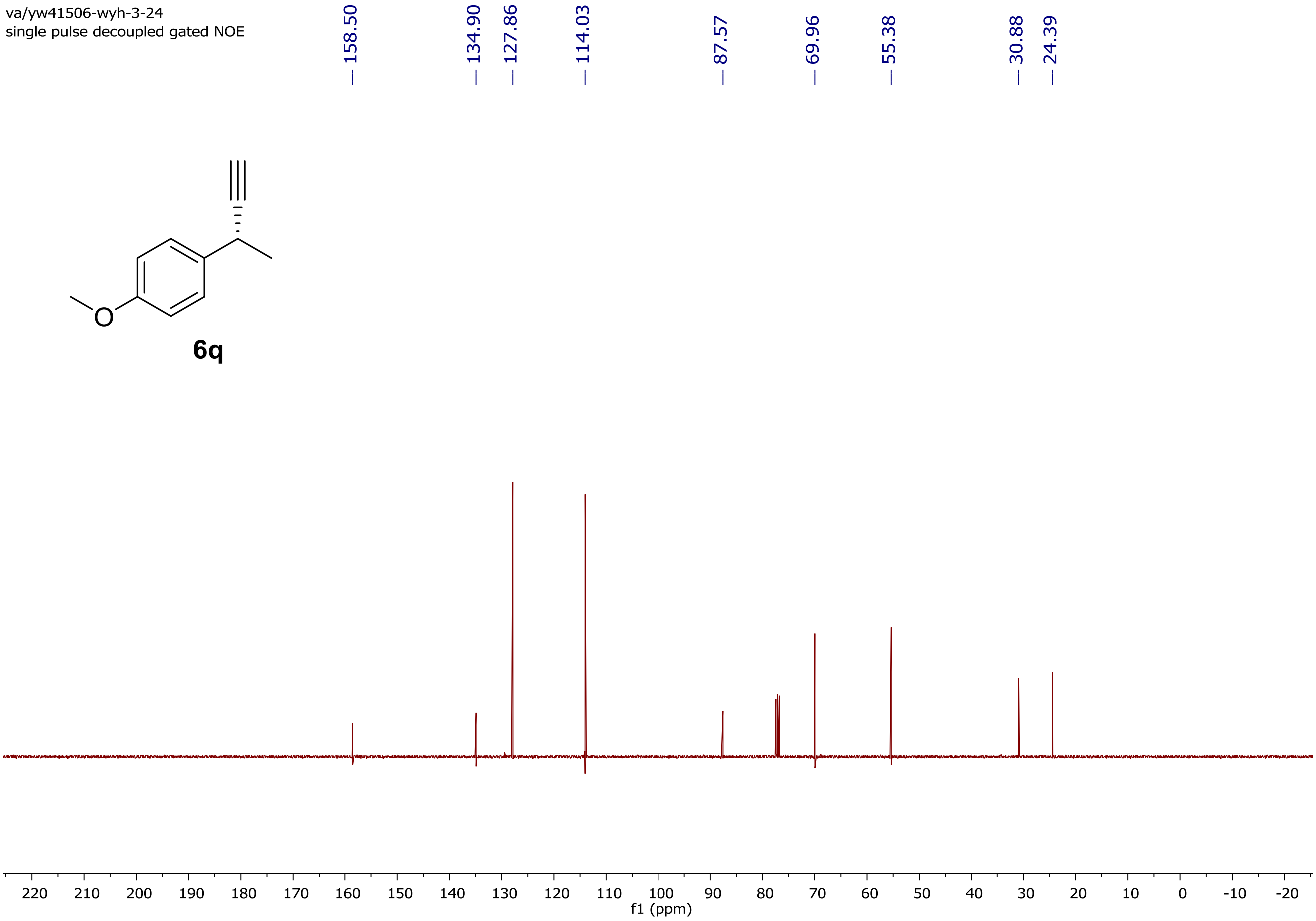

va/yw36975-wyh-2-136
single_pulse

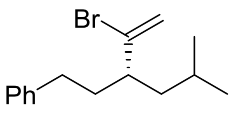

3a

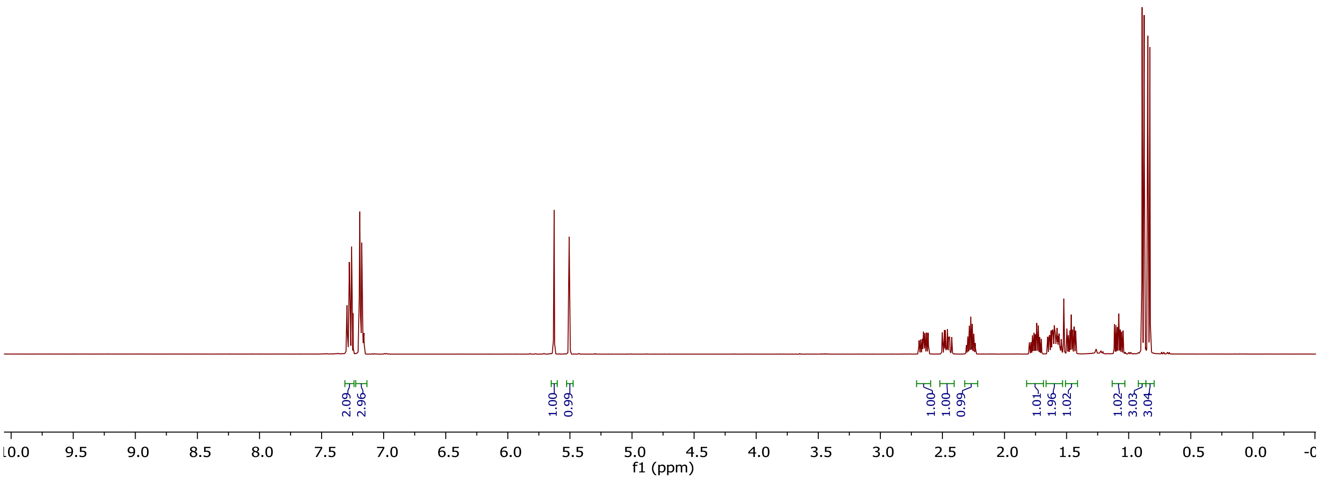

va/yw36975-wyh-2-136
single pulse decoupled gated NOE

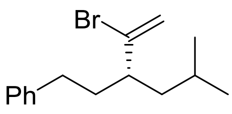

3a

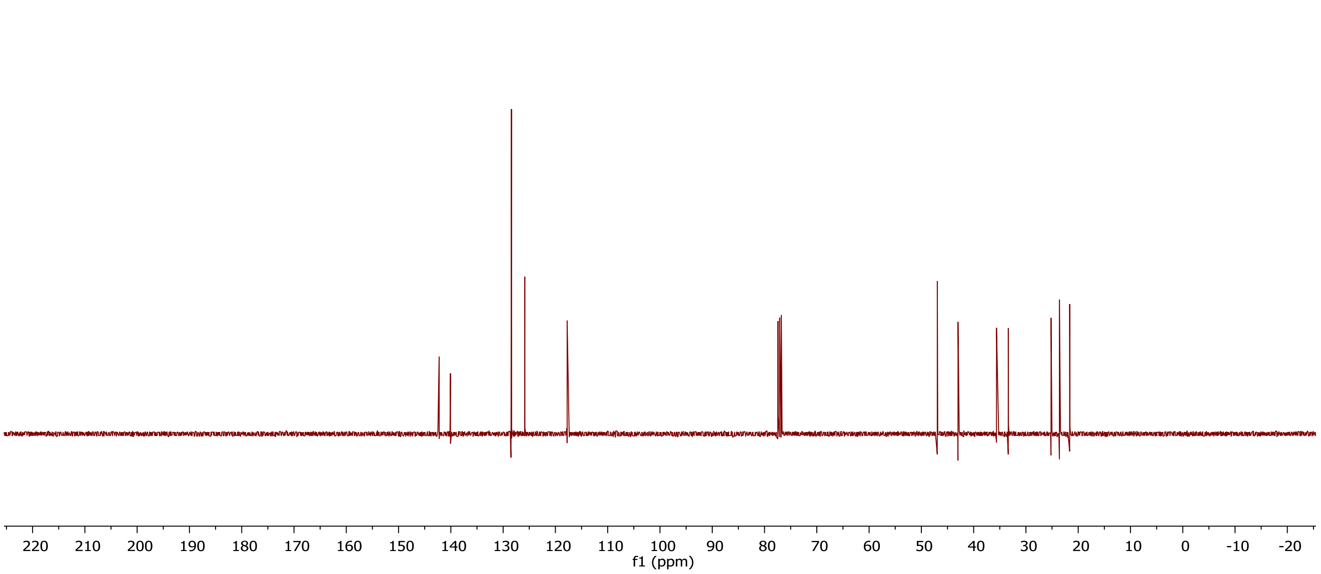

va/yw36995-wyh-2-137
single_pulse

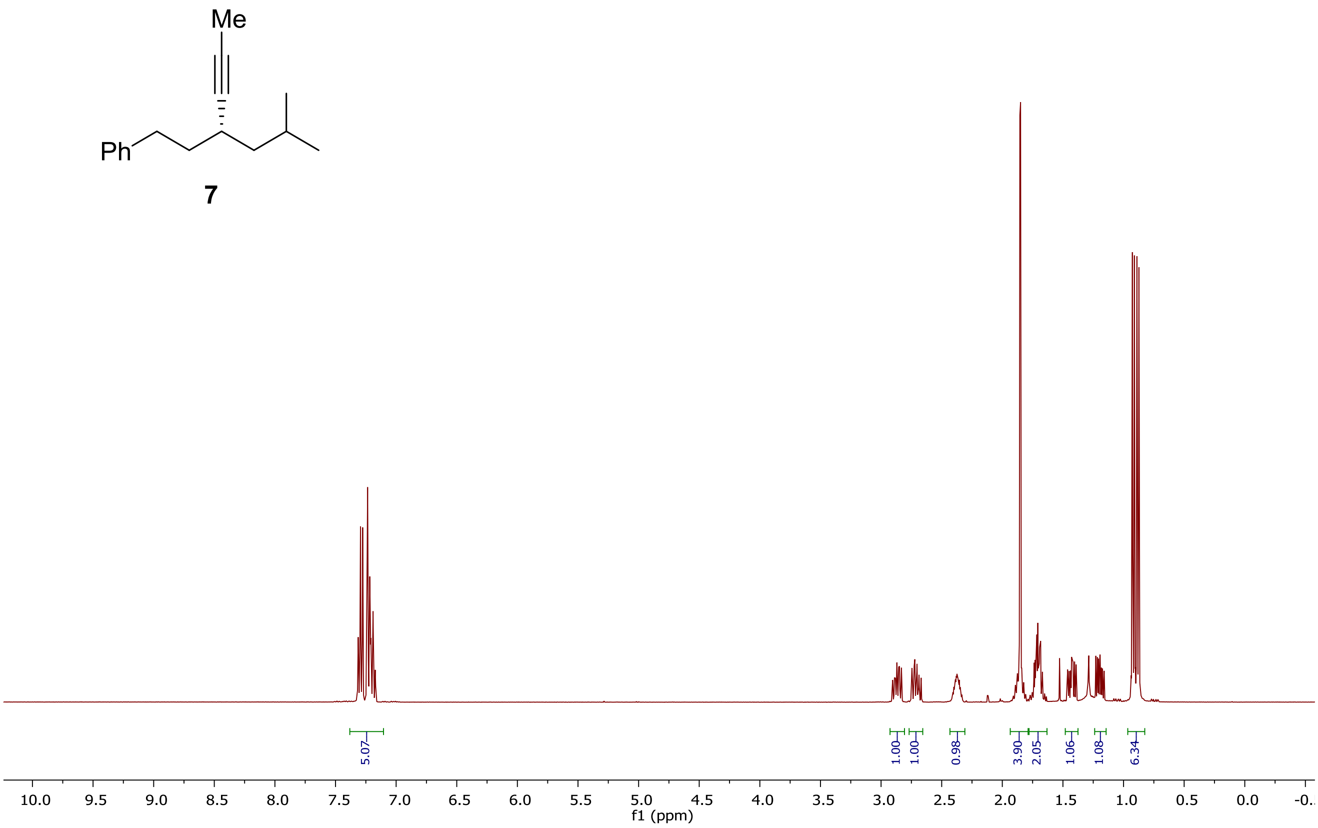

va/yw36995-wyh-2-137
single pulse decoupled gated NOE

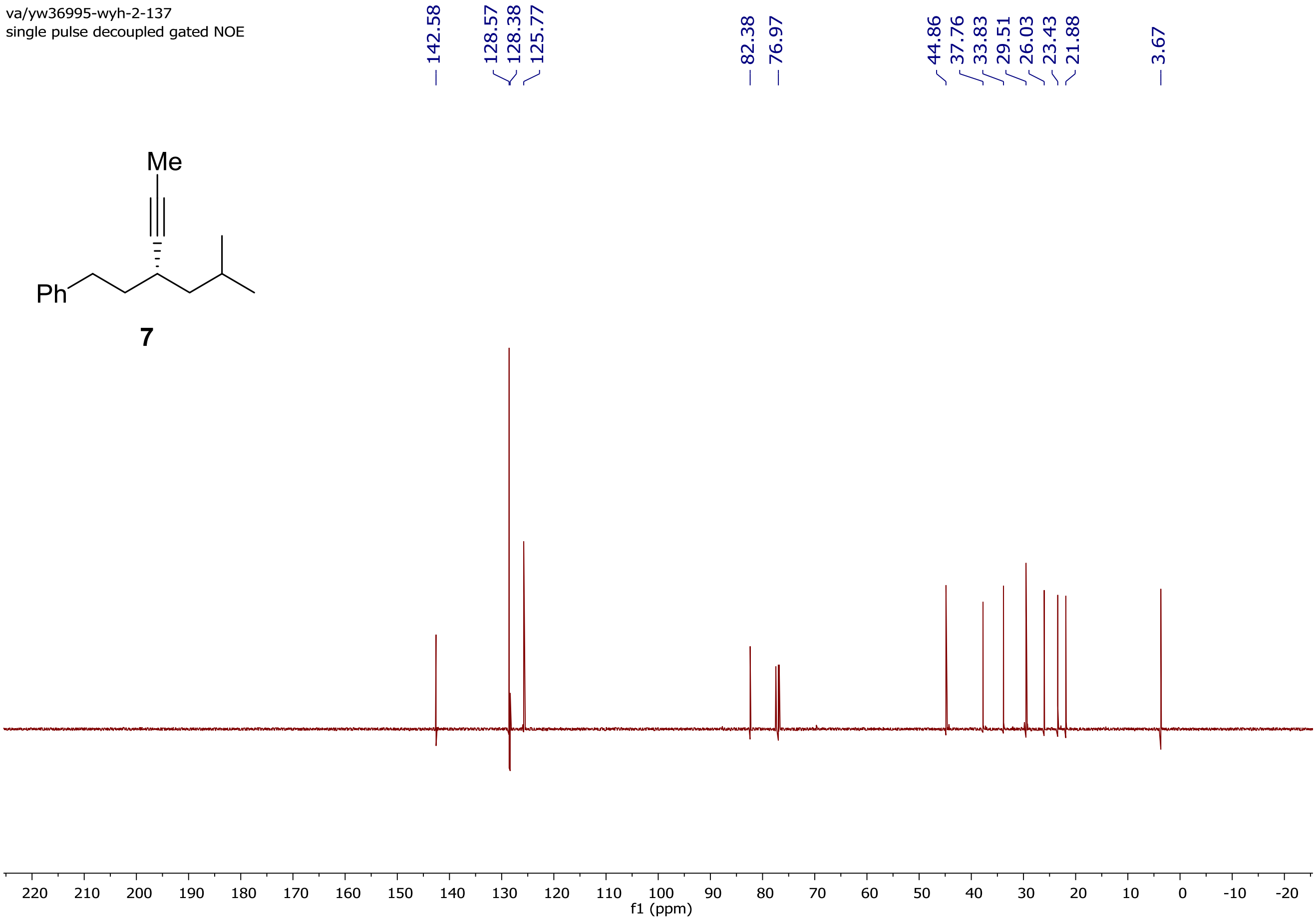

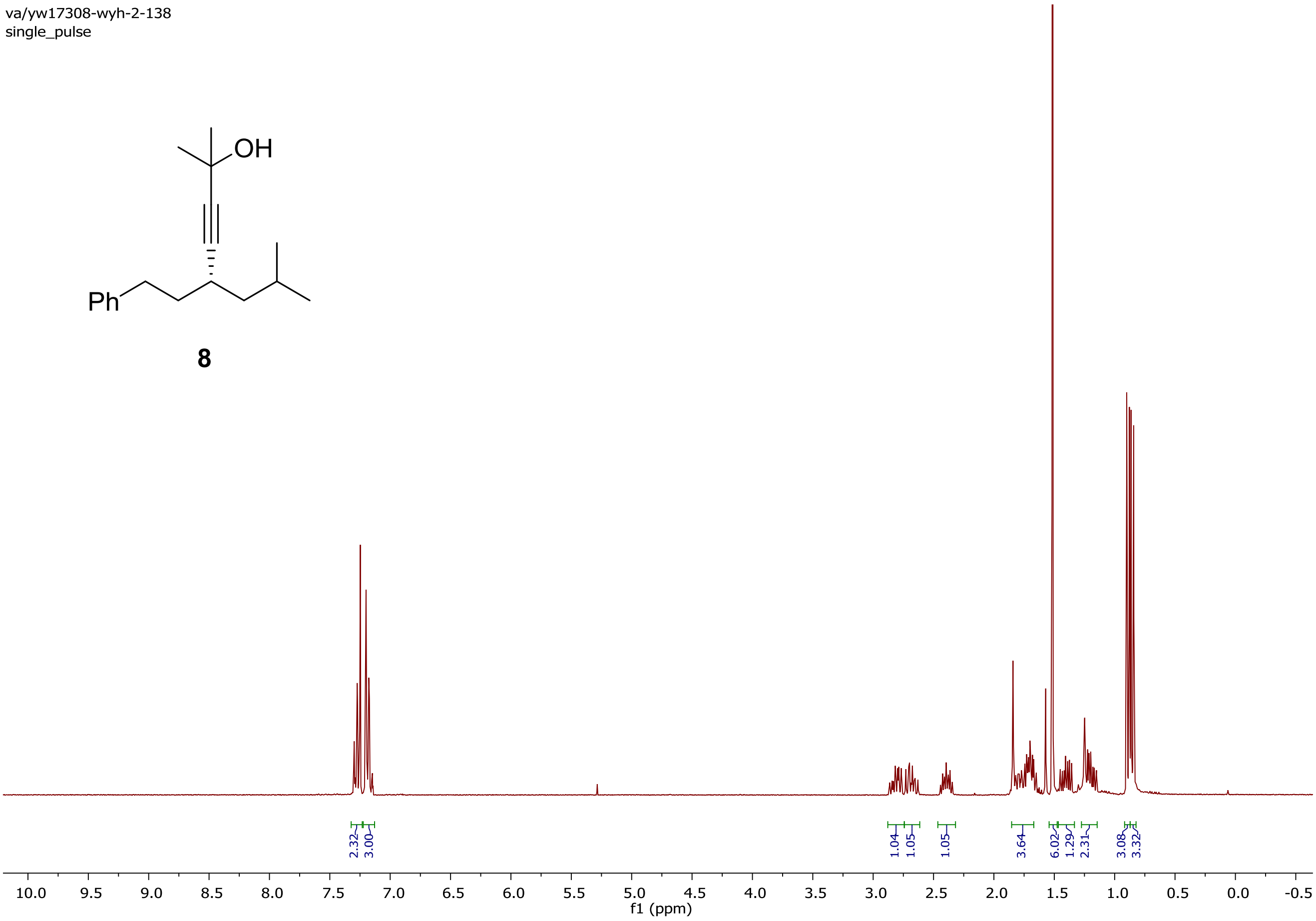
va/yw17308-wyh-2-138
single_pulse
OH
Ph
8
2.32
3.00
1.04
1.05
1.05
3.64
6.02
1.29
2.31
3.08
3.32
10.0 9.5 9.0 8.5 8.0 7.5 7.0 6.5 6.0 5.5 5.0 4.5 4.0 3.5 3.0 2.5 2.0 1.5 1.0 0.5 0.0 -0.5
f1 (ppm)

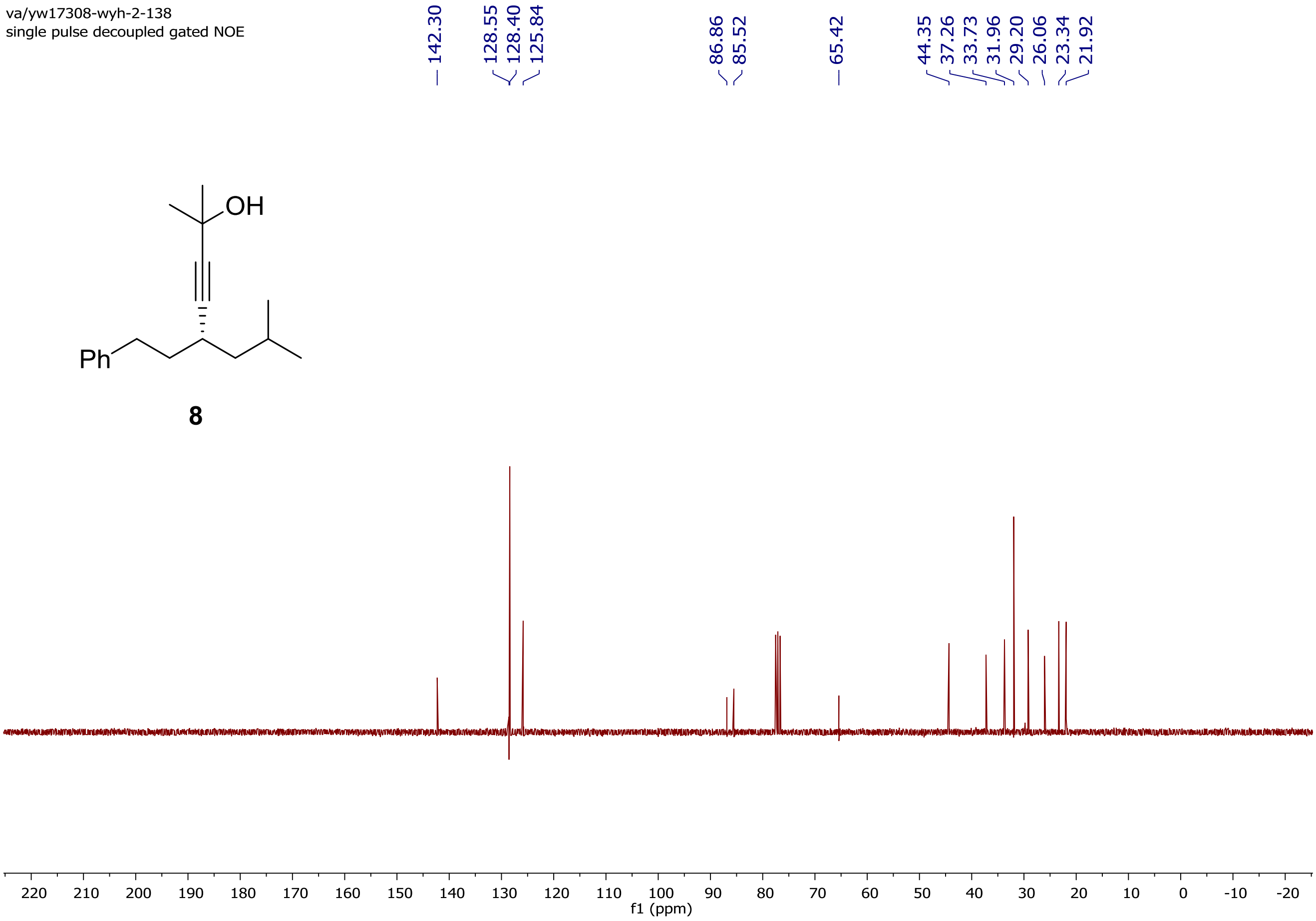
va/yw17308-wyh-2-138
single pulse decoupled gated NOE
142.30
128.55
128.40
125.84
86.86
85.52
65.42
44.35
37.26
33.73
31.96
29.20
26.06
23.34
21.92
OH
Ph
8
220 210 200 190 180 170 160 150 140 130 120 110 100 90 80 70 60 50 40 30 20 10 0 -10 -20
f1 (ppm)

va/yw17310-wyh-2-139
single_pulse

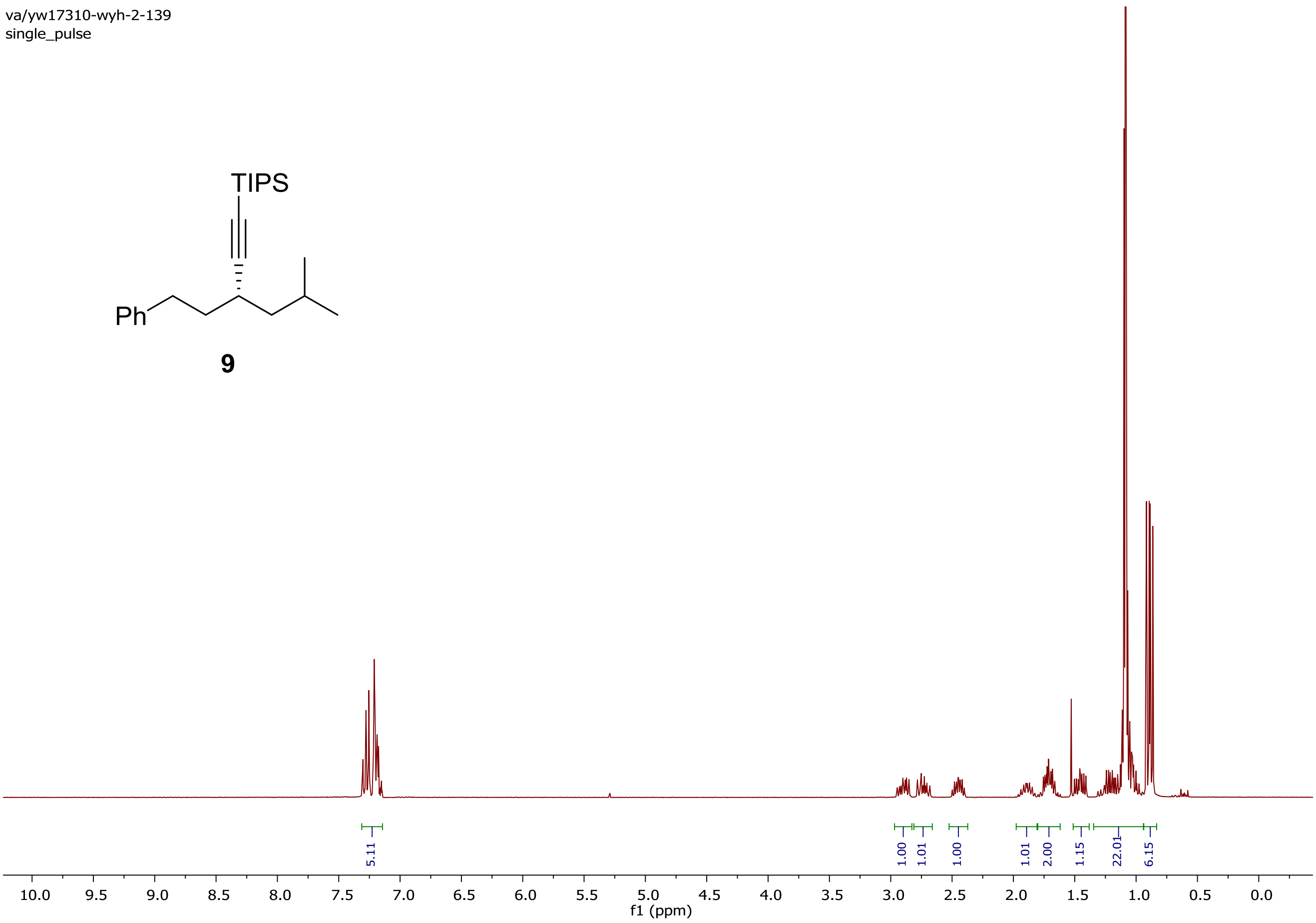

va/yw17310-wyh-2-139
single pulse decoupled gated NOE

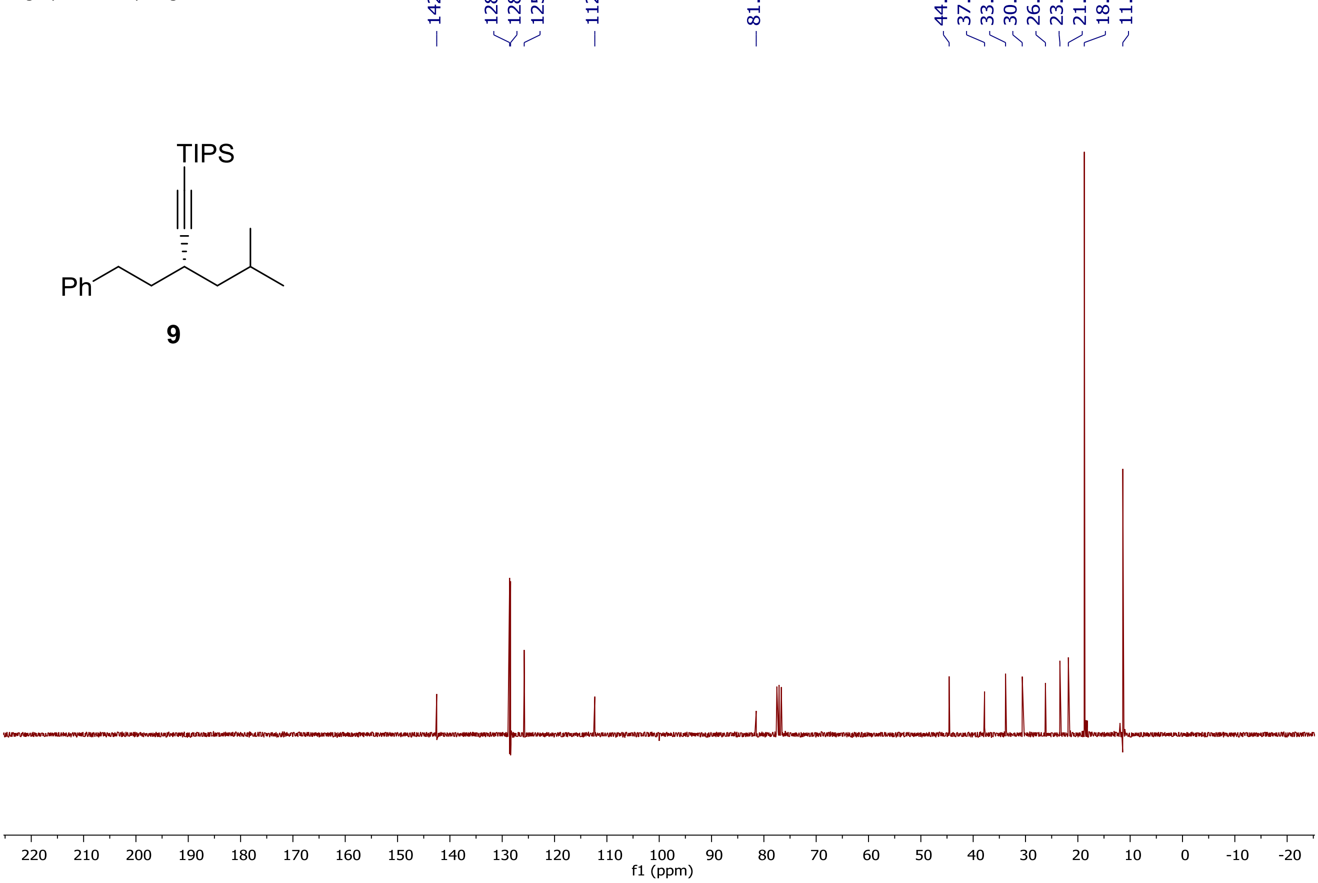

Supplement: Supplementary file 1 — Supplementary [file ANIE-55-4270-s001.pdf]
